# Supplementary material for: 5-Oxo-hexahydroquinoline Derivatives and Their Tetrahydroquinoline Counterparts as Multidrug Resistance Reversal Agents
Source: Molecules. 2020 Apr 16;25(8):1839. doi: 10.3390/molecules25081839 (PMC7221826; doi:10.3390/molecules25081839)
Supplement: Supplementary file 1 [file molecules-25-01839-s001.pdf]

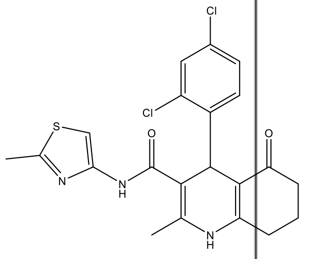

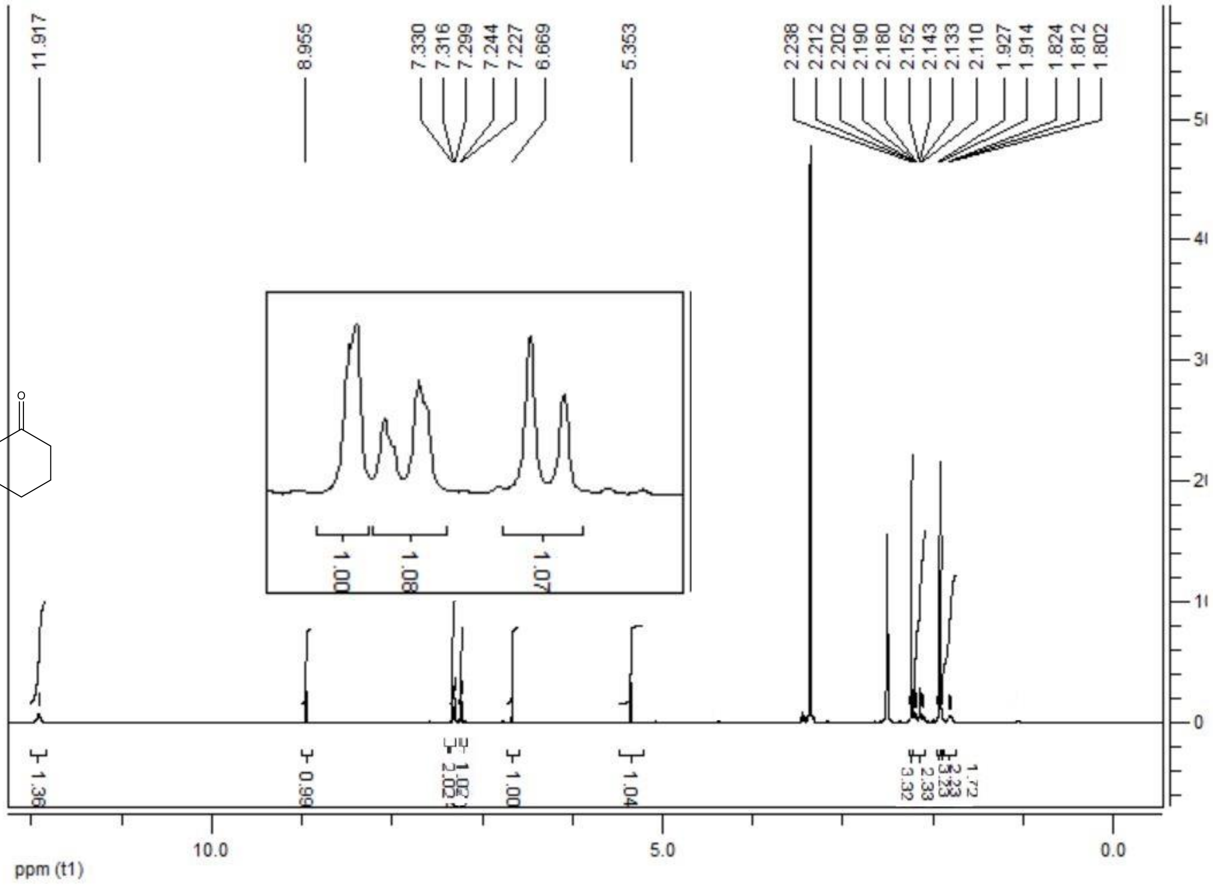

Figure 1s. ¹H-NMR spectrum of A1

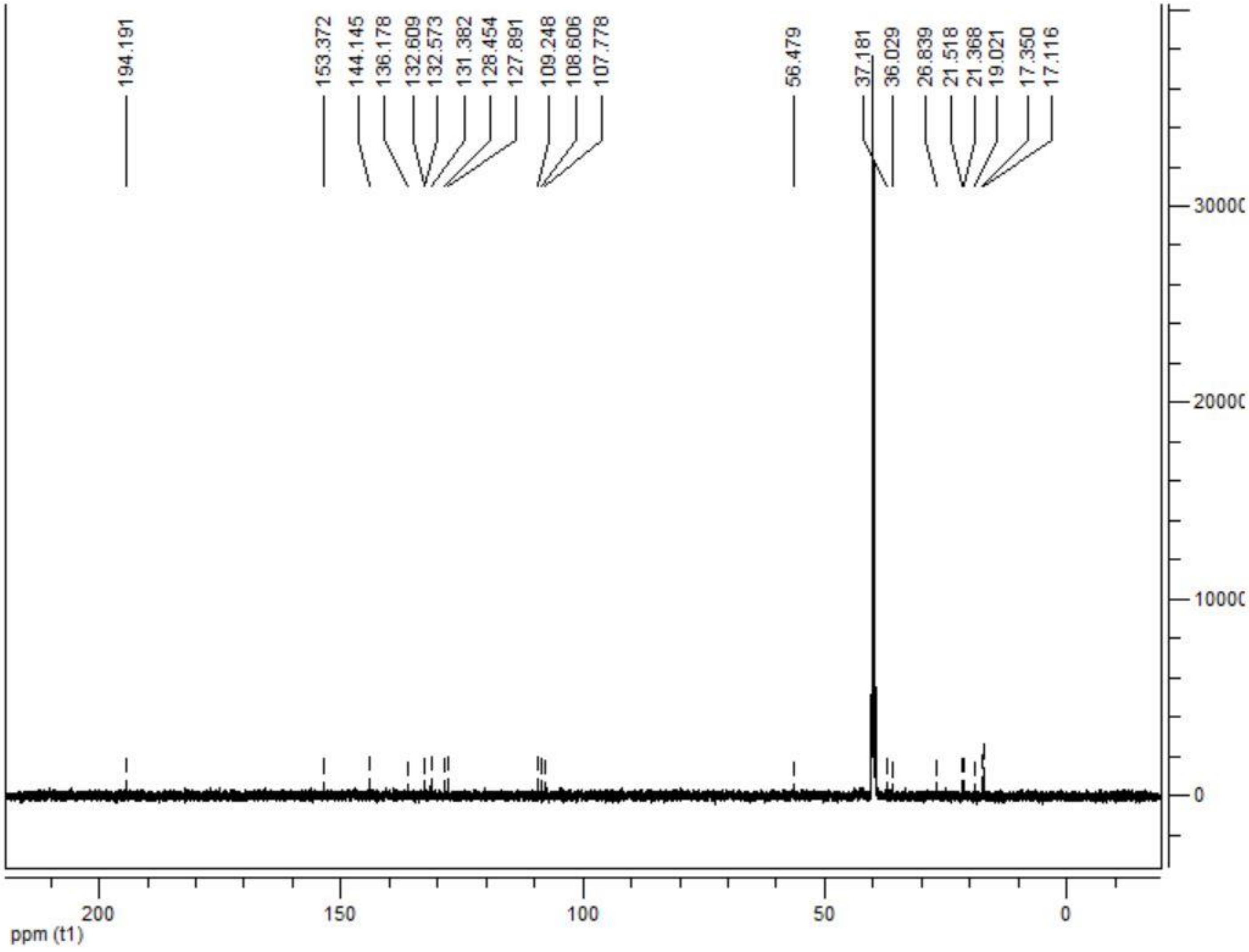

Figure 2s. ^{13}C-NMR spectrum of A1

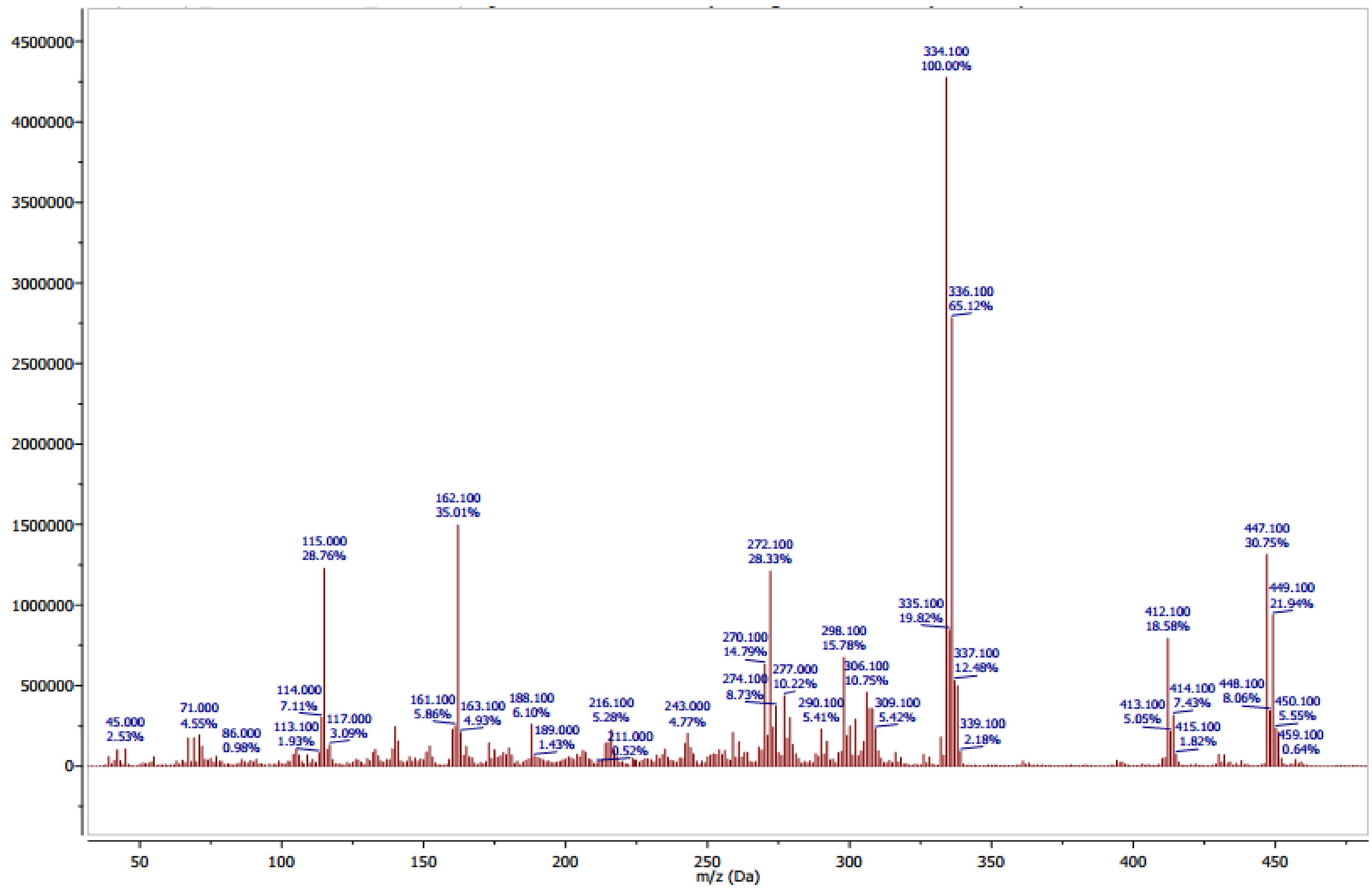

Figure 3s. Mass spectrum of A1

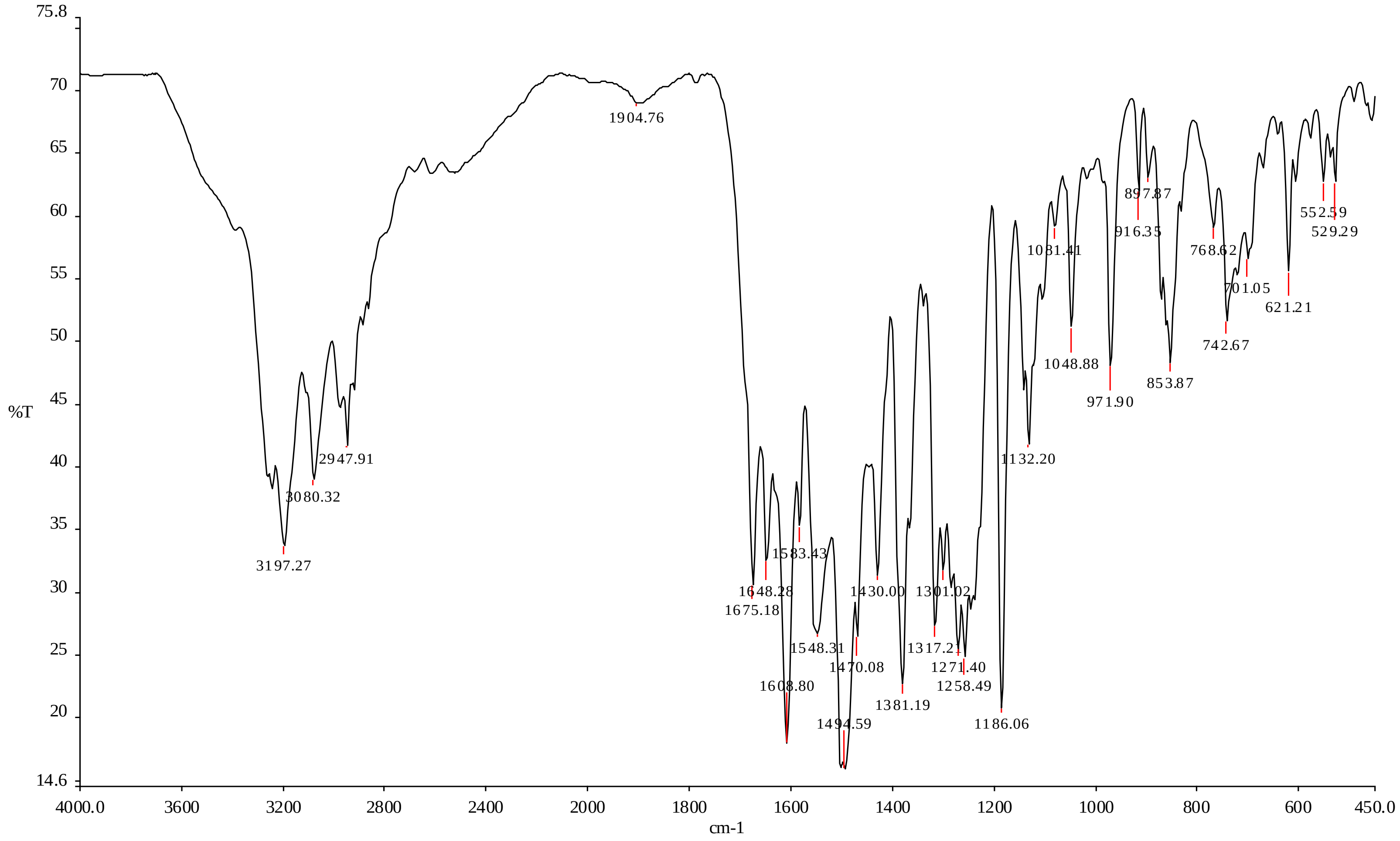

Figure 4s. IR spectrum of A1

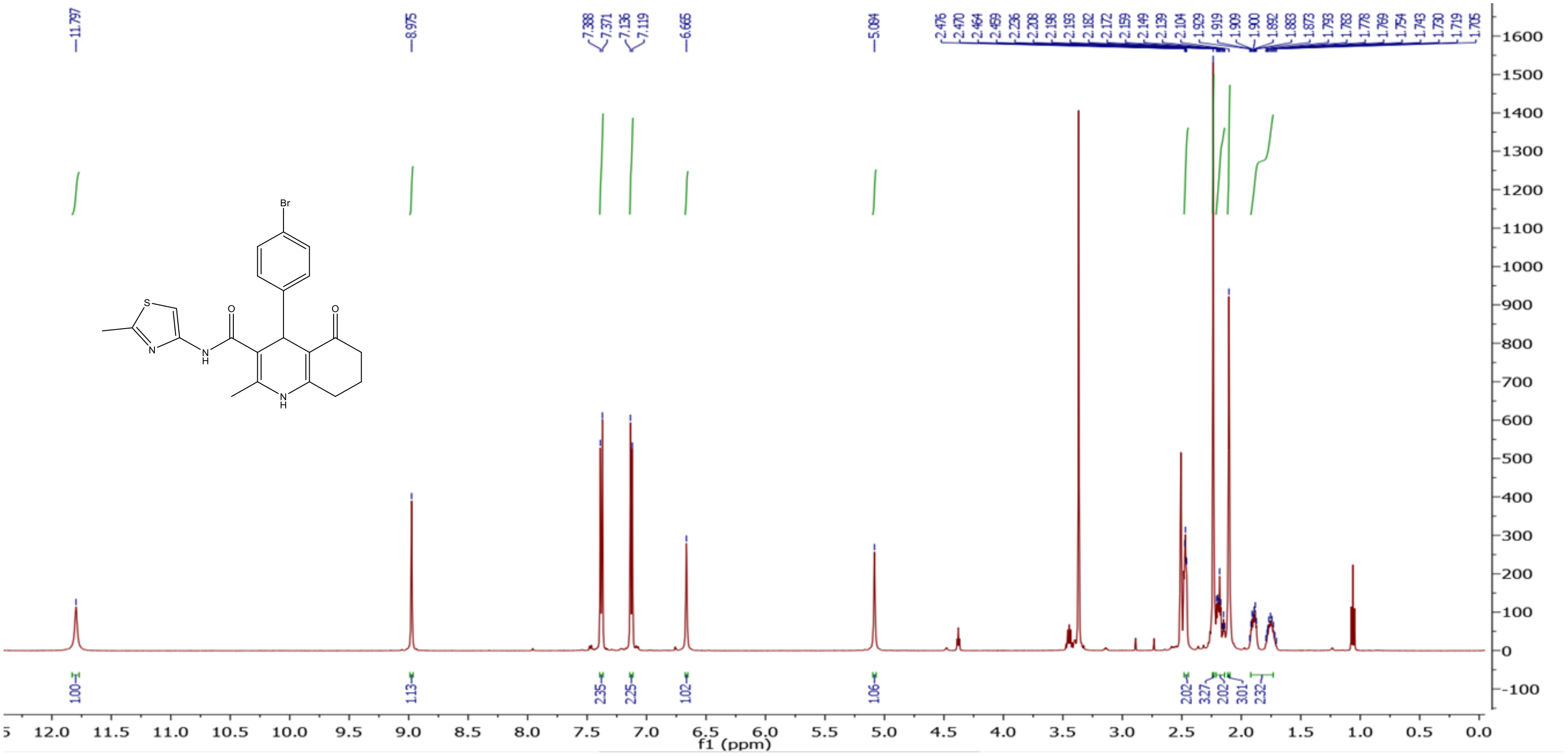

Figure 5s. ^{1}H-NMR spectrum of A2

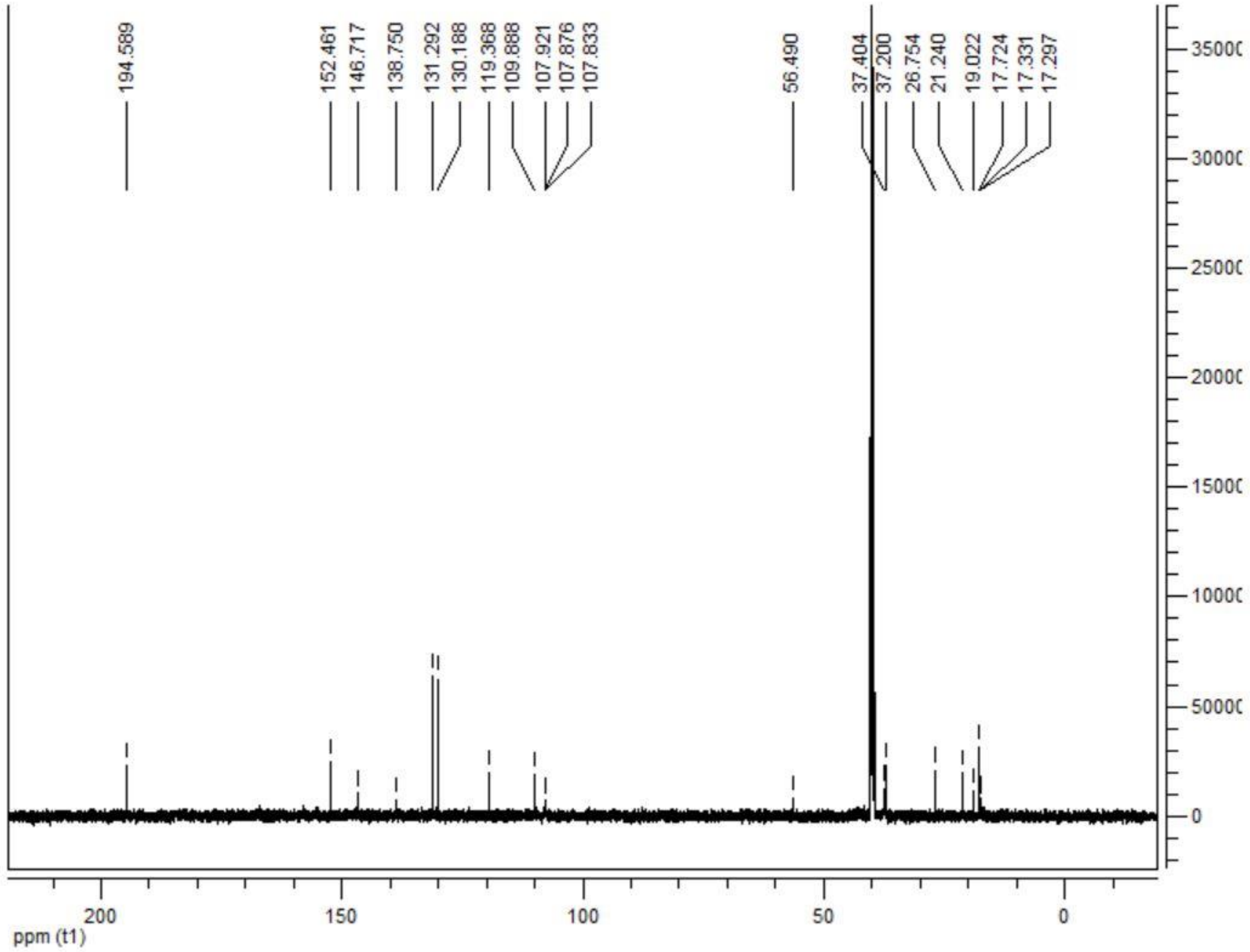

Figure 6s. ^{13}C-NMR spectrum of A2

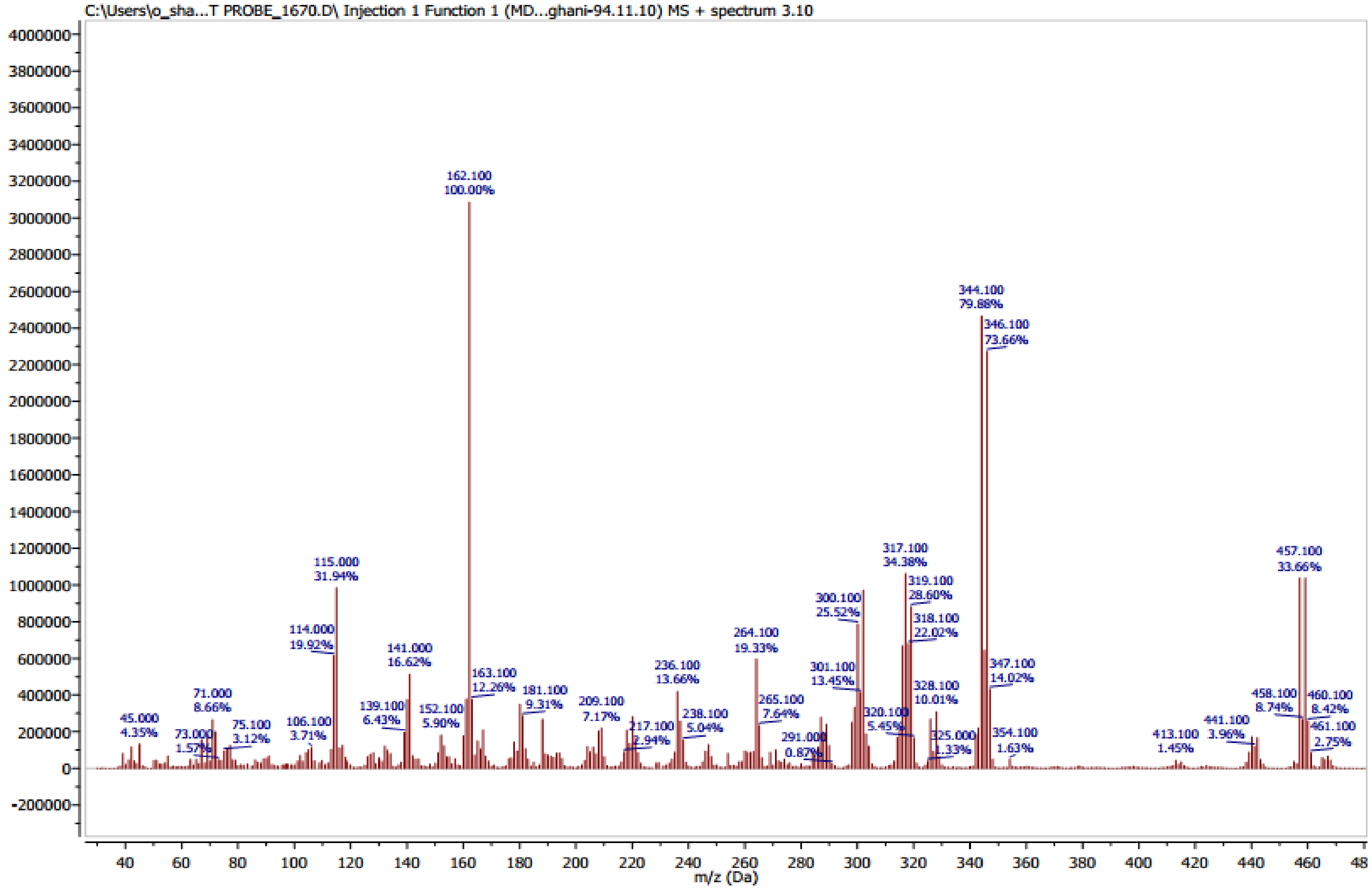

Figure 7s. Mass spectrum of A2

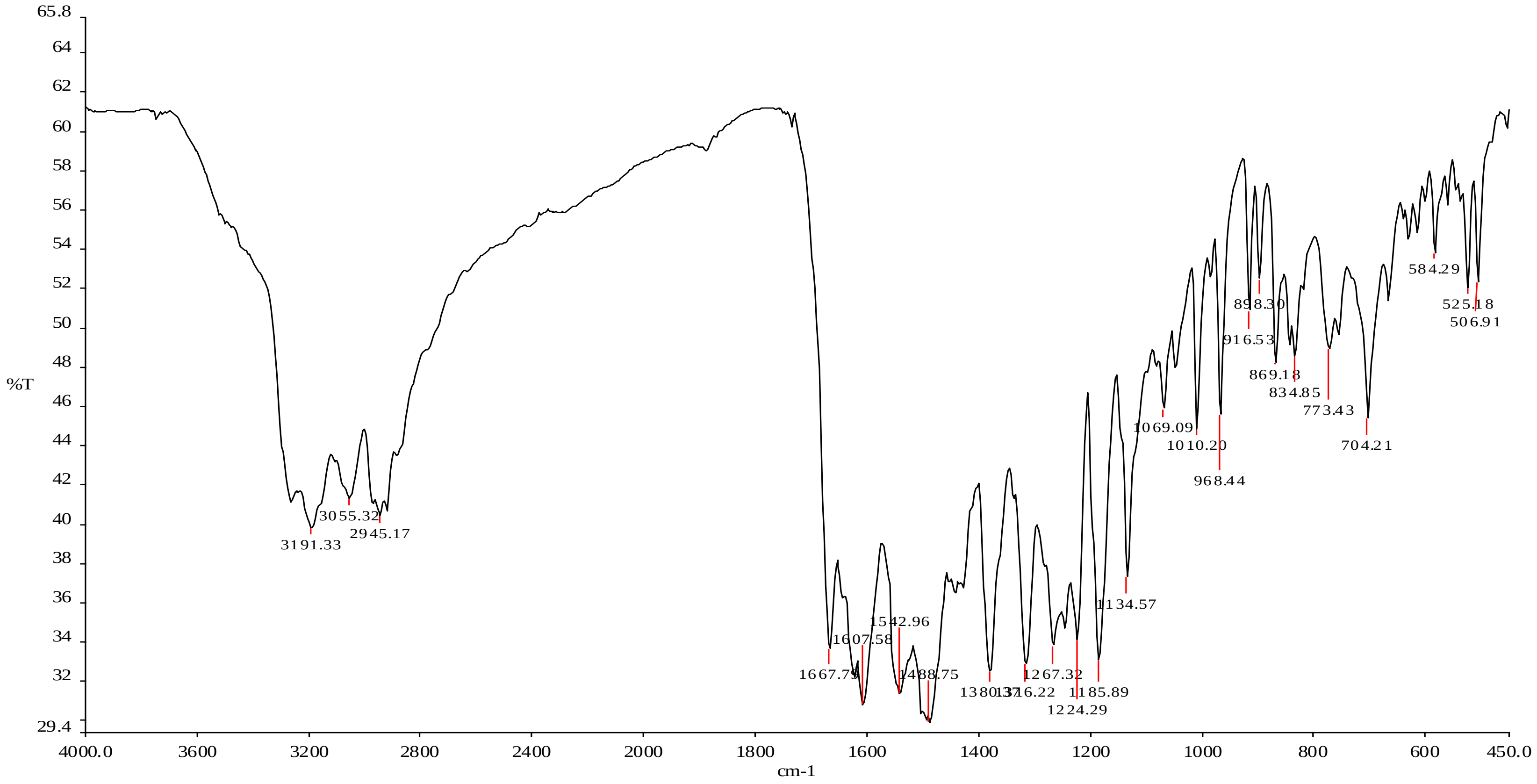

Figure 8s. IR spectrum of A2

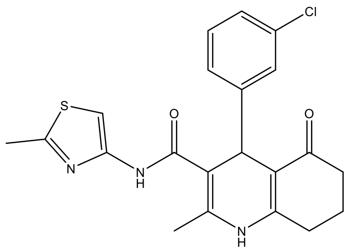

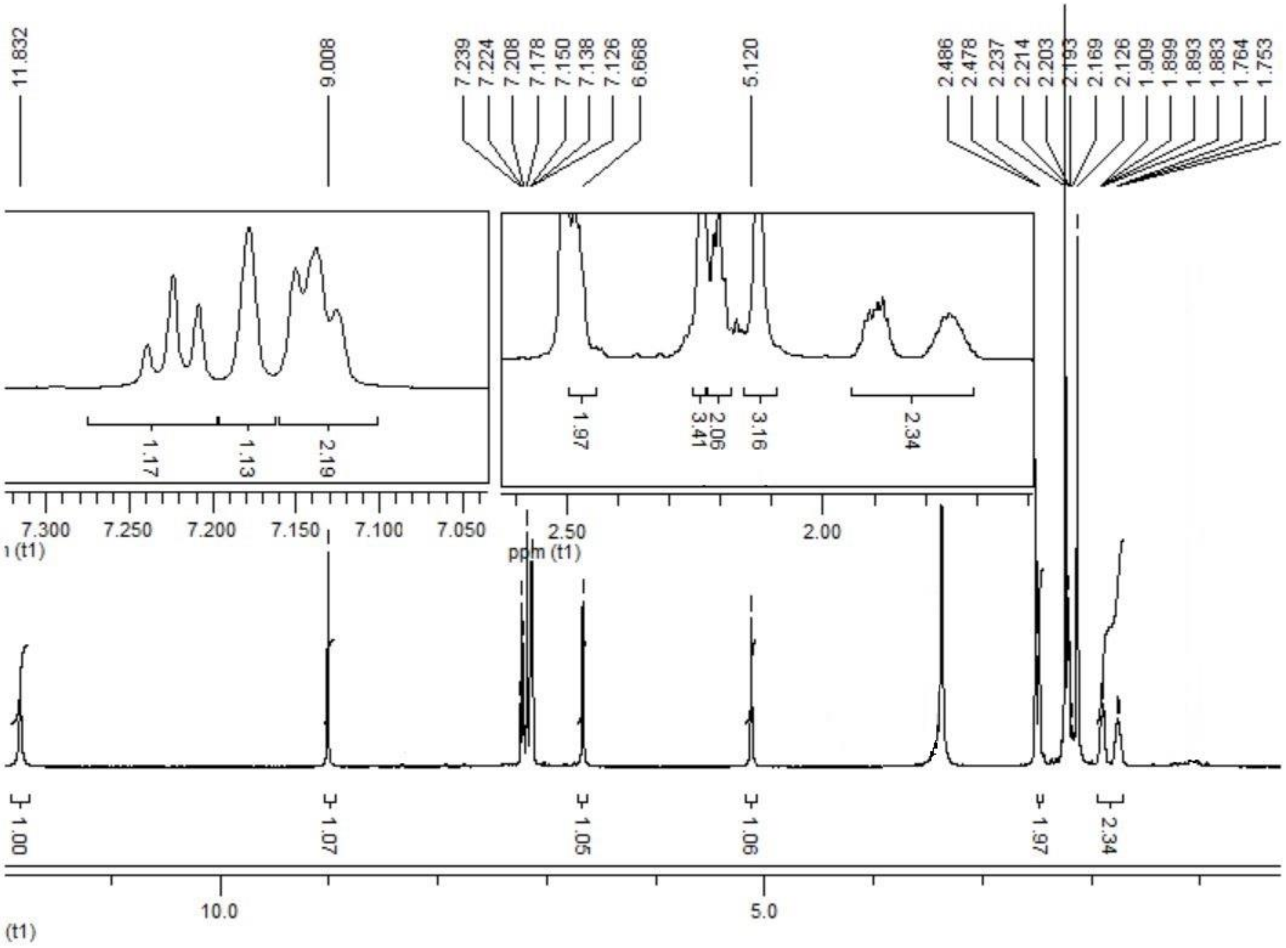

Figure 9s. ^{1}H-NMR spectrum of A3

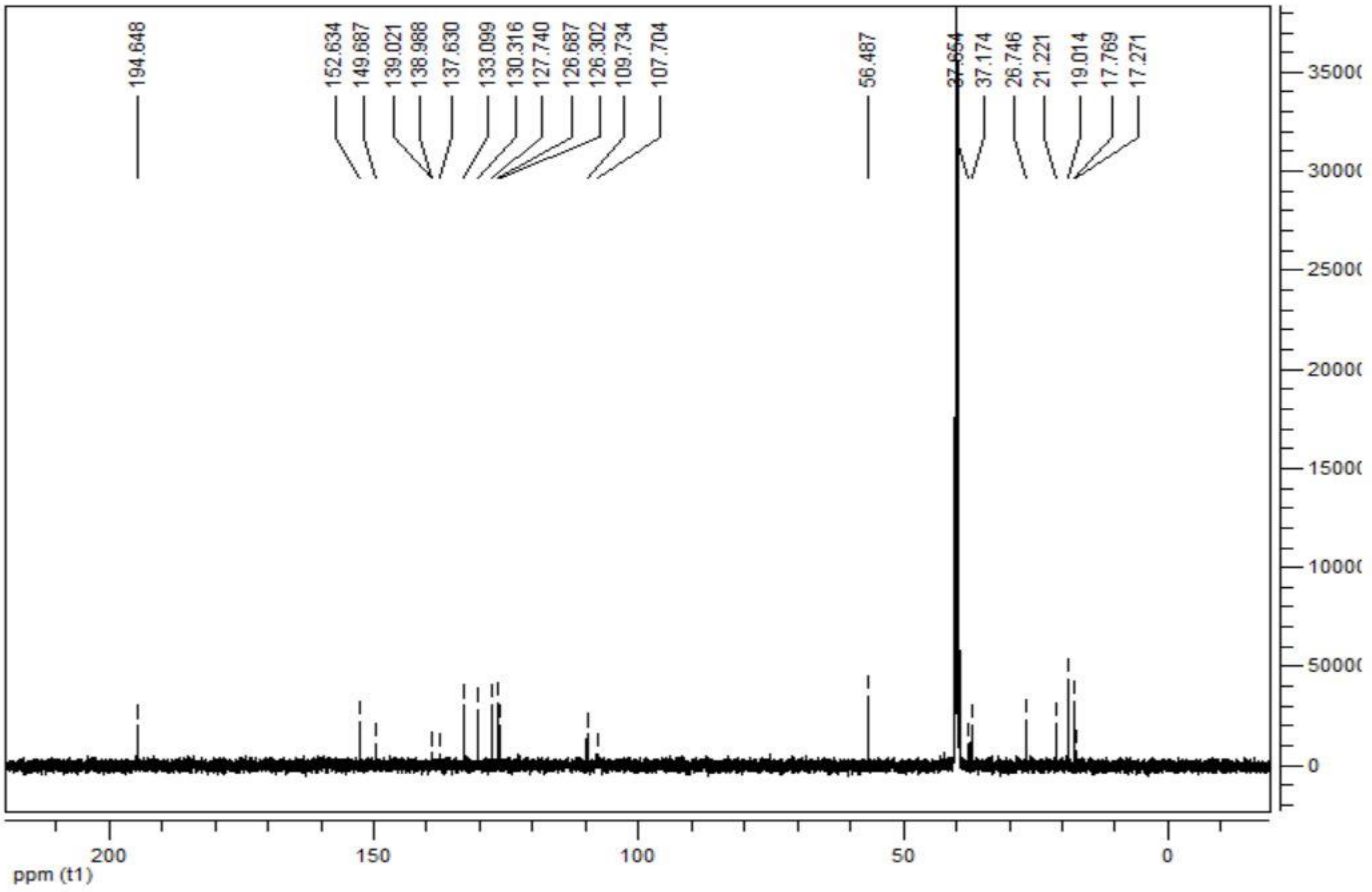

Figure 10s. ^{13}C-NMR spectrum of A3

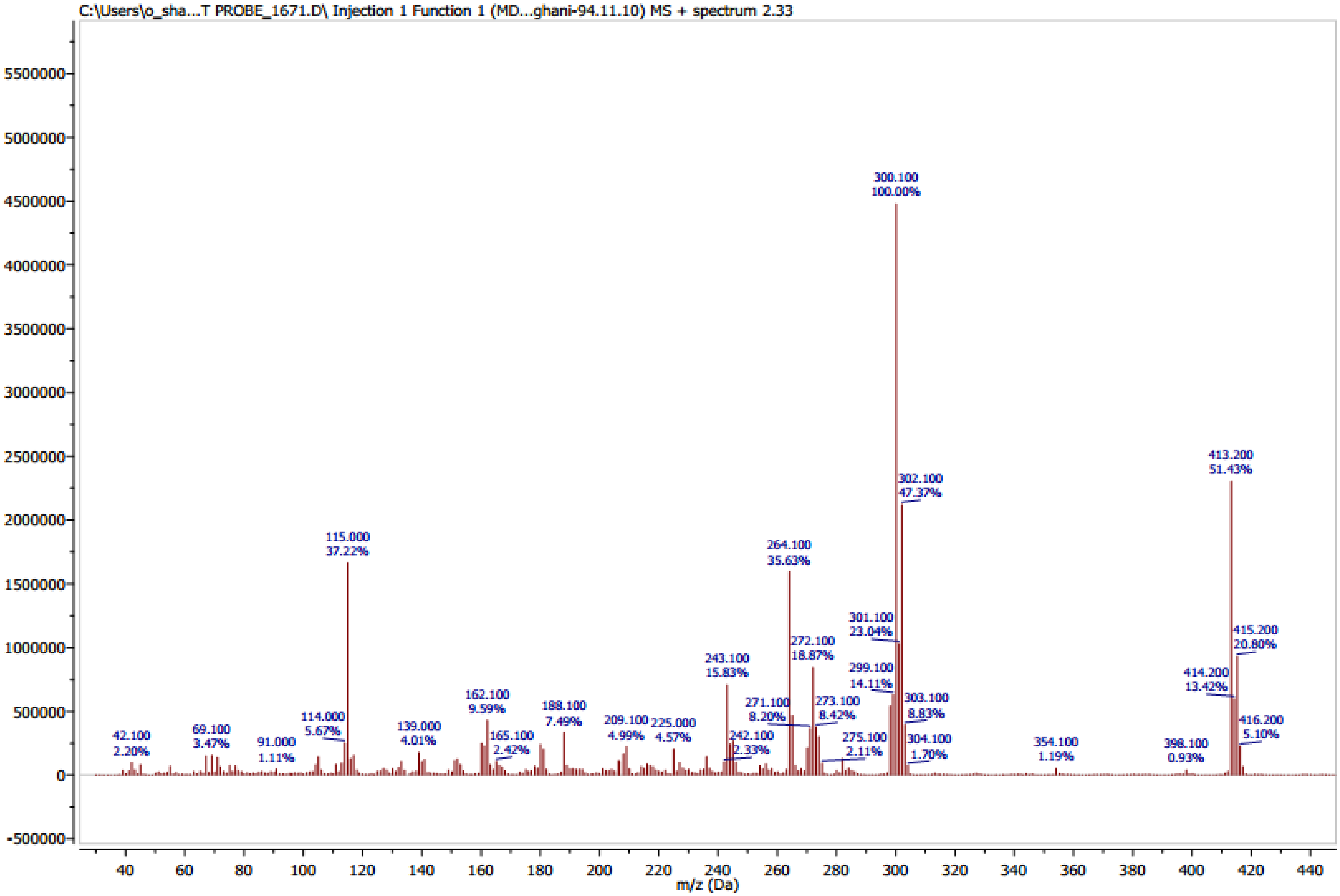

Figure 11s. Mass spectrum of A3

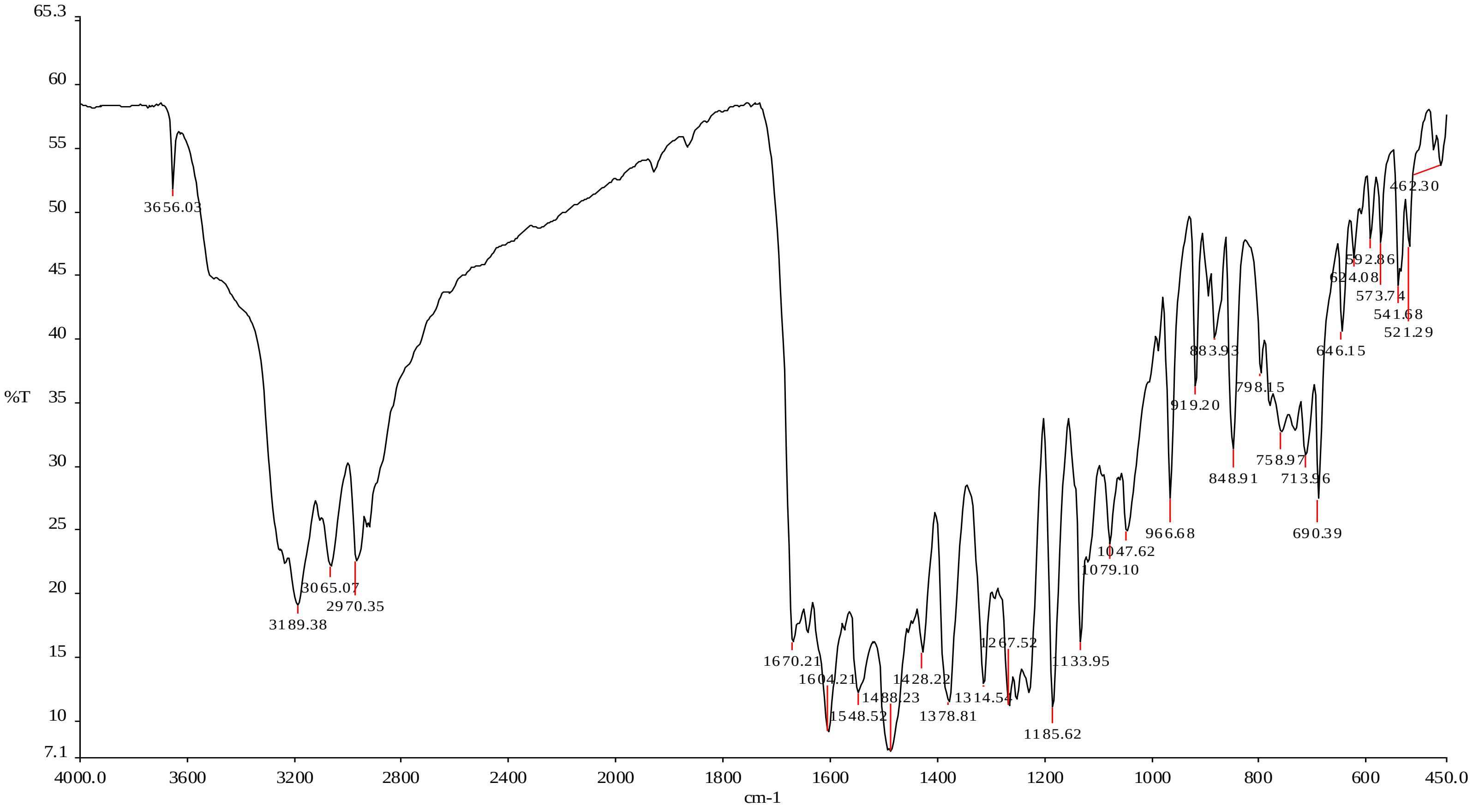

Figure 12s. IR spectrum of A3

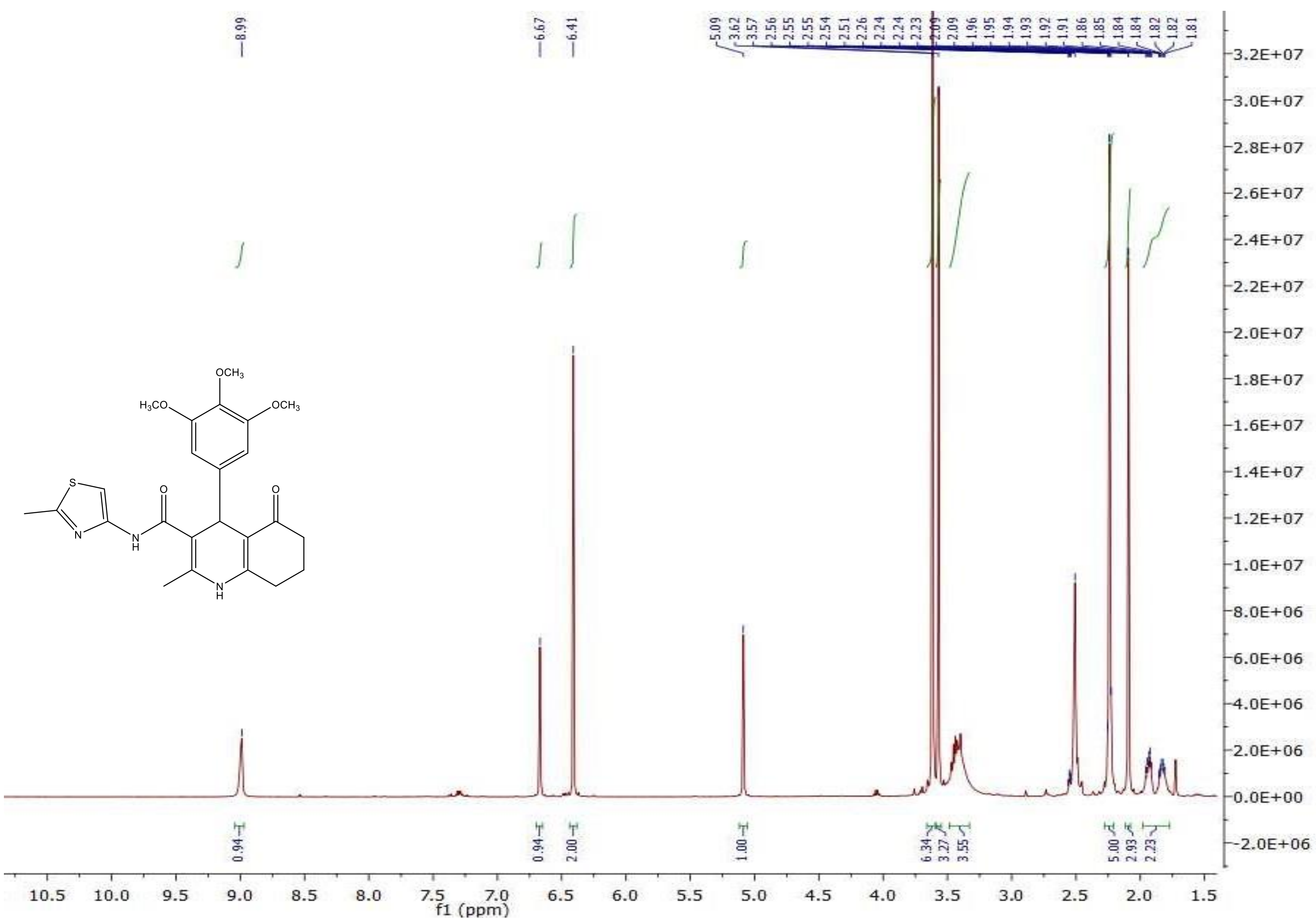

Figure 13s. ^{1}H-NMR spectrum of A4

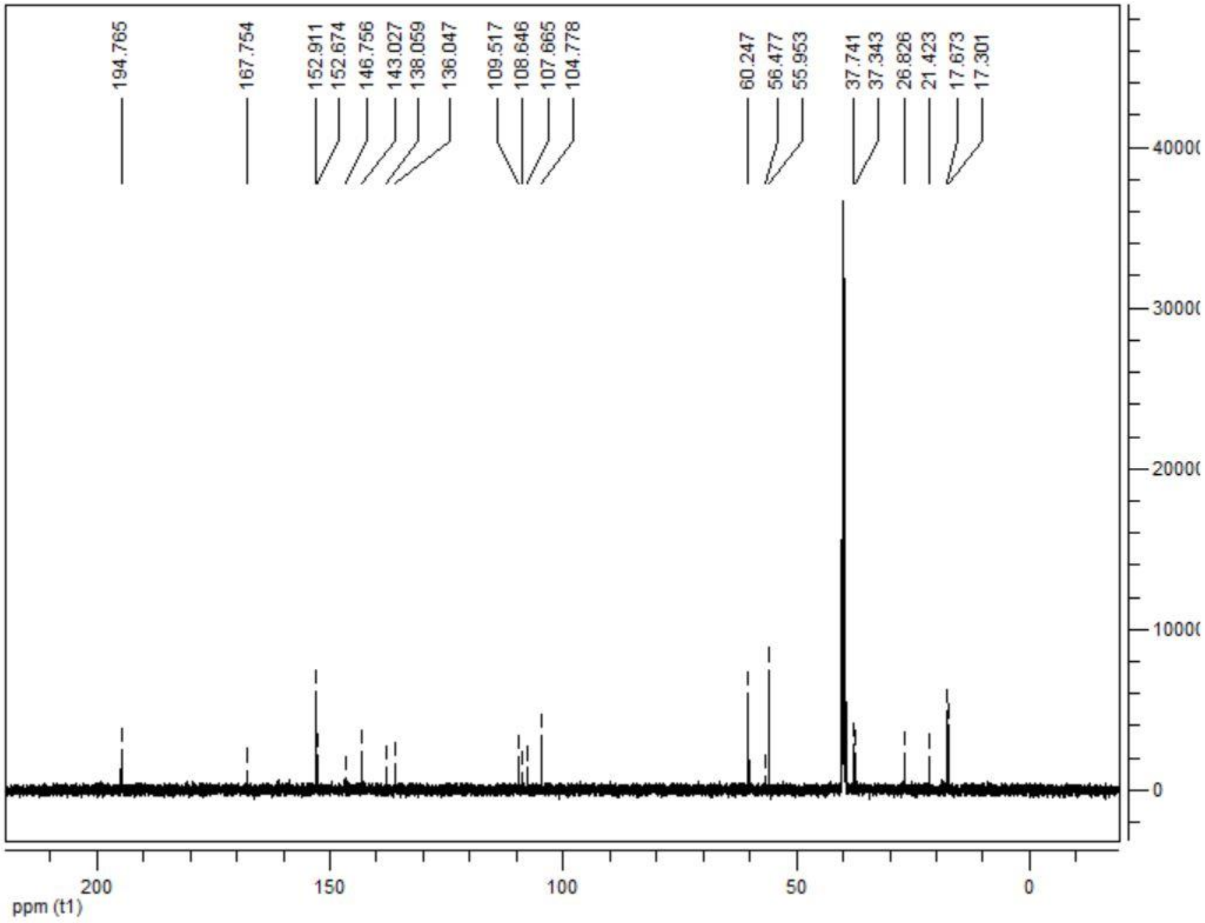

Figure 14s. ^{13}C-NMR spectrum of A4

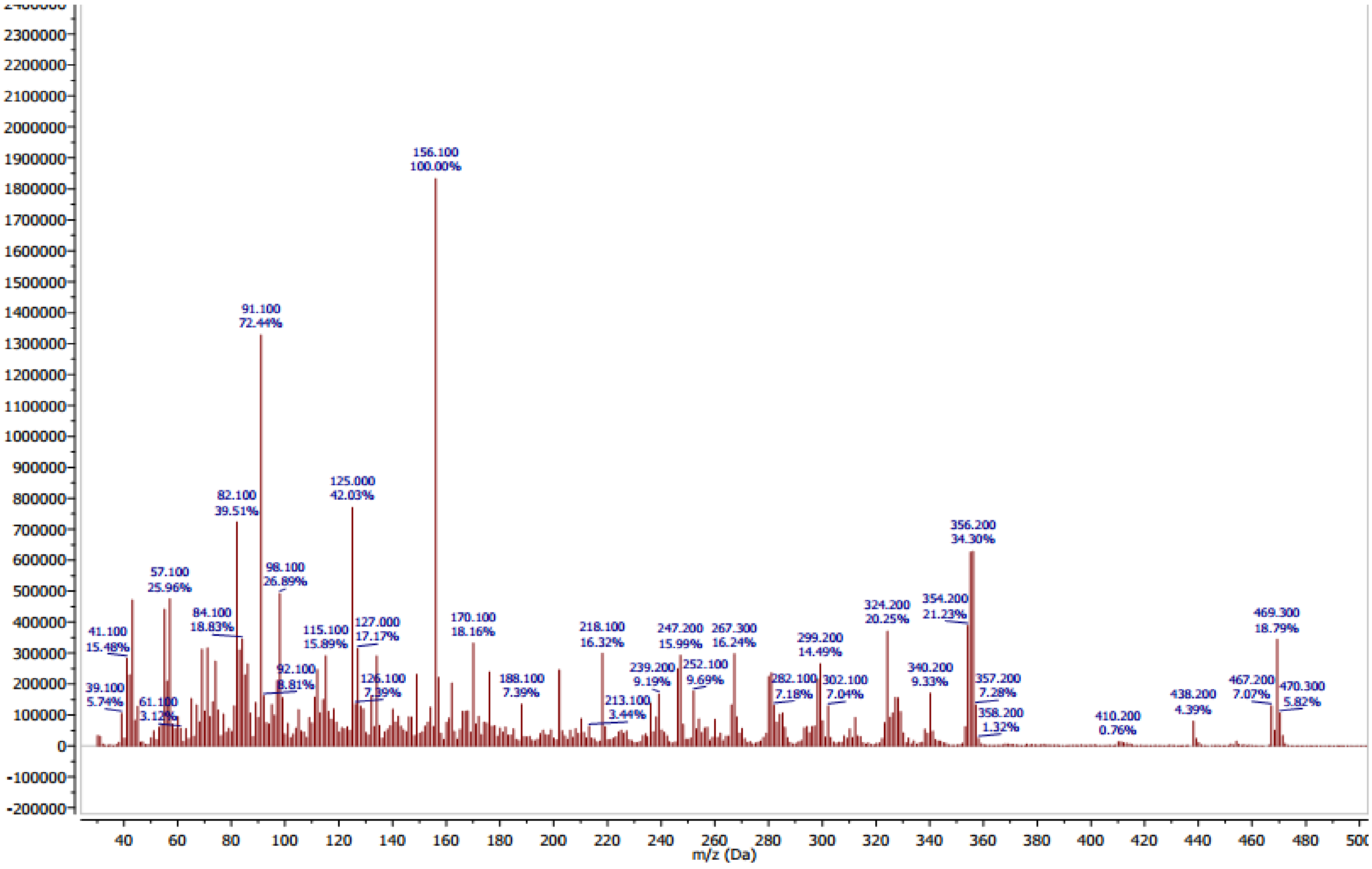

Figure 15s. Mass spectrum of A4

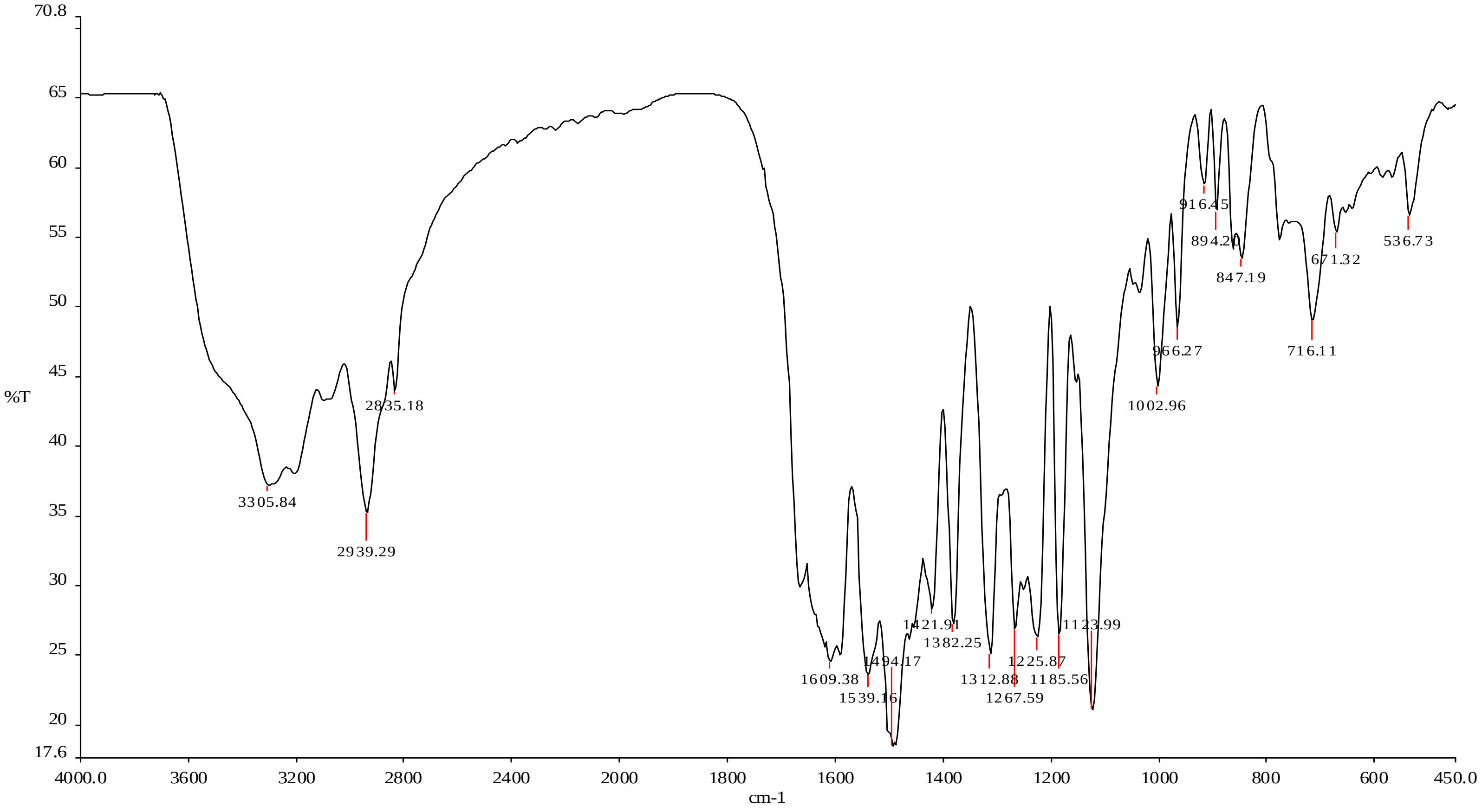

Figure 16s. IR spectrum of A4

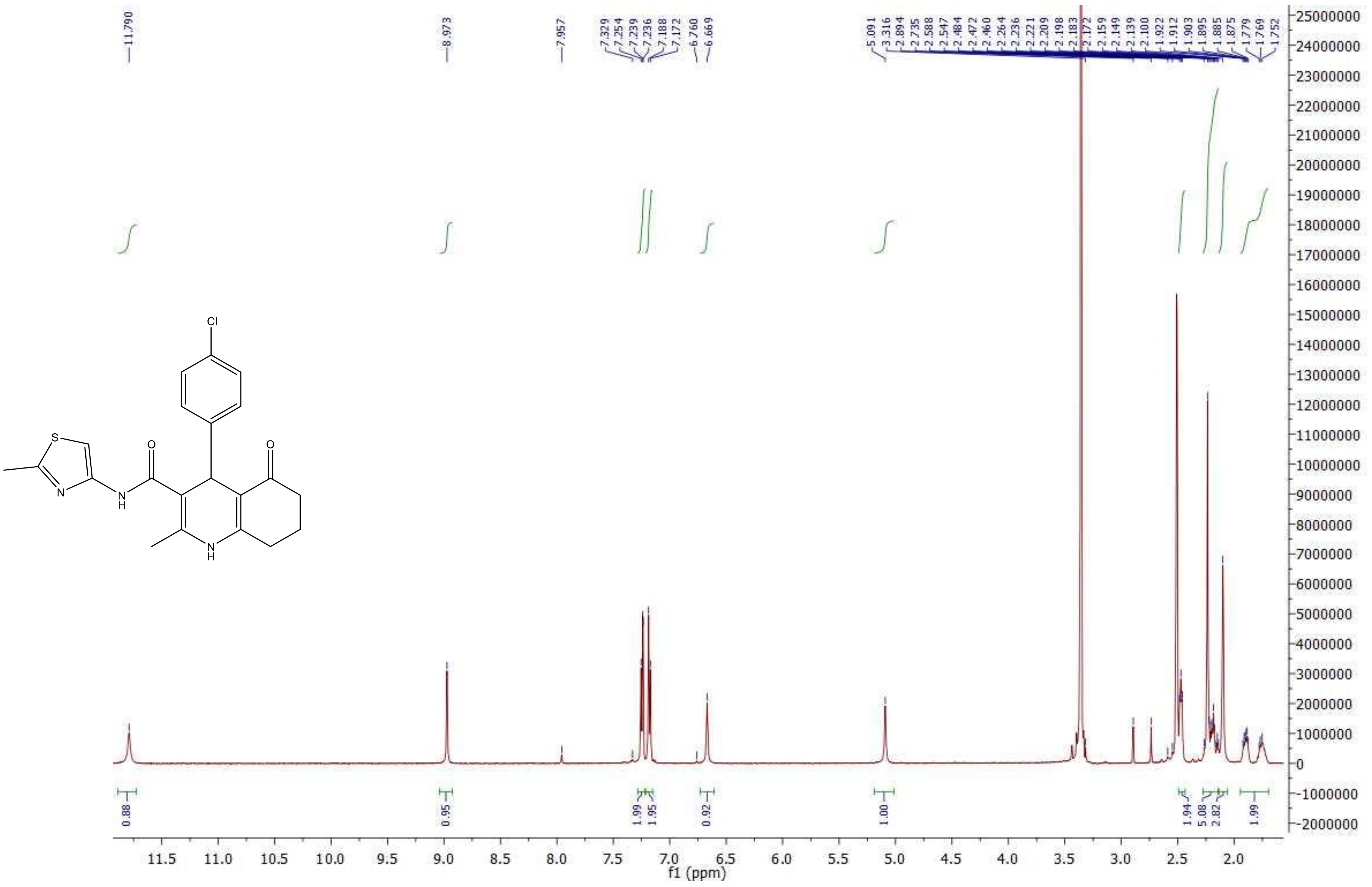

Figure 17s. ^{1}H-NMR spectrum of A5

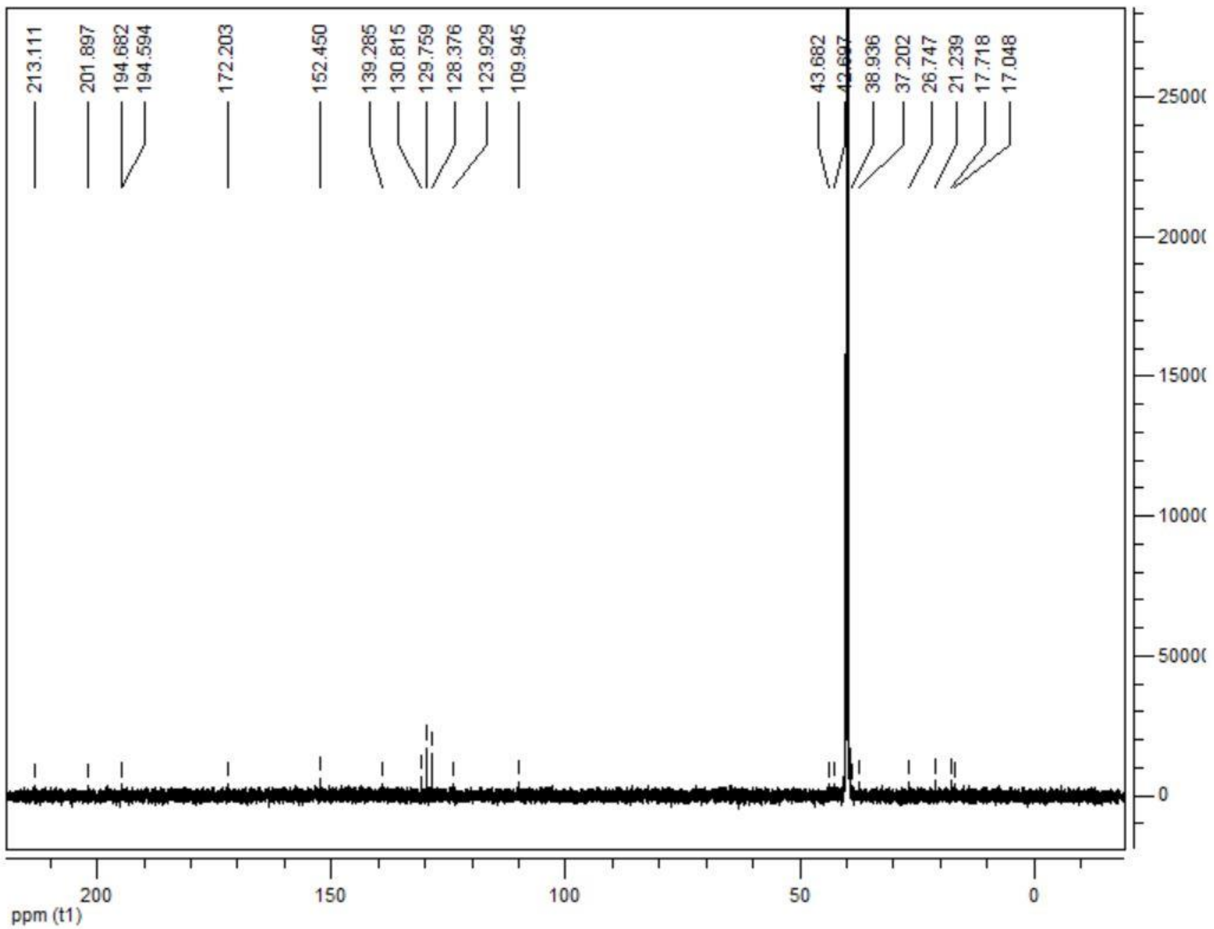

Figure 18s. ^{13}C-NMR spectrum of A5

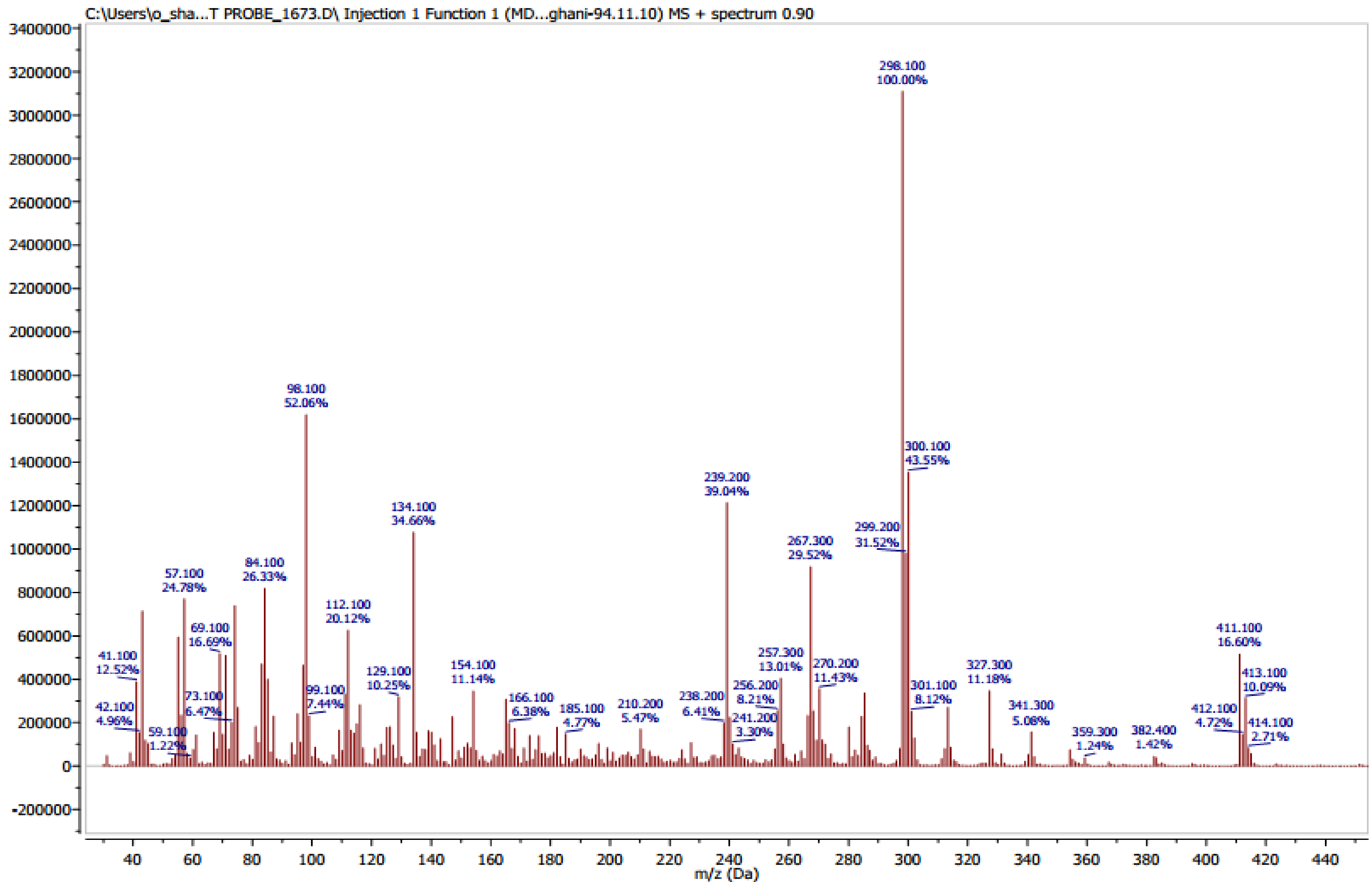

Figure 19s. Mass spectrum of A5

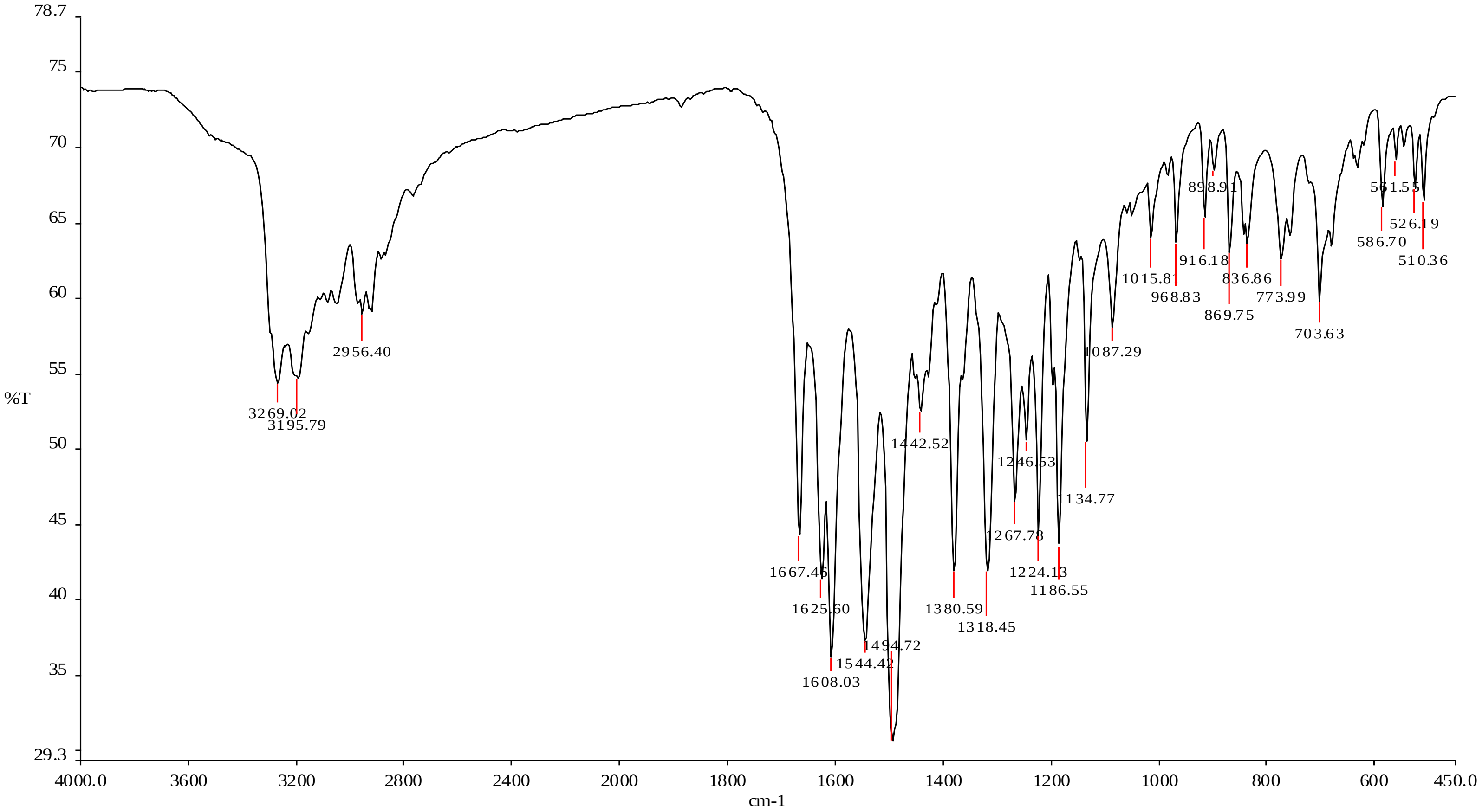

Figure 20s. IR spectrum of A5

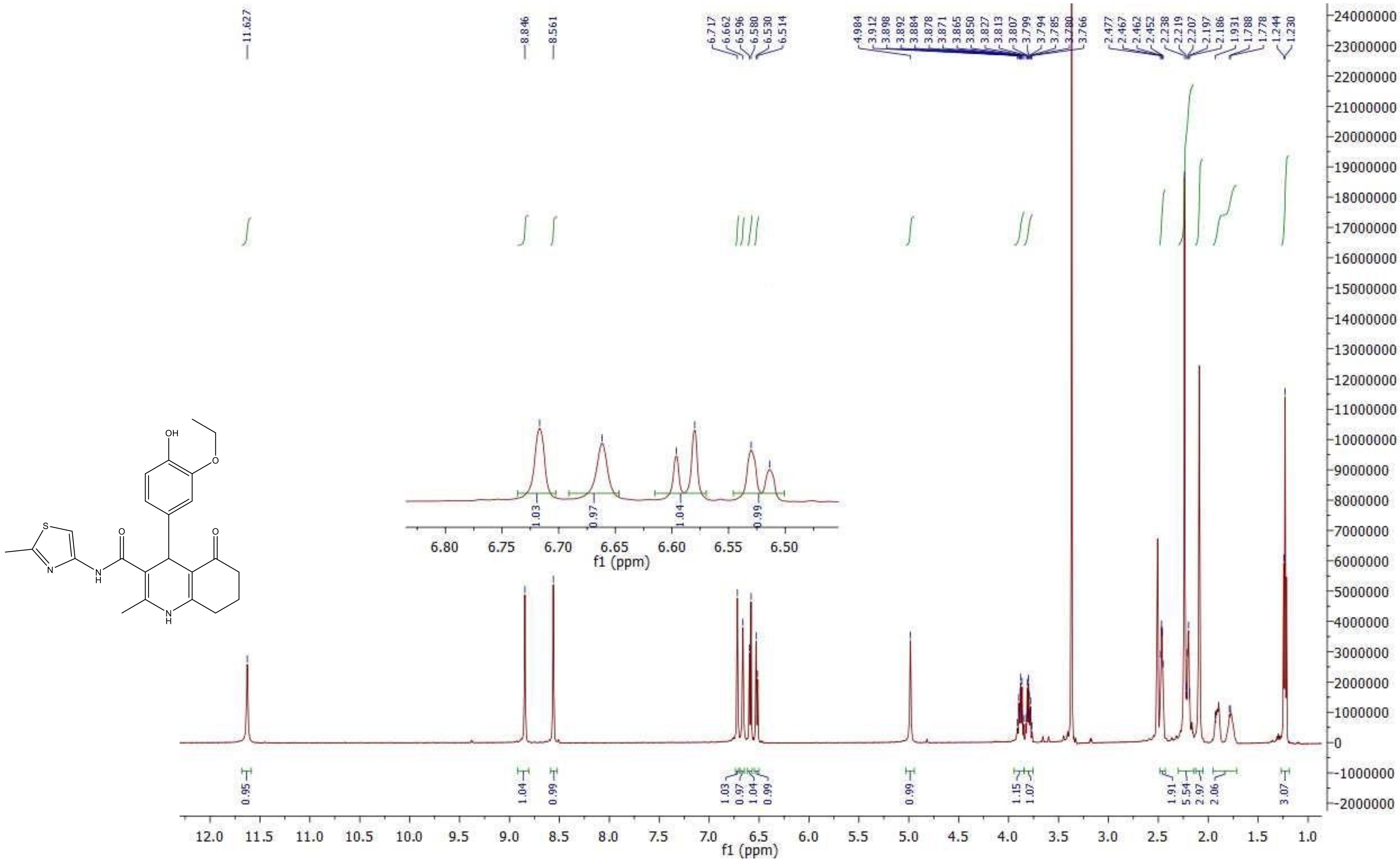

Figure 21s. ^{1}H-NMR spectrum of A6

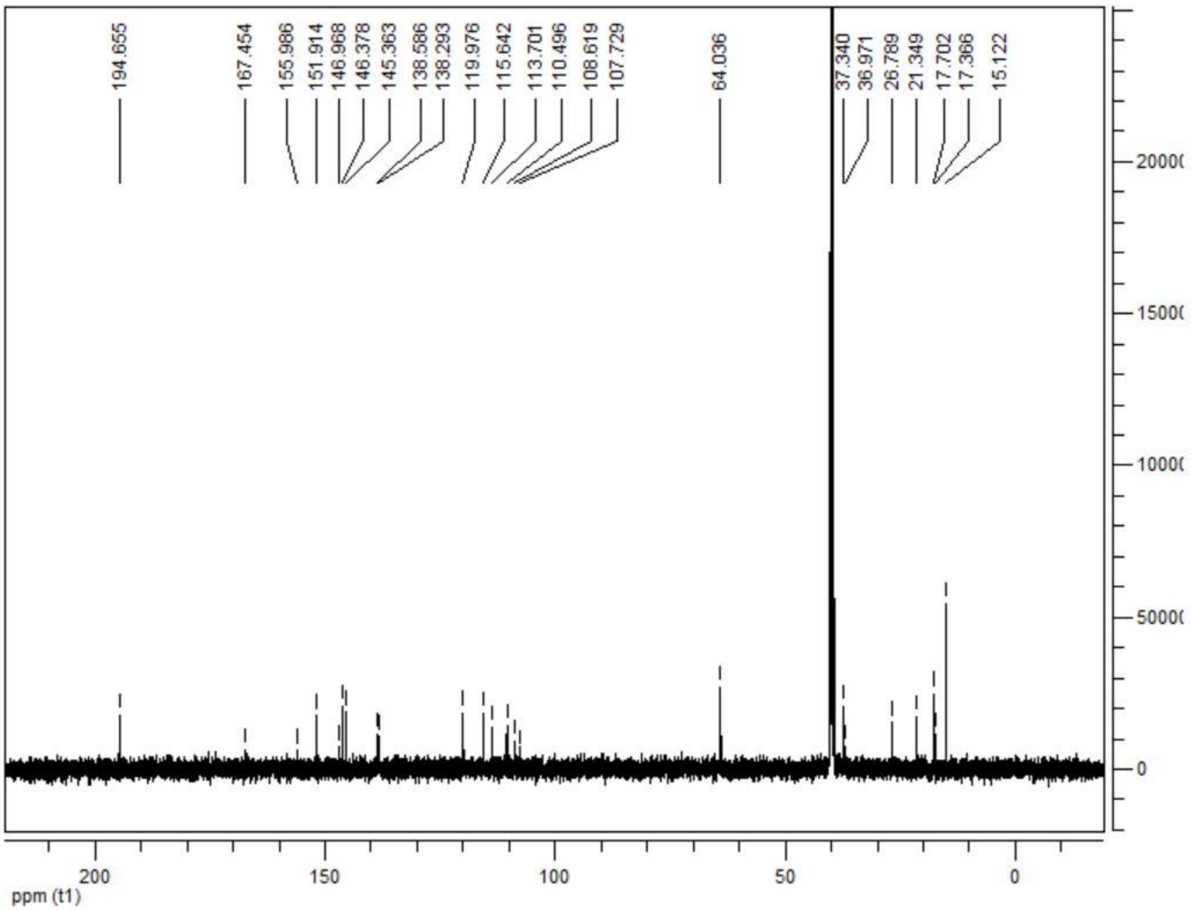

Figure 22s. ^{13}C-NMR spectrum of A6

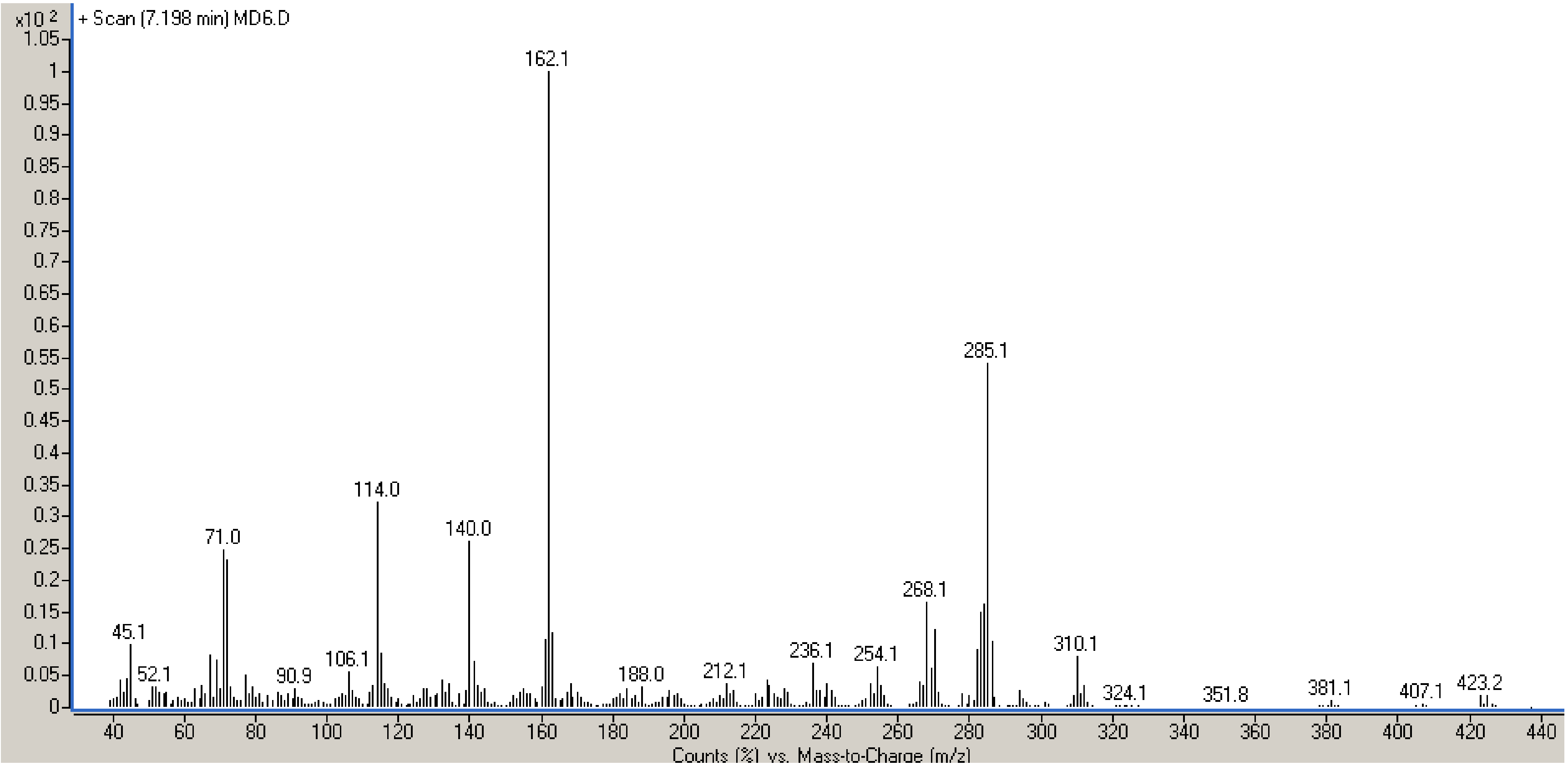

Figure 23s. Mass spectrum of A6

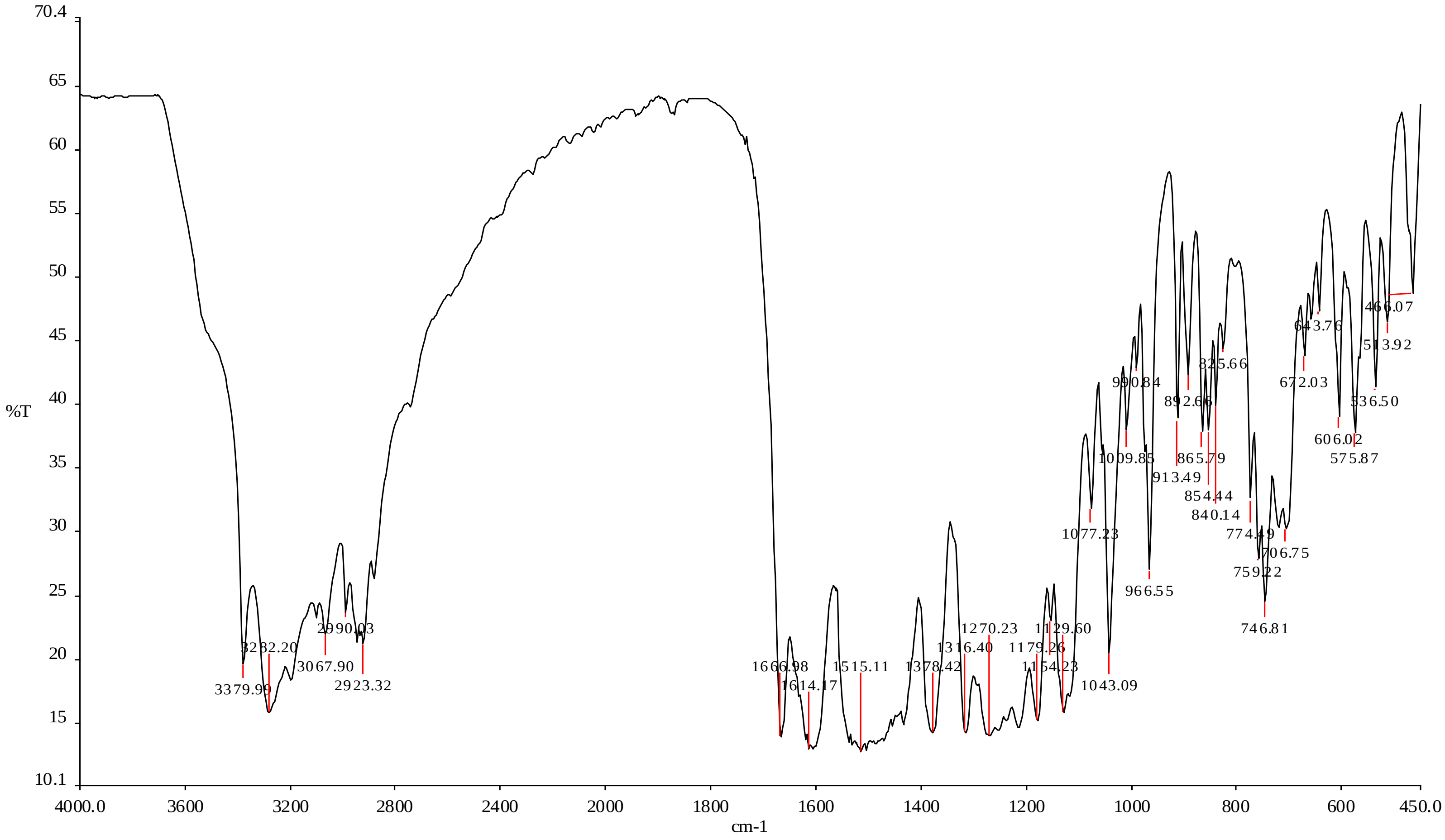

Figure 24s. IR spectrum of A6

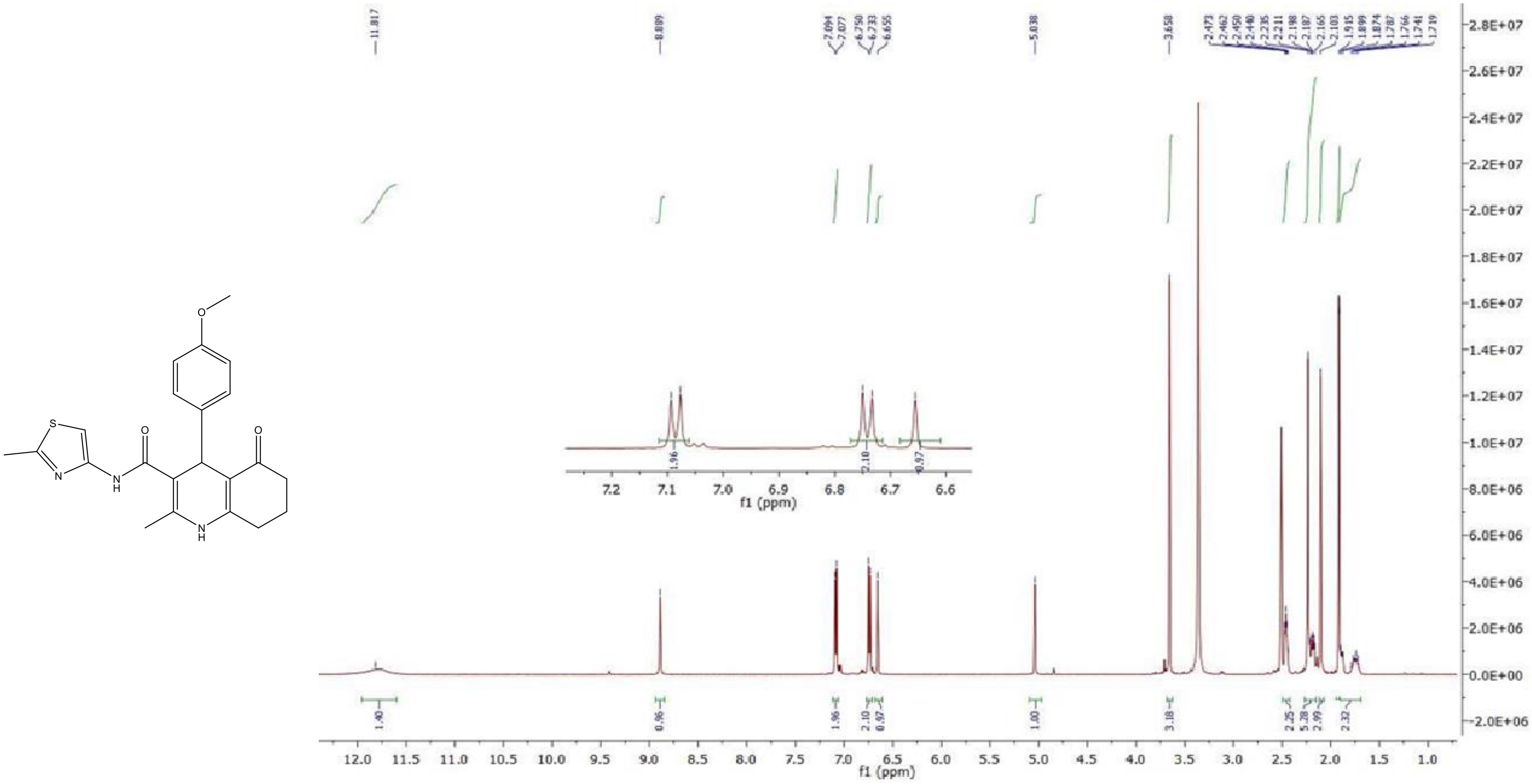

Figure 25s. ^{1}H-NMR spectrum of A7

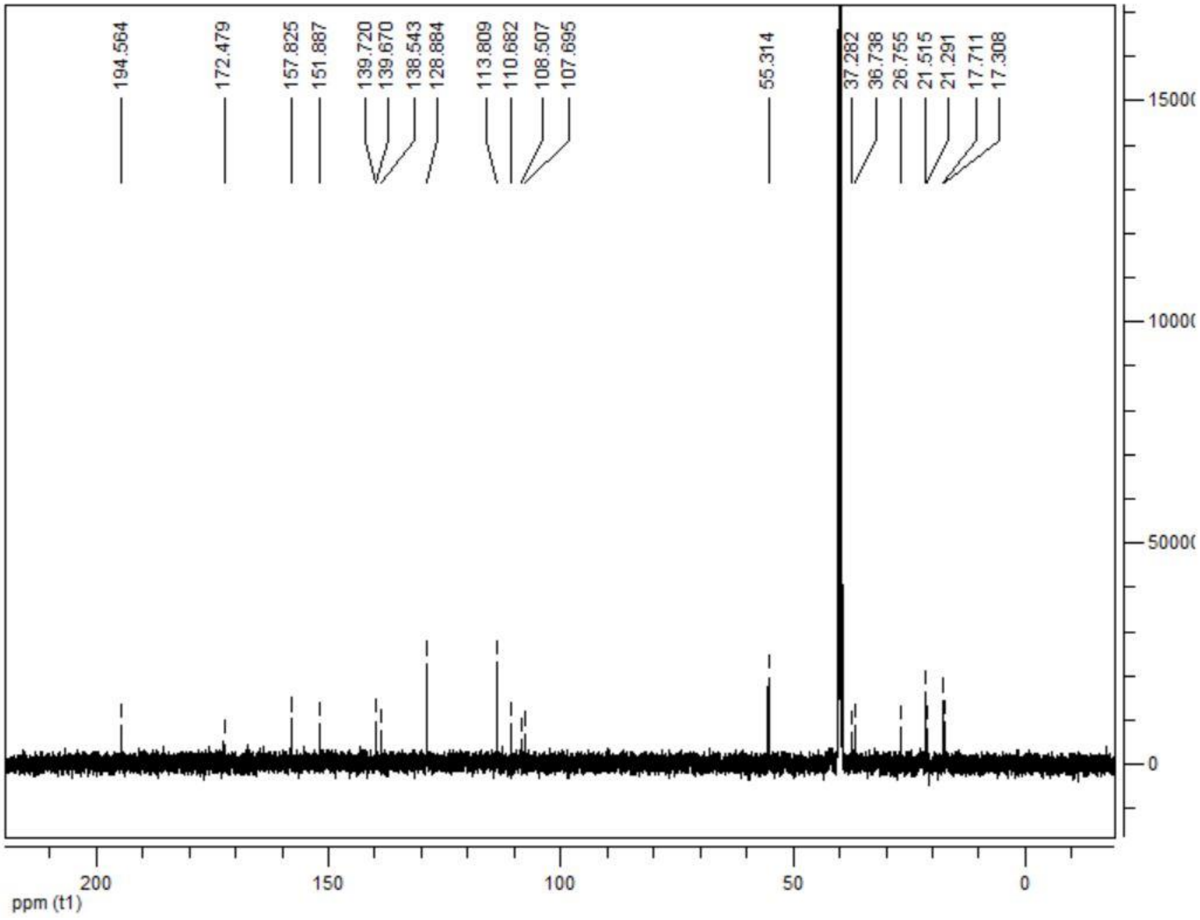

Figure 26s. ^{13}C-NMR spectrum of A7

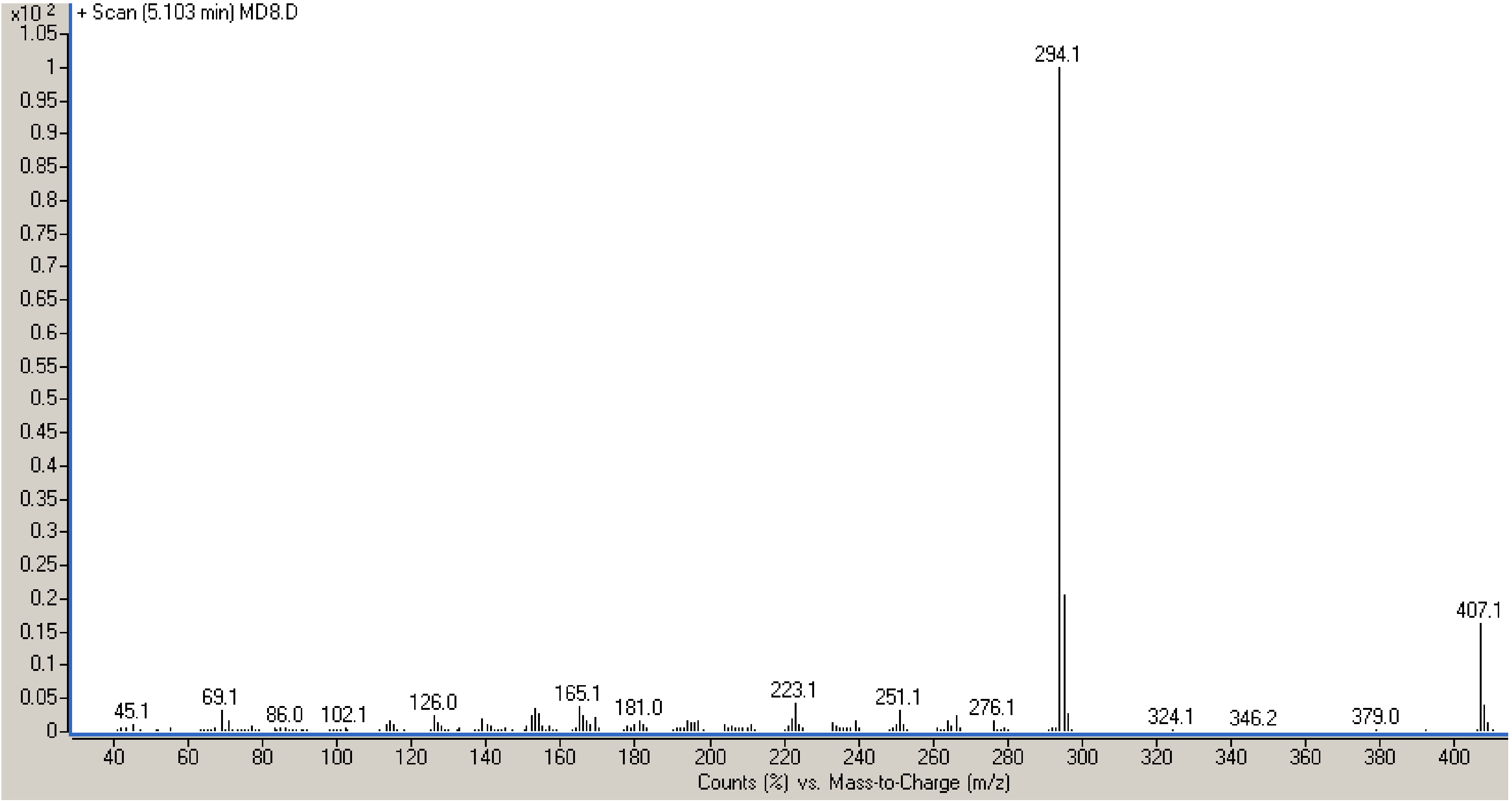

Figure 27s. Mass spectrum of A7

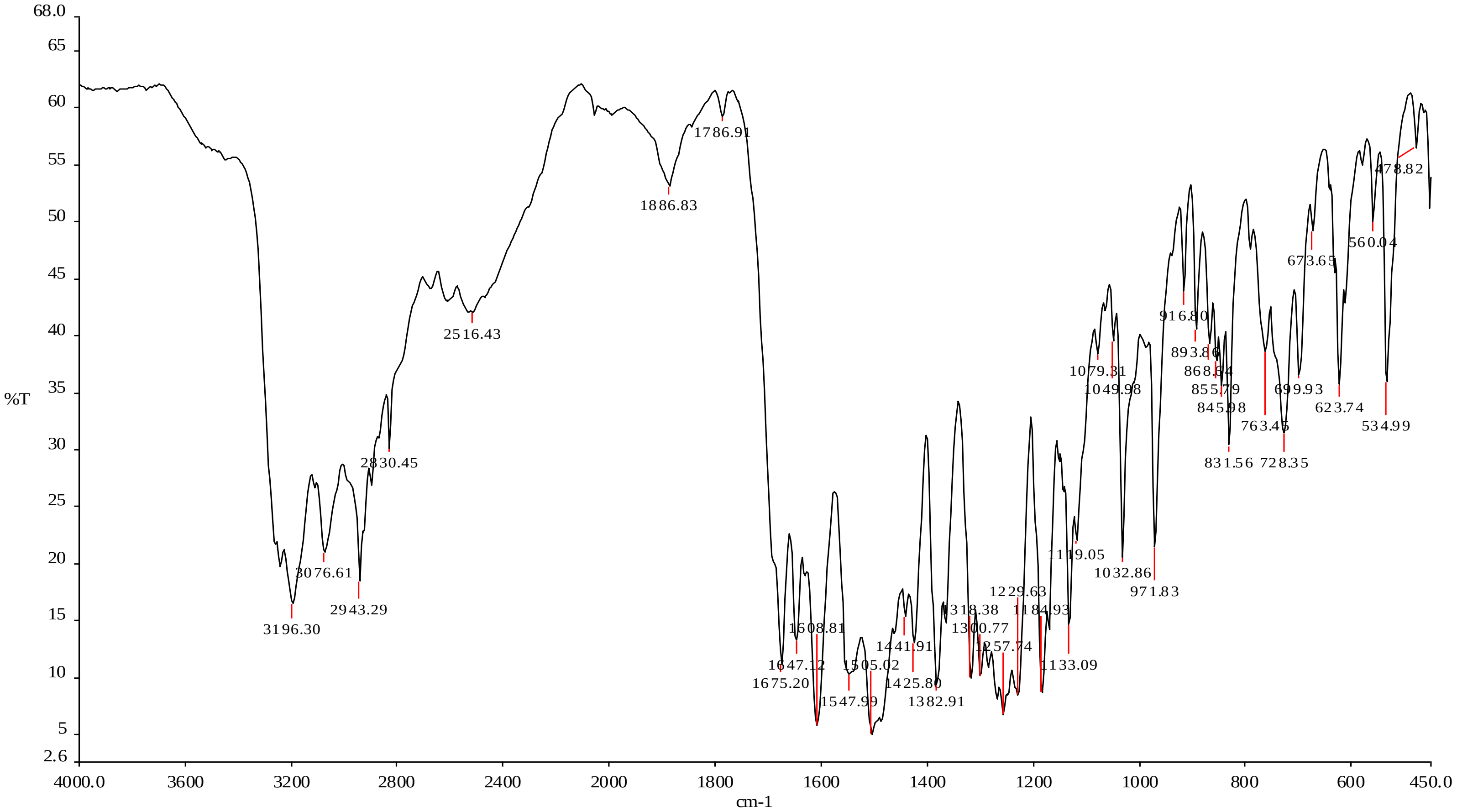

Figure 28s. IR spectrum of A7

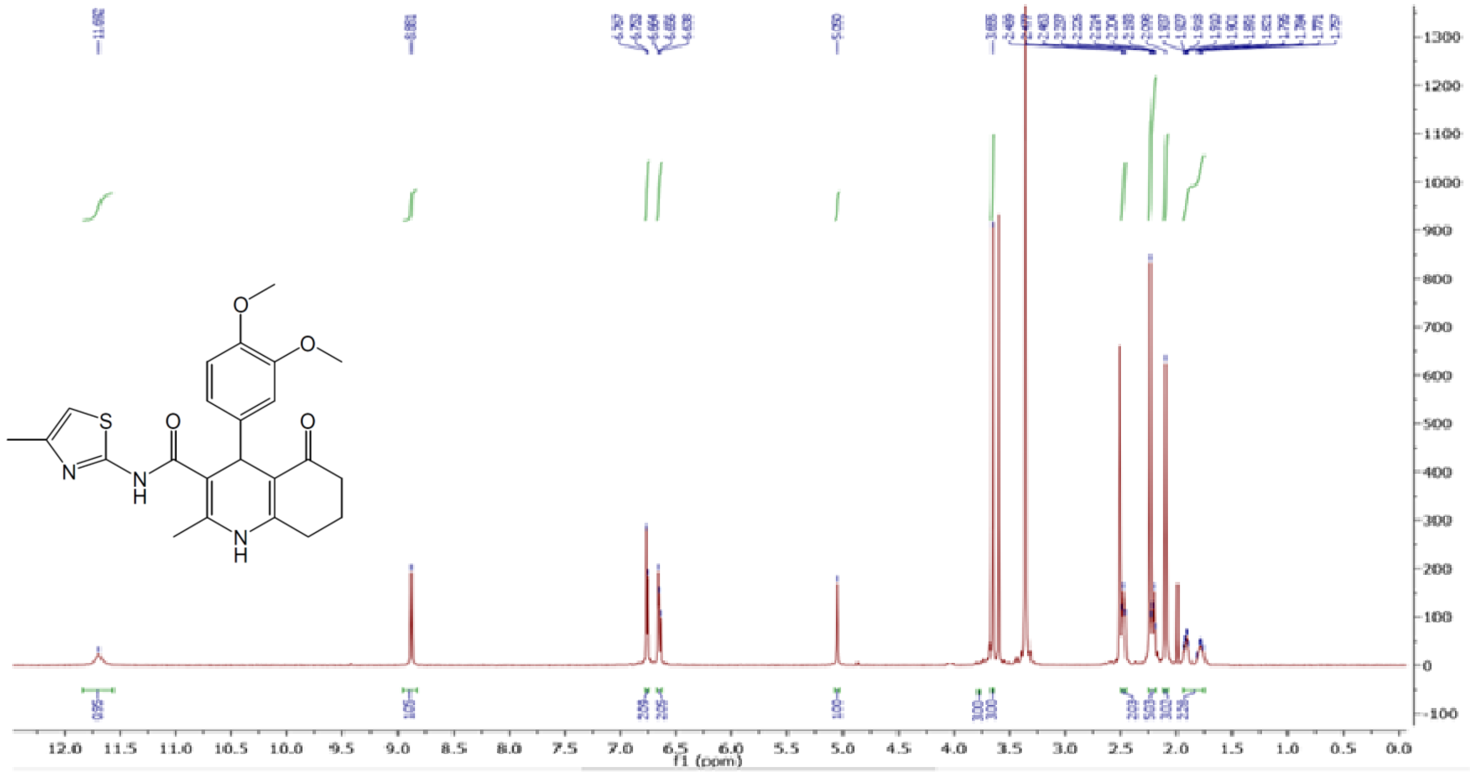

Figure 29s. ^{1}H NMR spectrum of A8

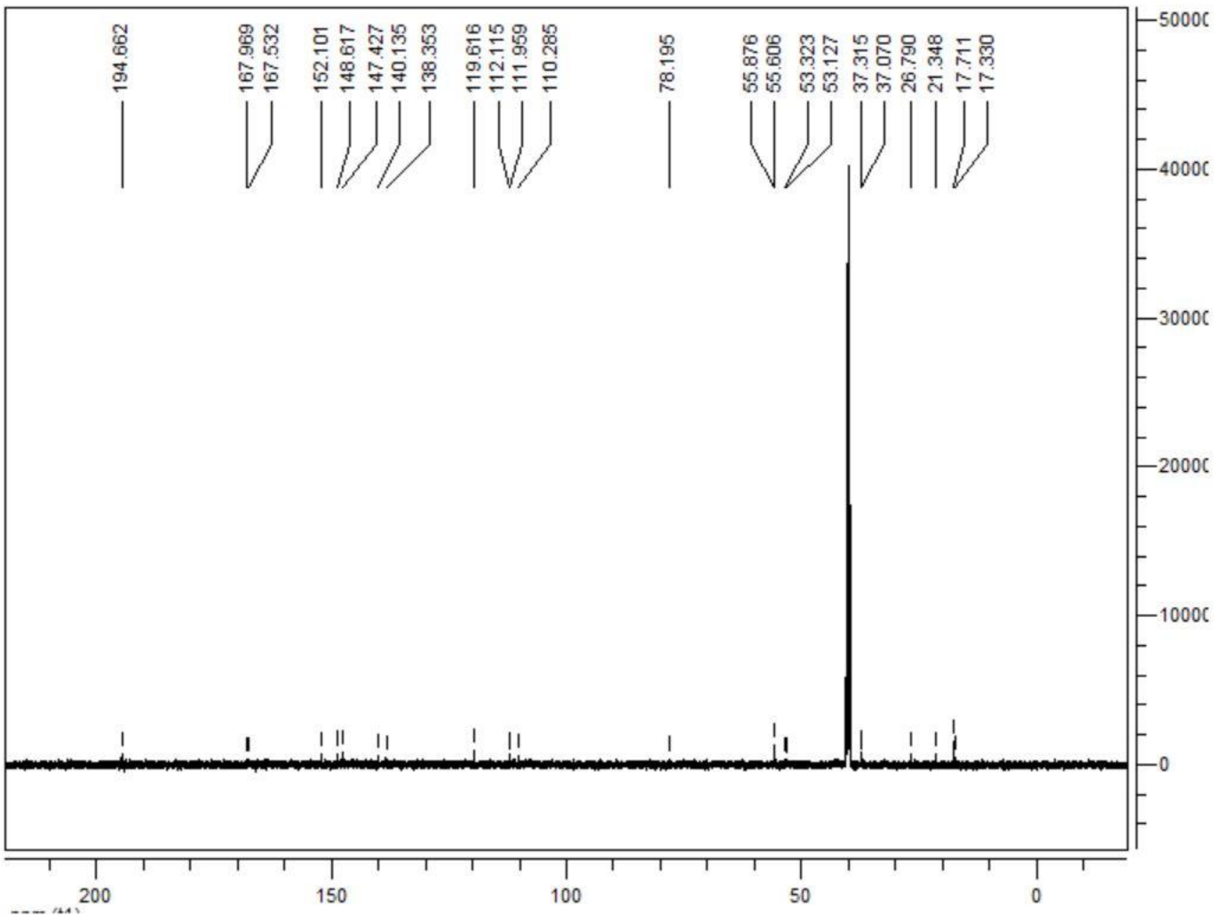

Figure 30s. ^{13}C NMR spectrum of A8

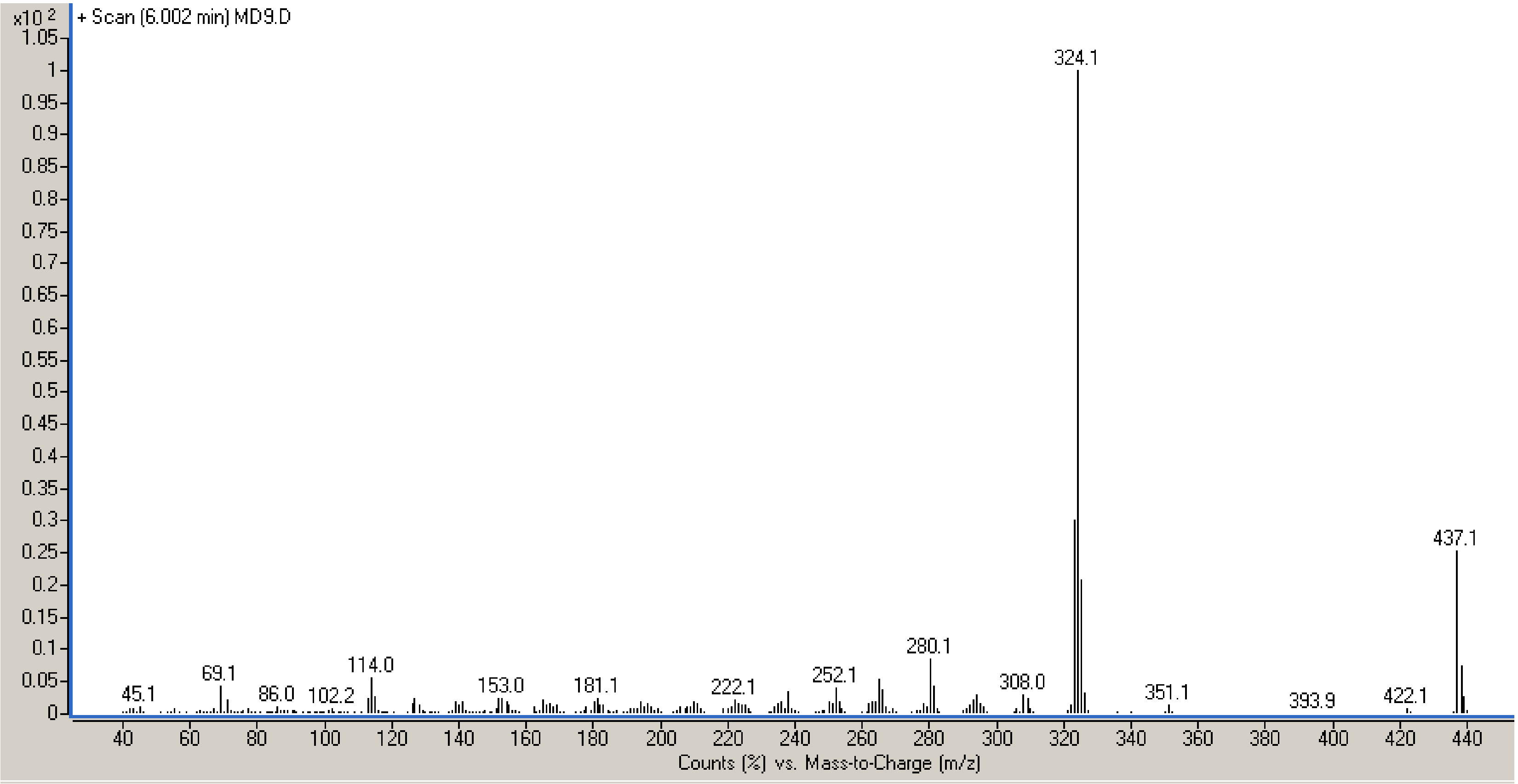

Figure 31s. Mass spectrum of A8

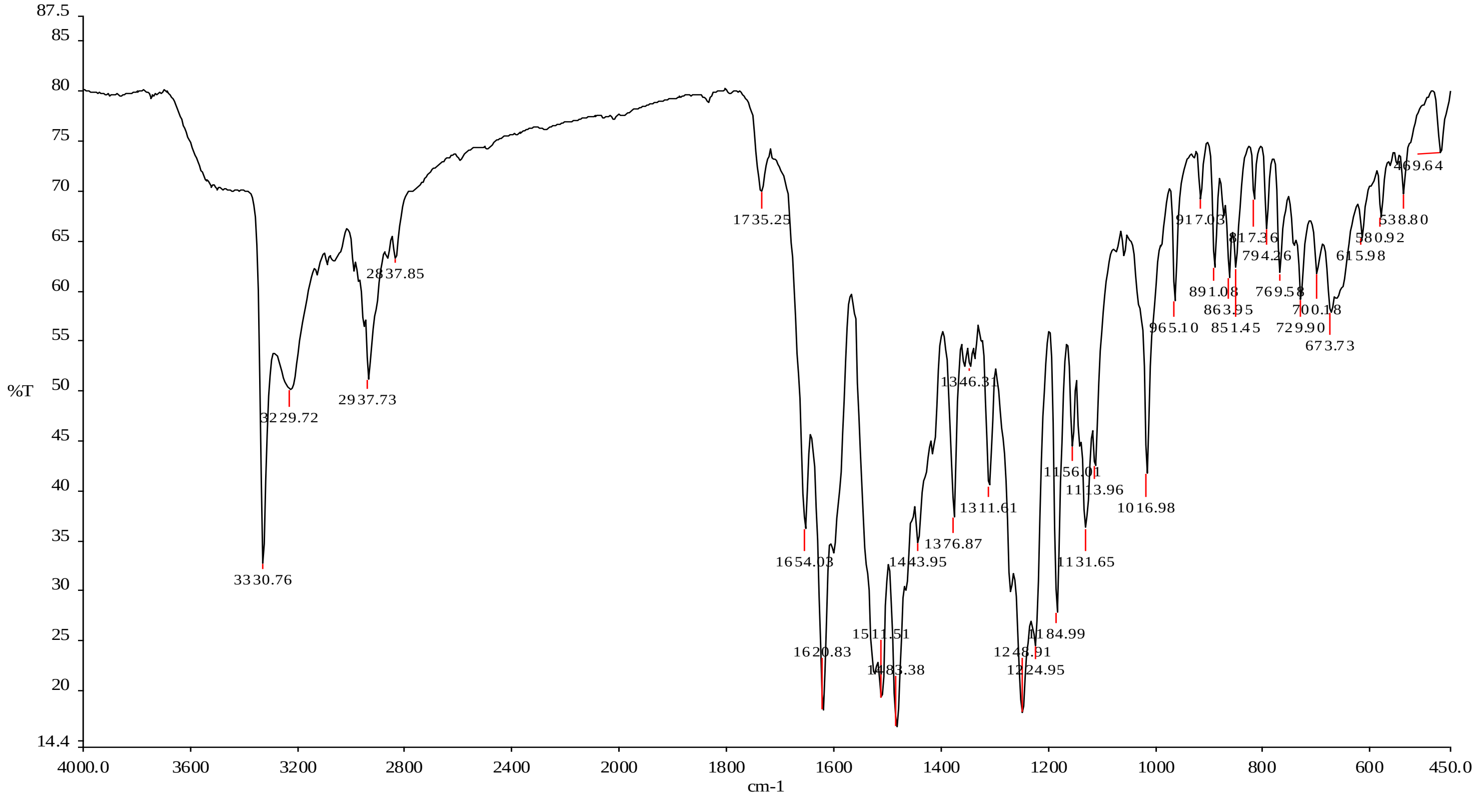

Figure 32s. IR spectrum of A8

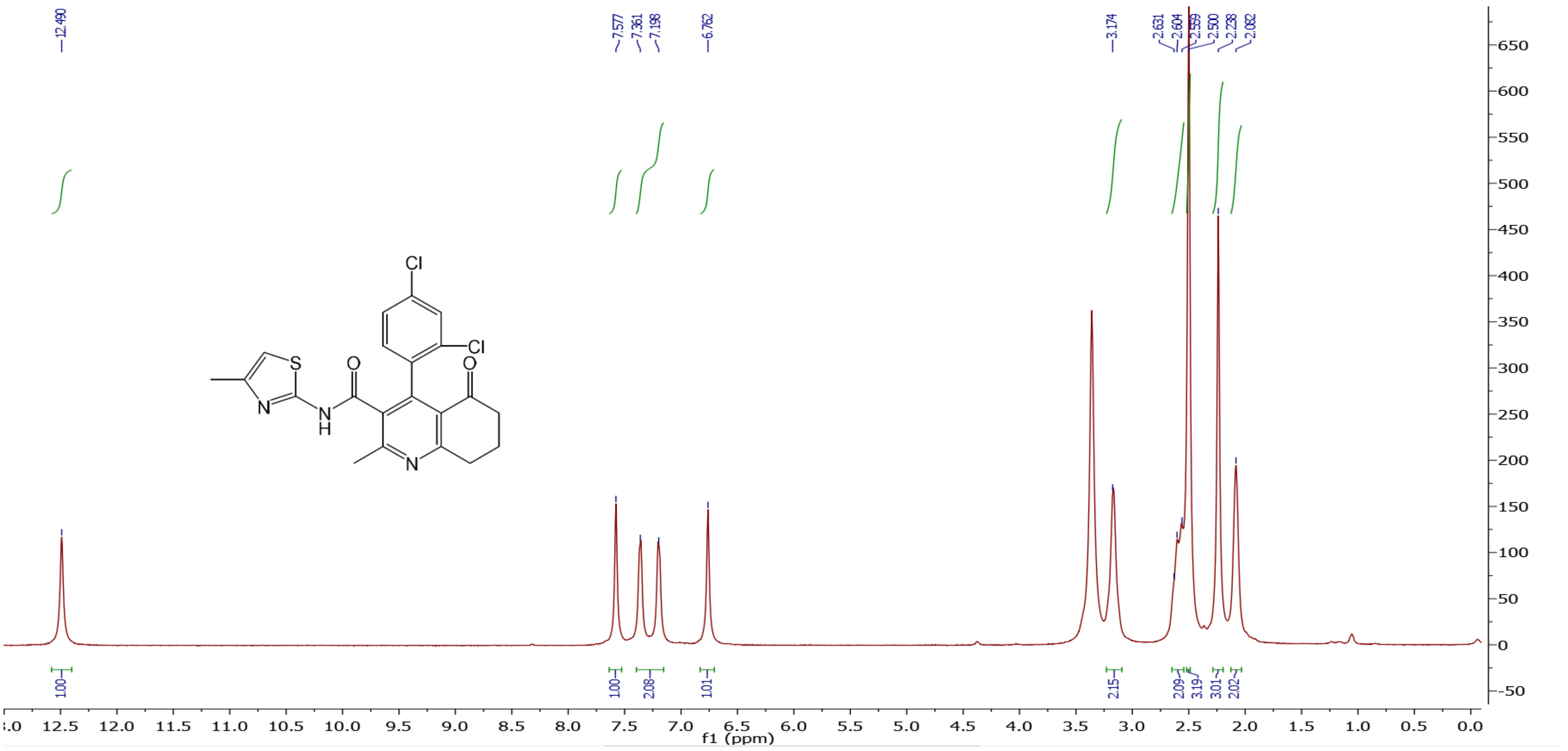

Figure 33s. ^{1}H NMR spectrum of B1

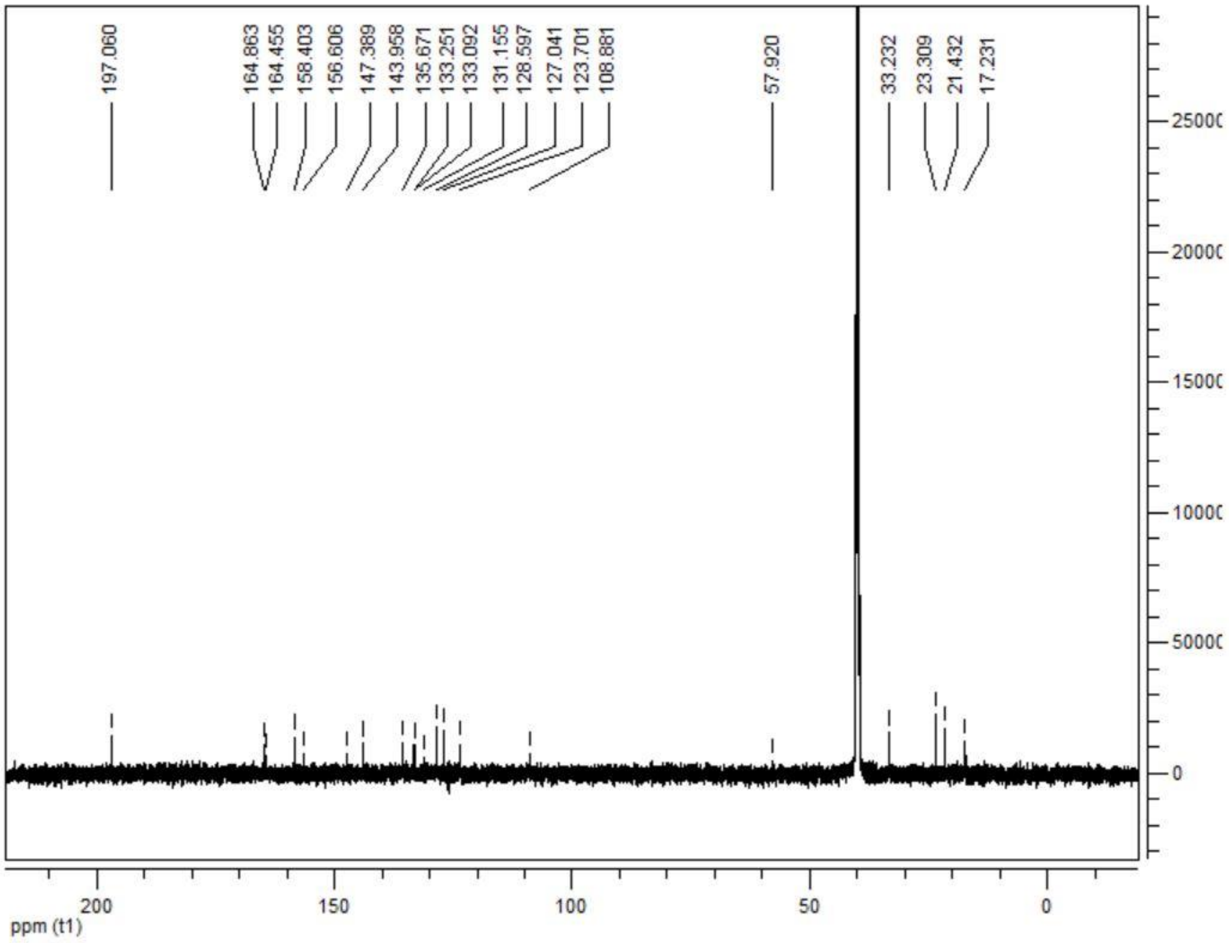

Figure 34s. ^{13}C NMR spectrum of B1

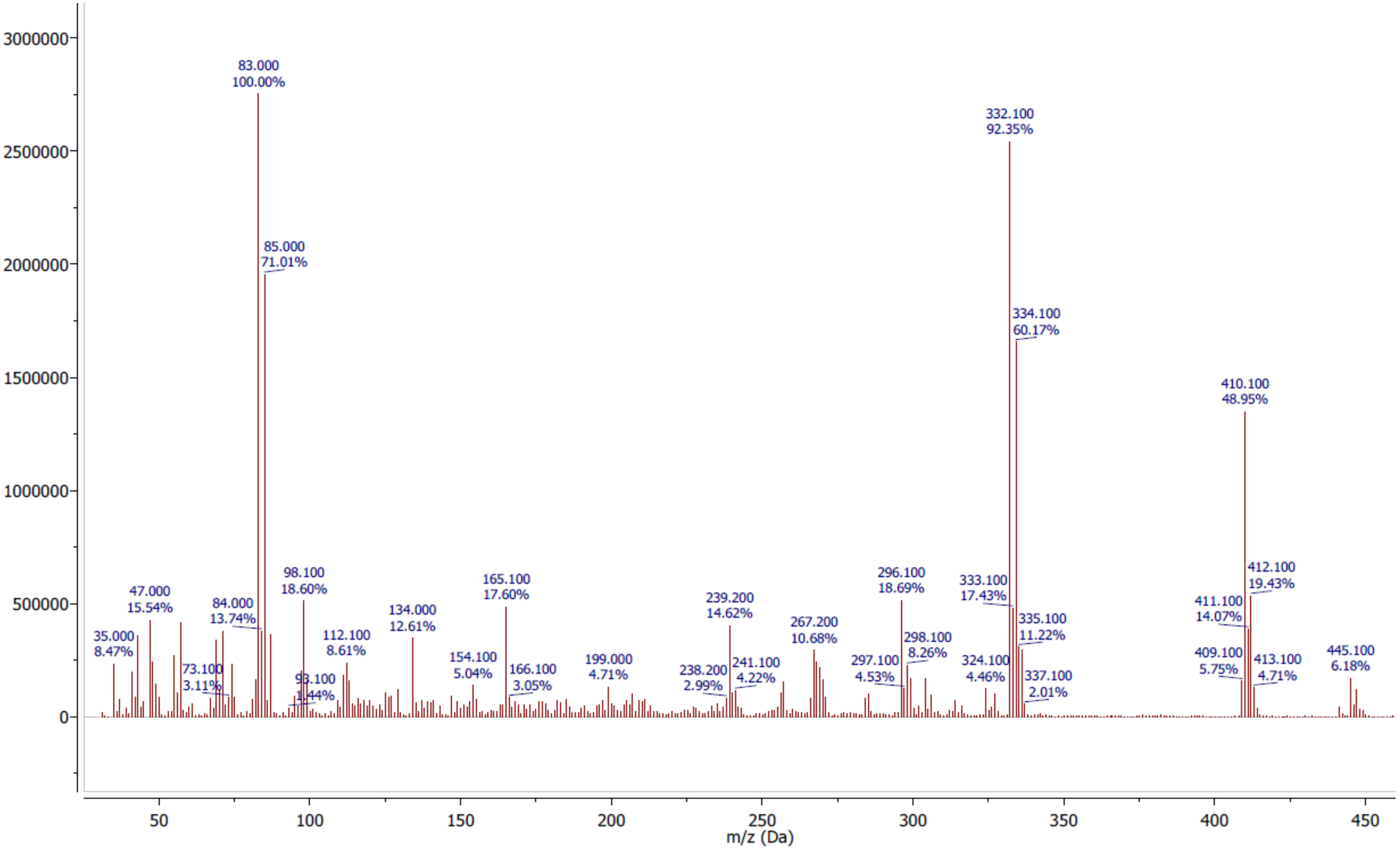

Figure 35s. Mass spectrum of B1

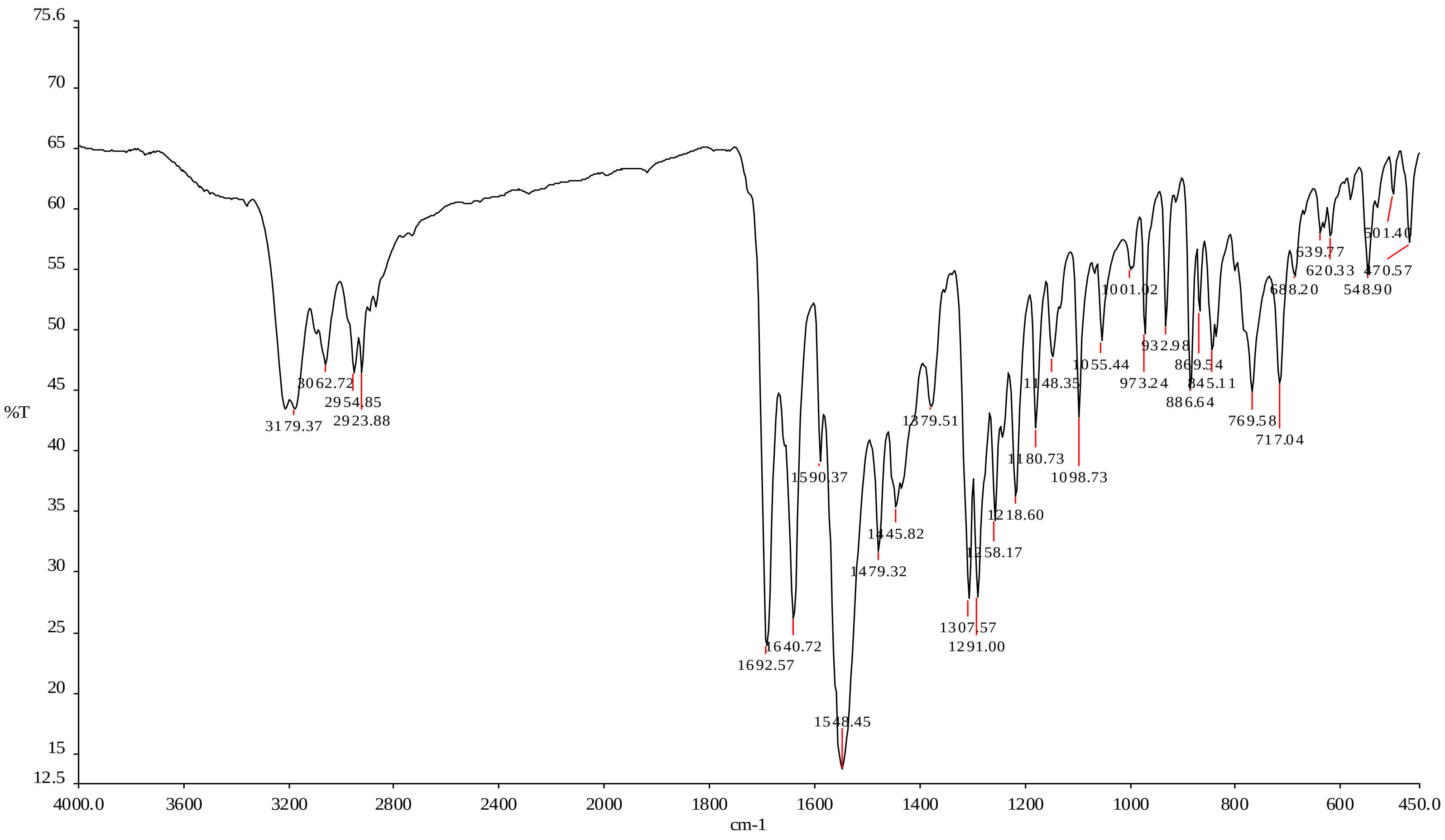

Figure 36s. IR spectrum of B1

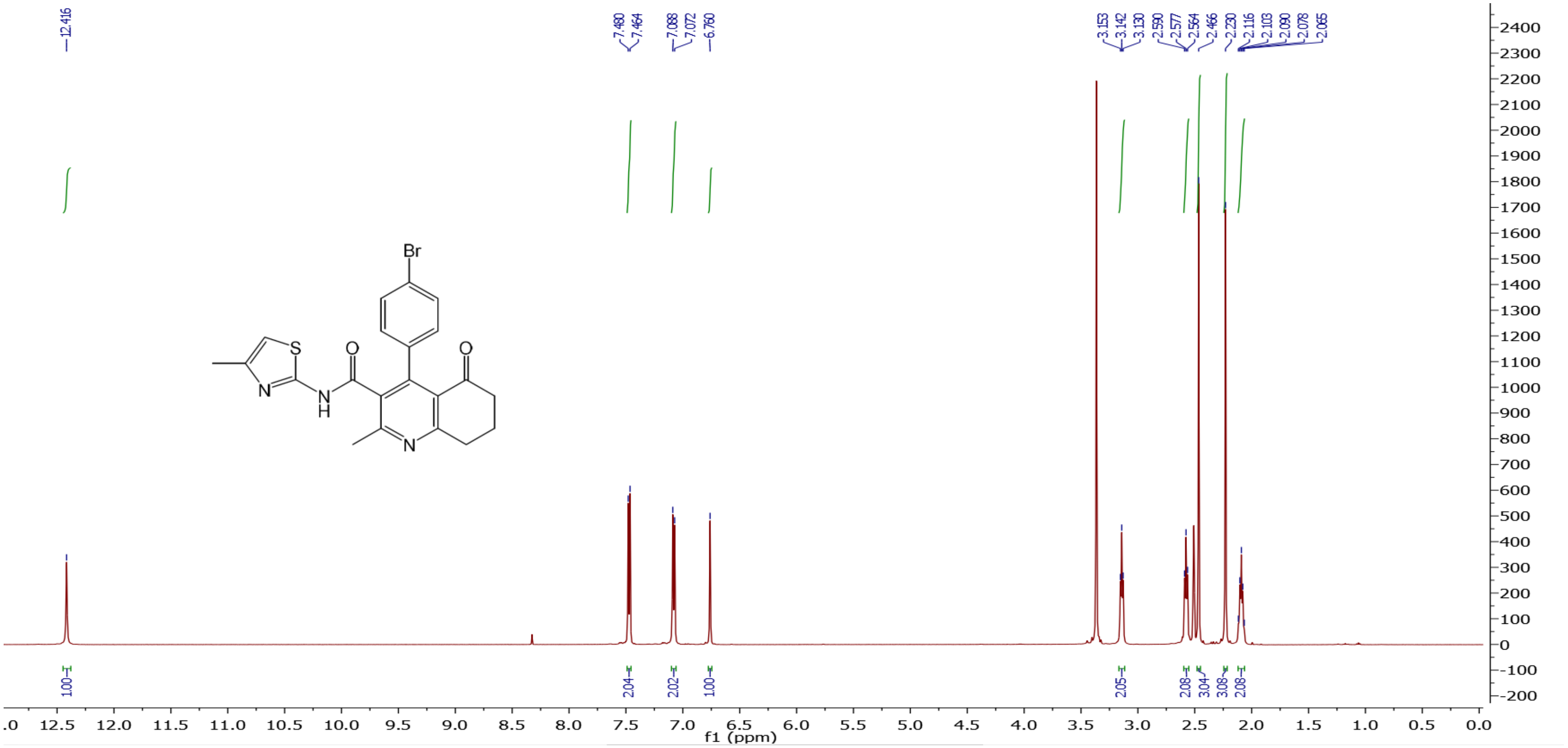

Figure 37s. ^{1}H NMR spectrum of B2

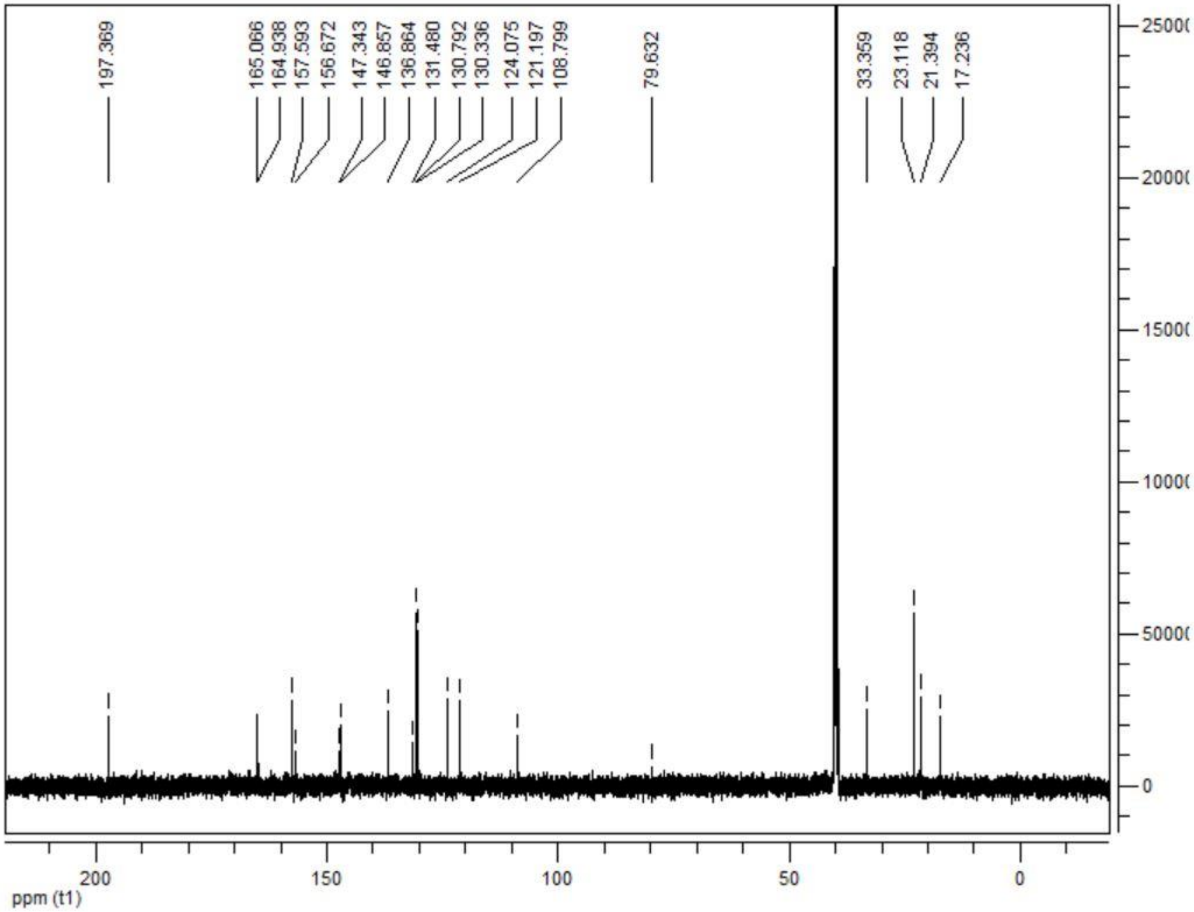

Figure 38s. ^{13}C NMR spectrum of B2

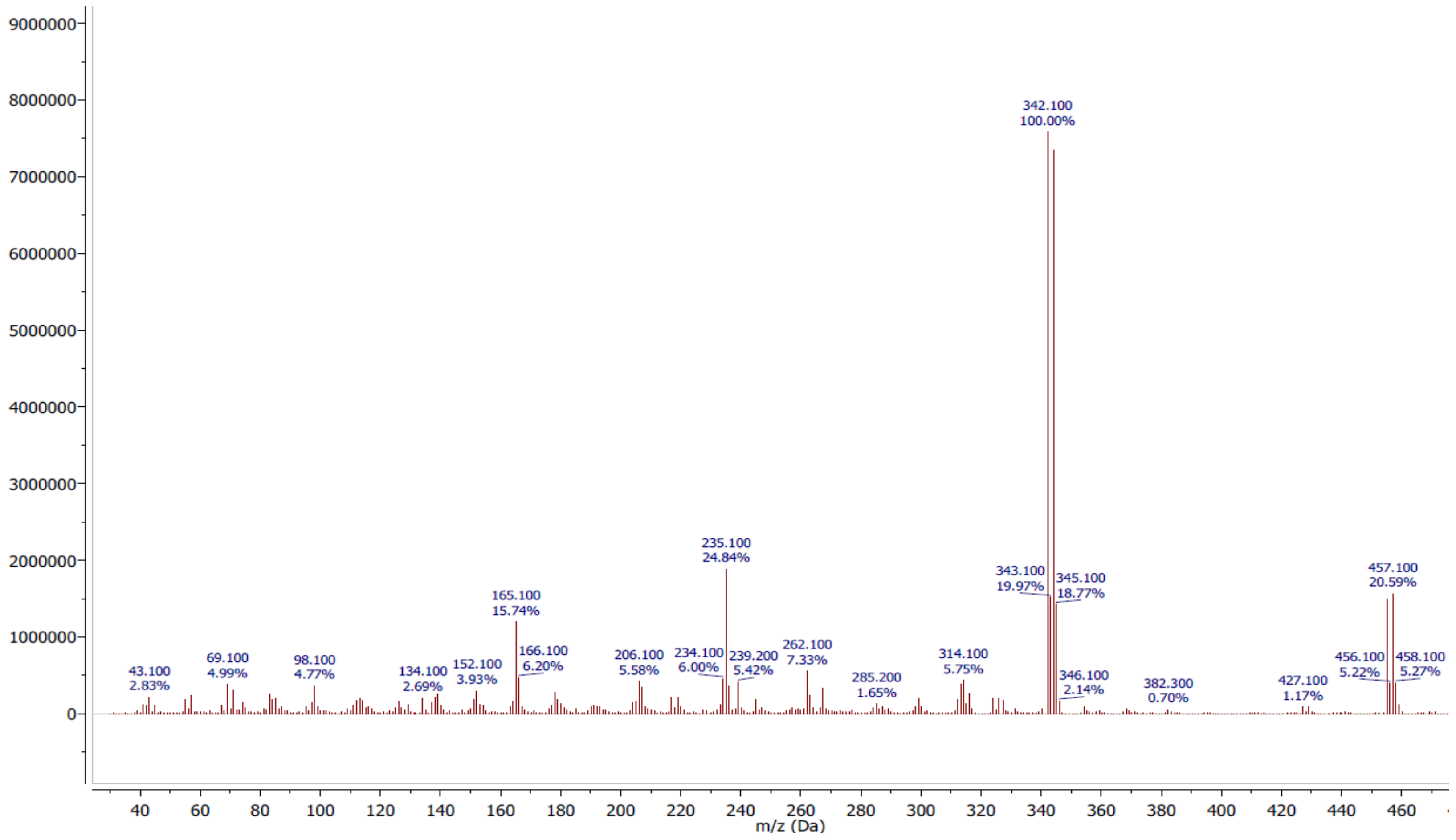

Figure 39s. Mass spectrum of B2

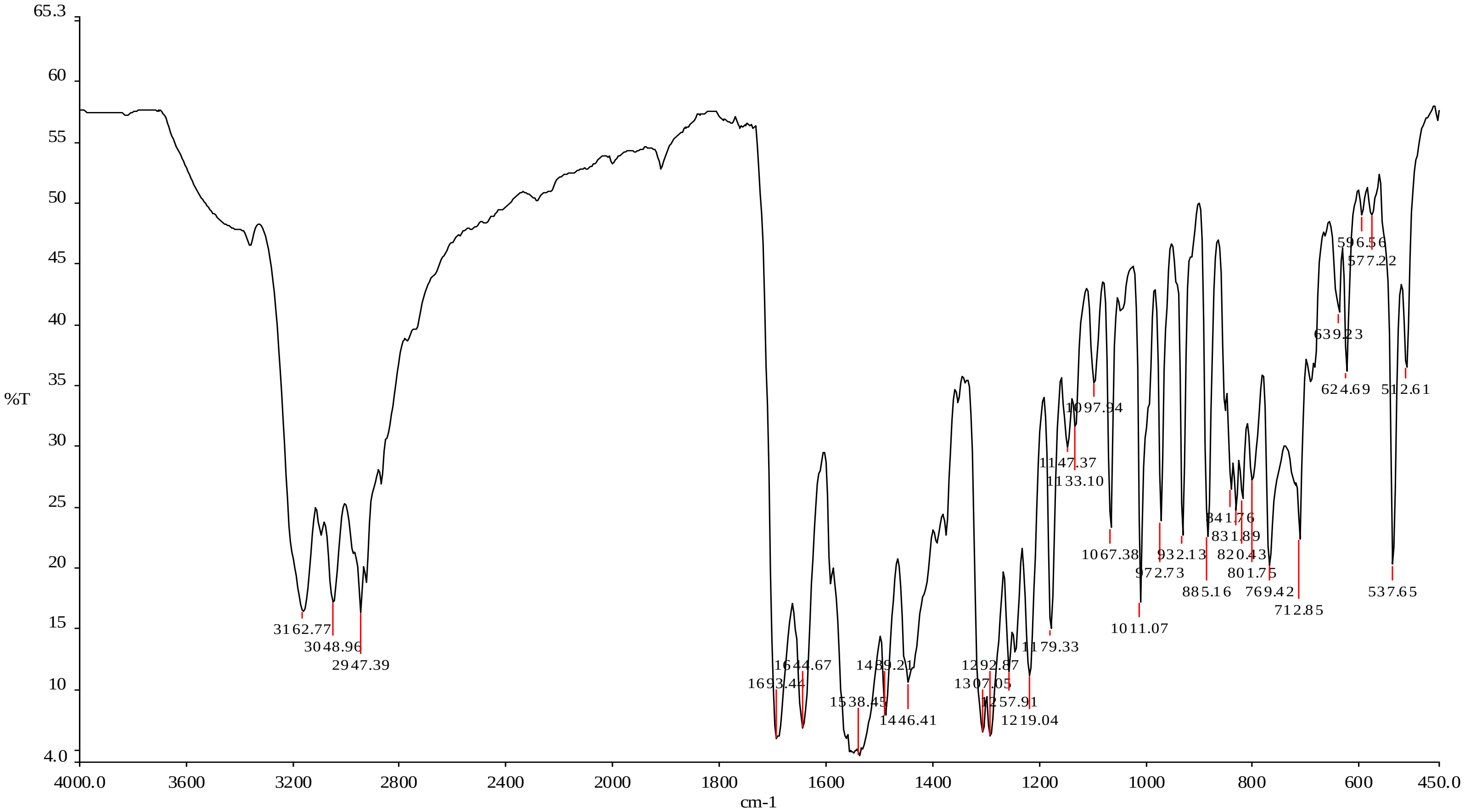

Figure 40s. IR spectrum of B2

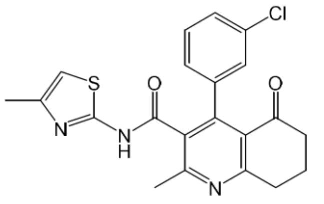

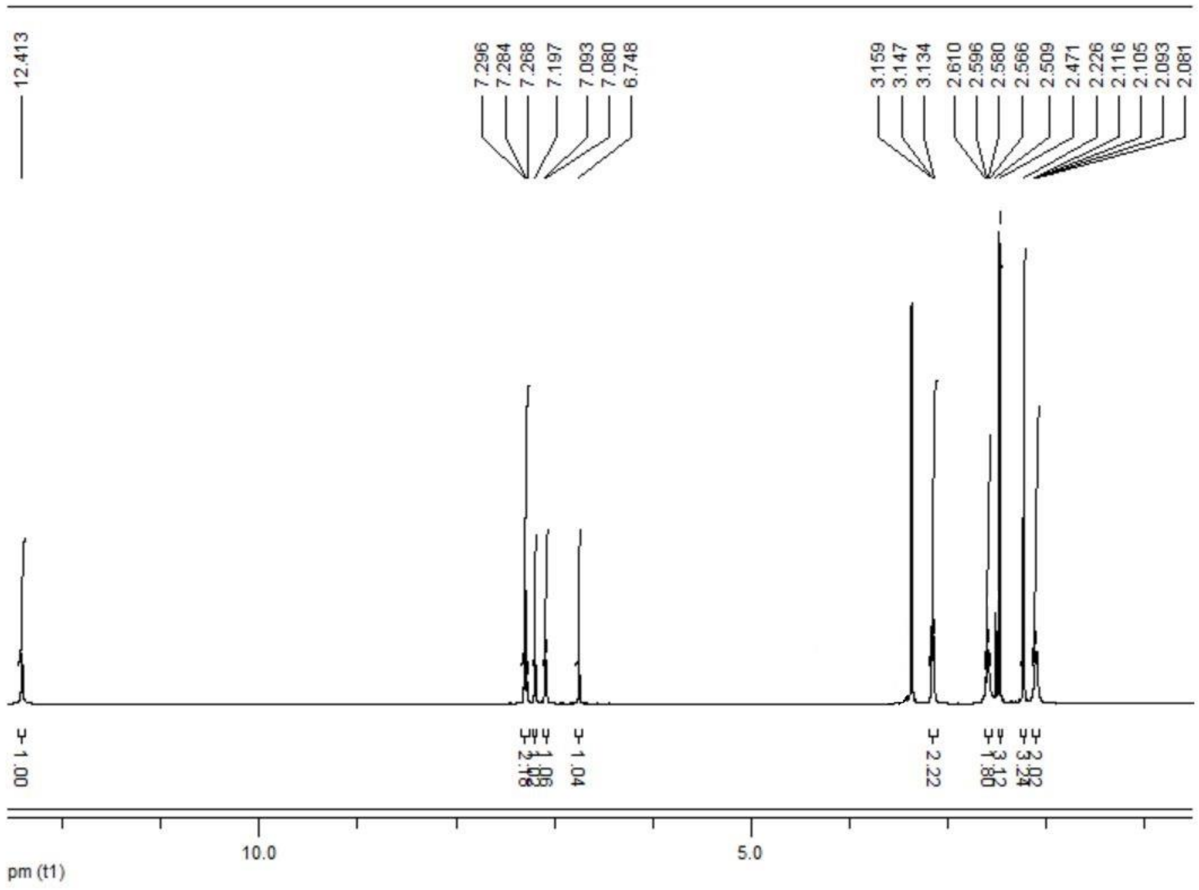

Figure 41s. ¹H NMR spectrum of B3

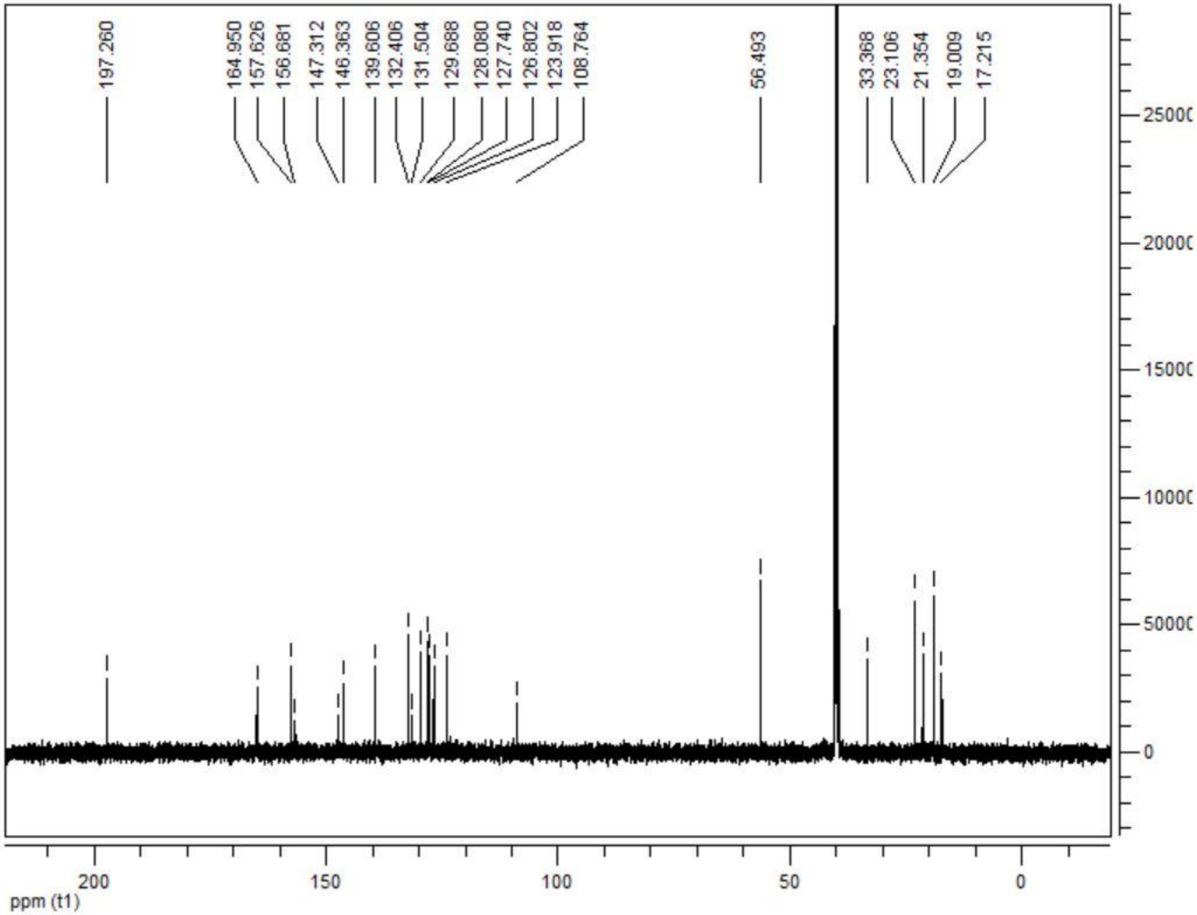

Figure 42s. ^{13}C NMR spectrum of B3

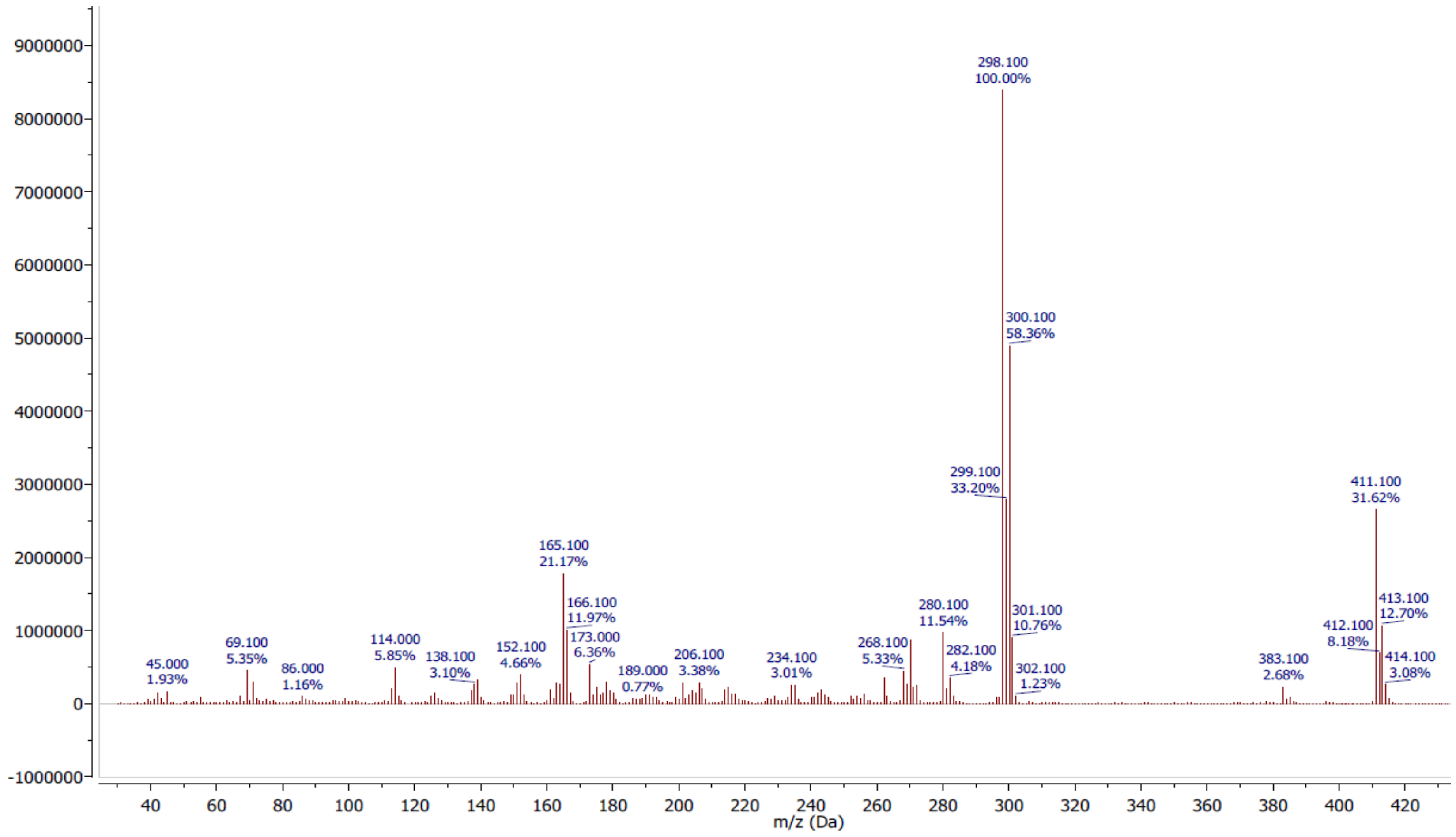

Figure 43s. Mass spectrum of B3

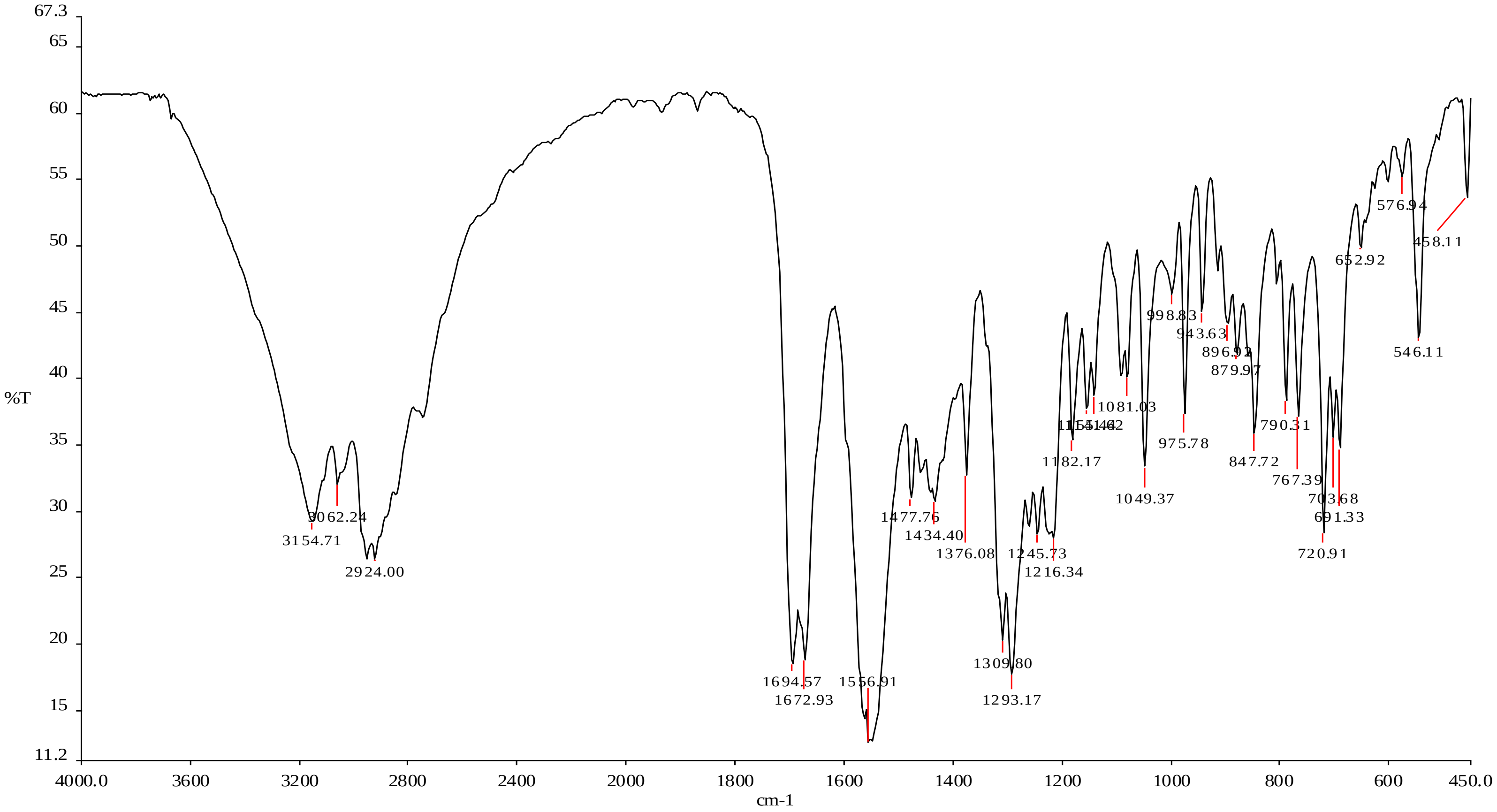

Figure 44s. IR spectrum of B3

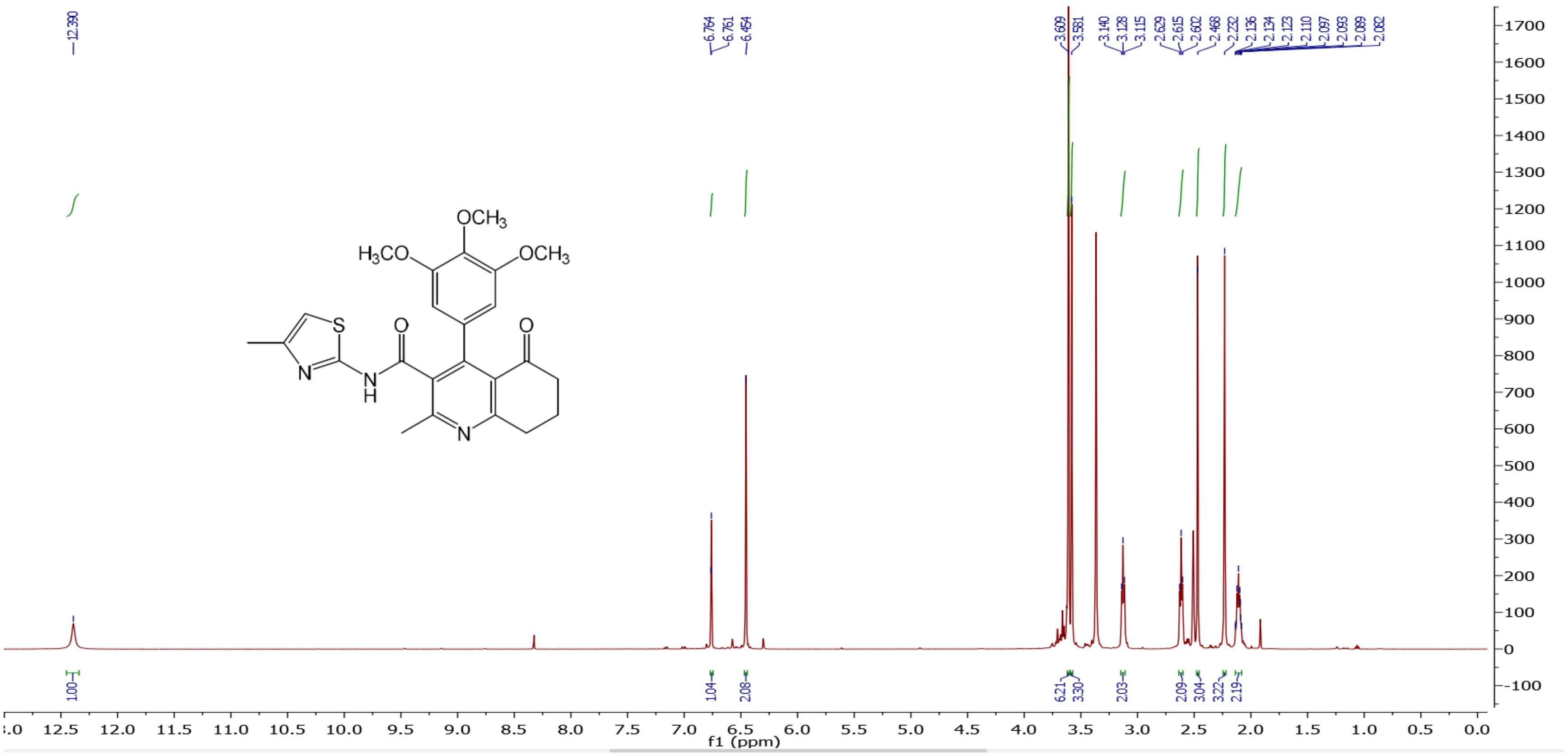

Figure 45s. ^{1}H NMR spectrum of B4

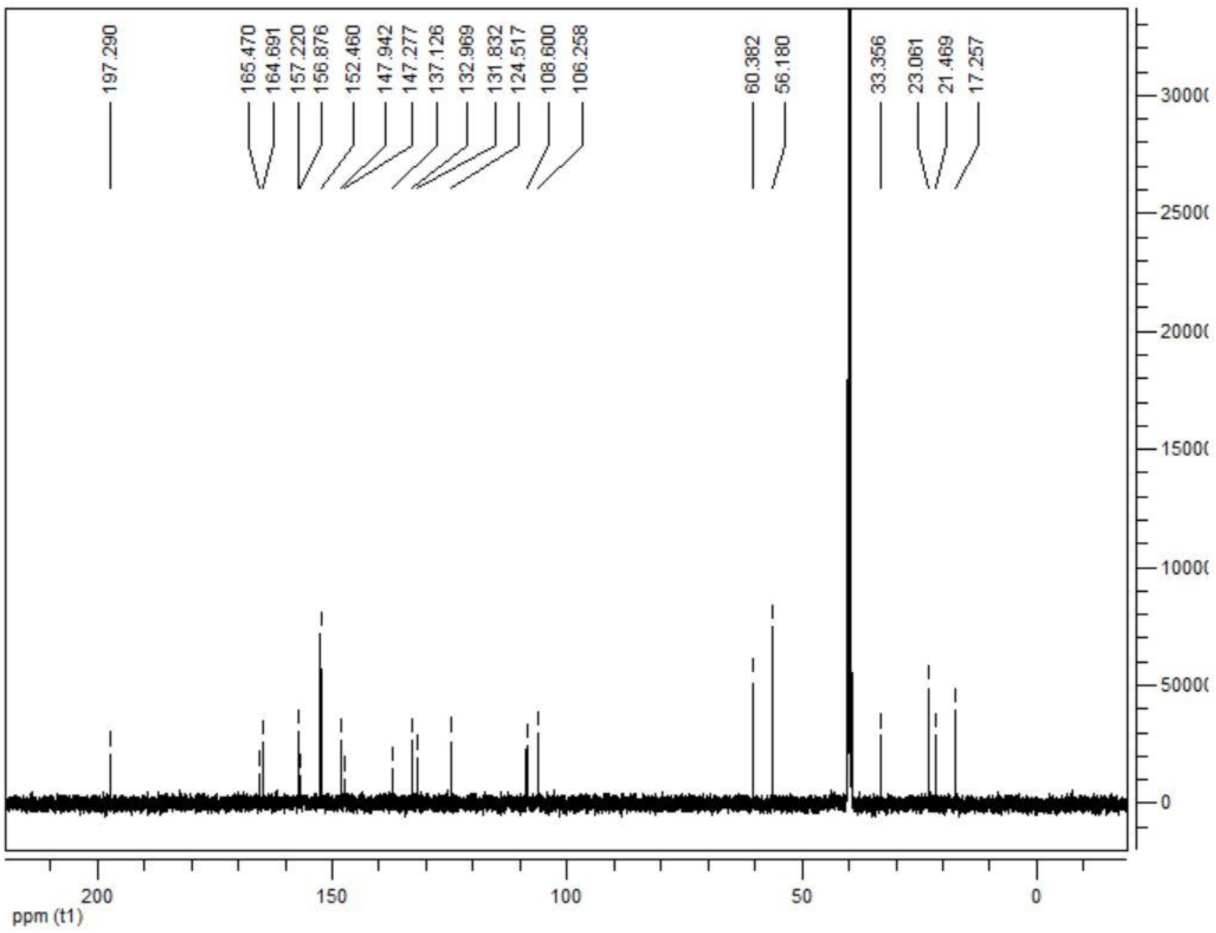

Figure 46s. ^{13}C NMR spectrum of B4

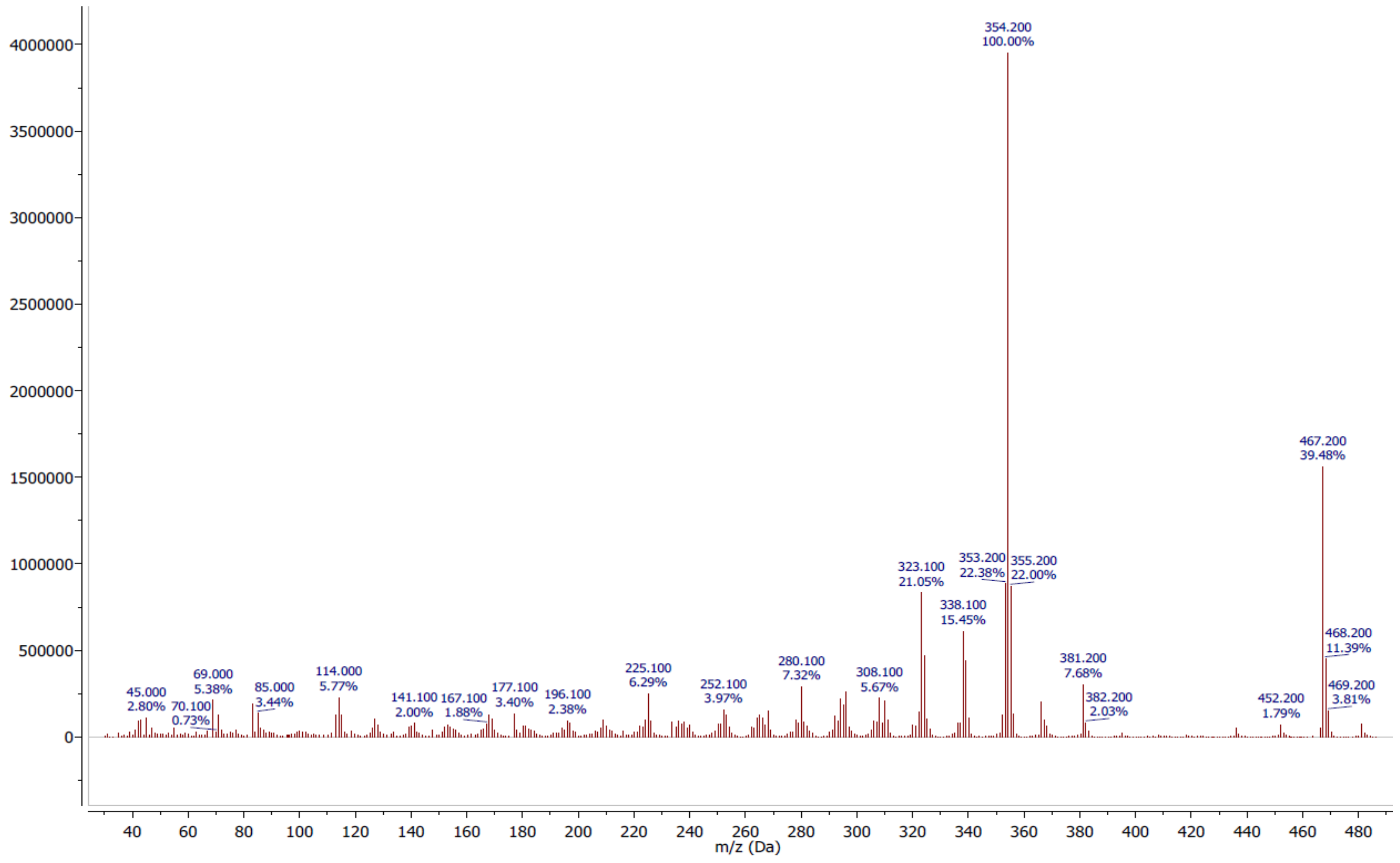

Figure 47s. Mass spectrum of B4

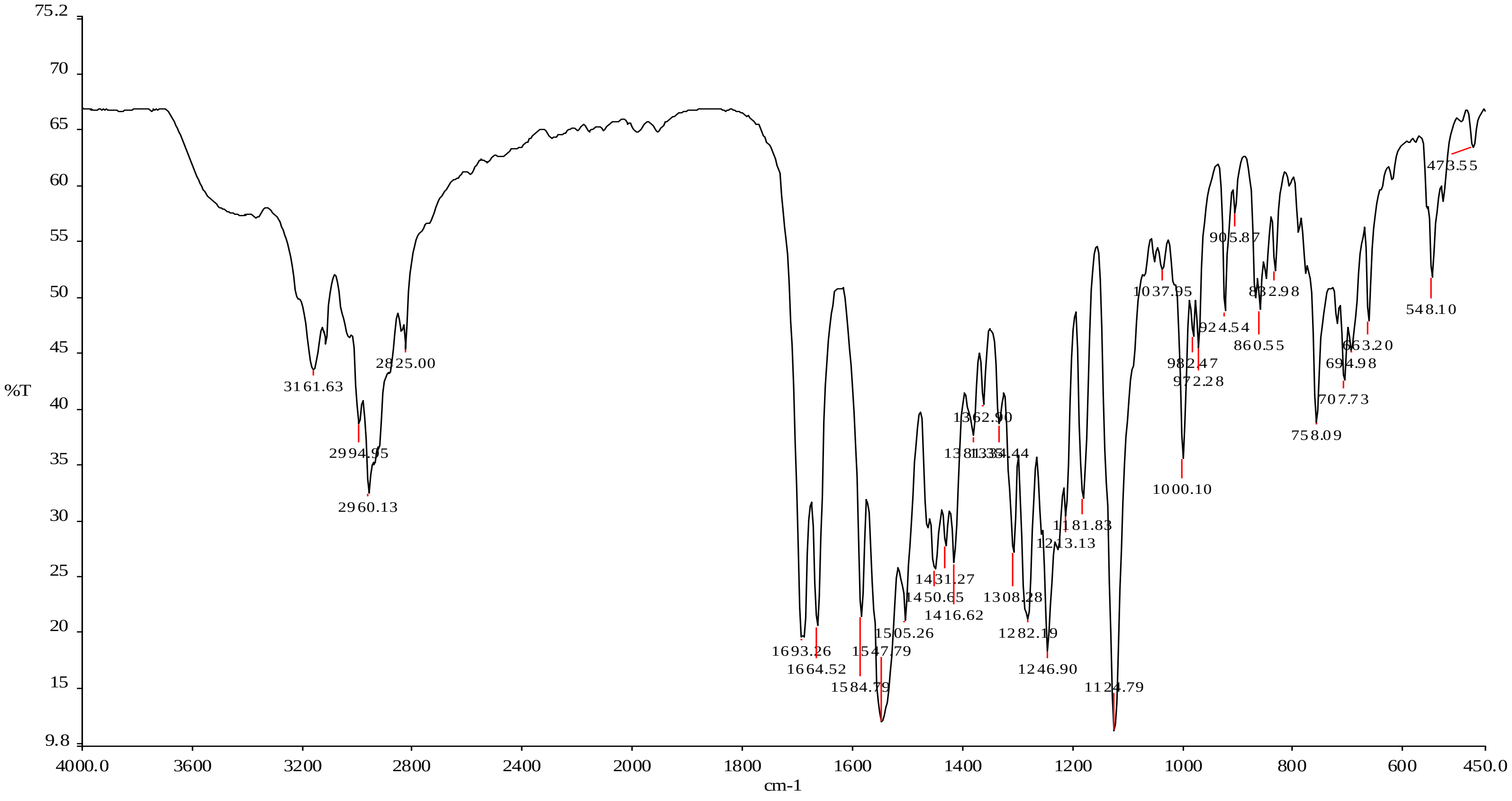

Figure 48s. IR spectrum of B4

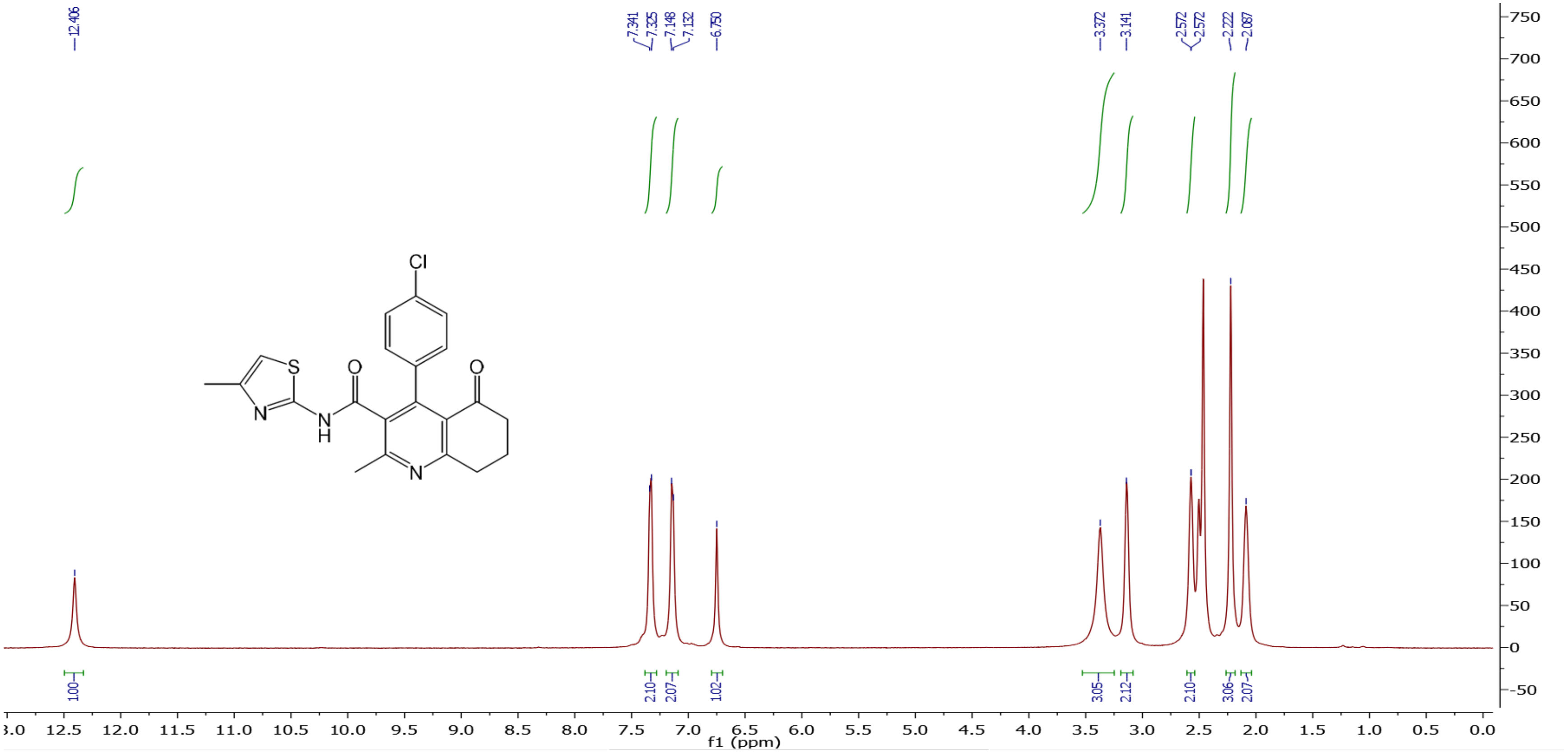

Figure 49s. ^{1}H NMR spectrum of B5

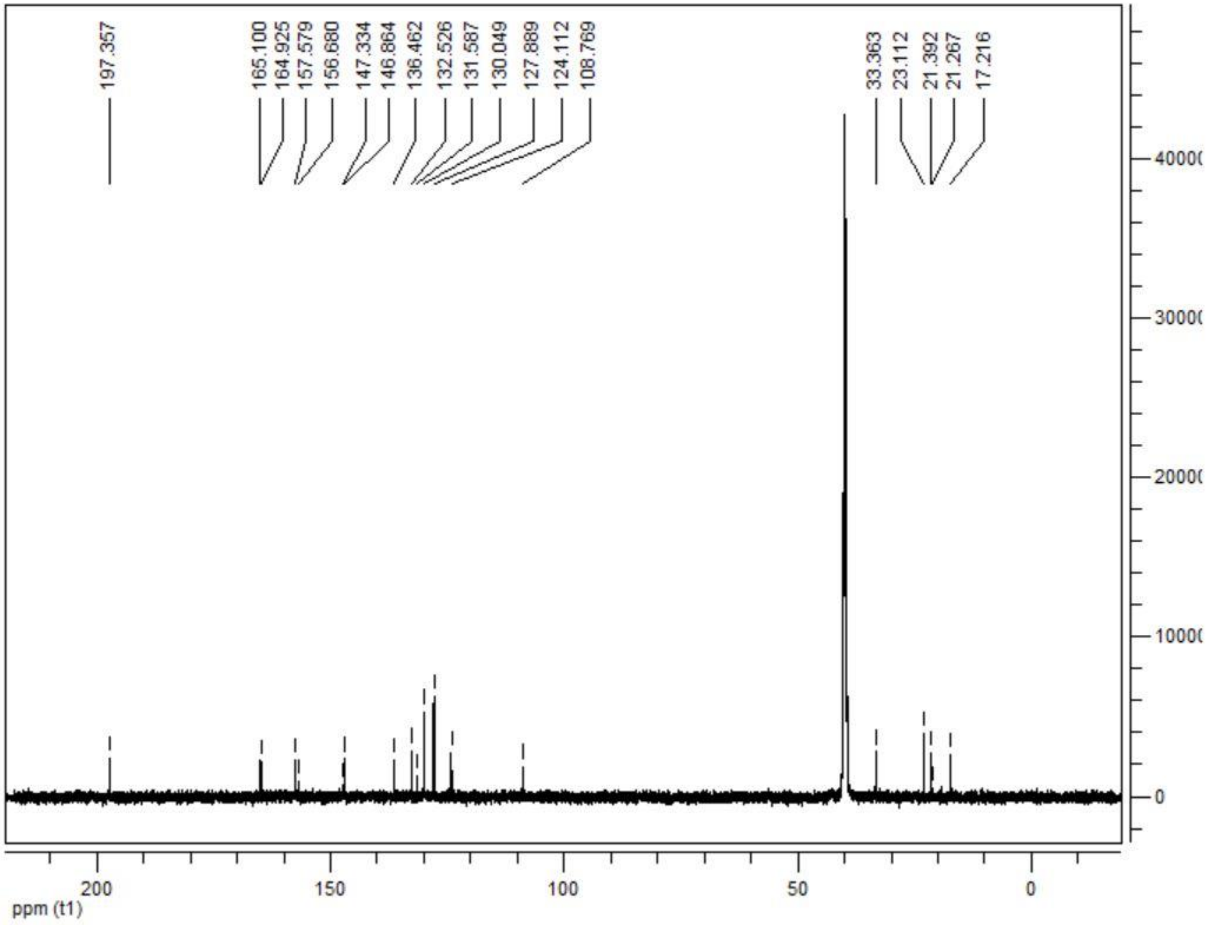

Figure 51s. ^{13}C NMR spectrum of B5

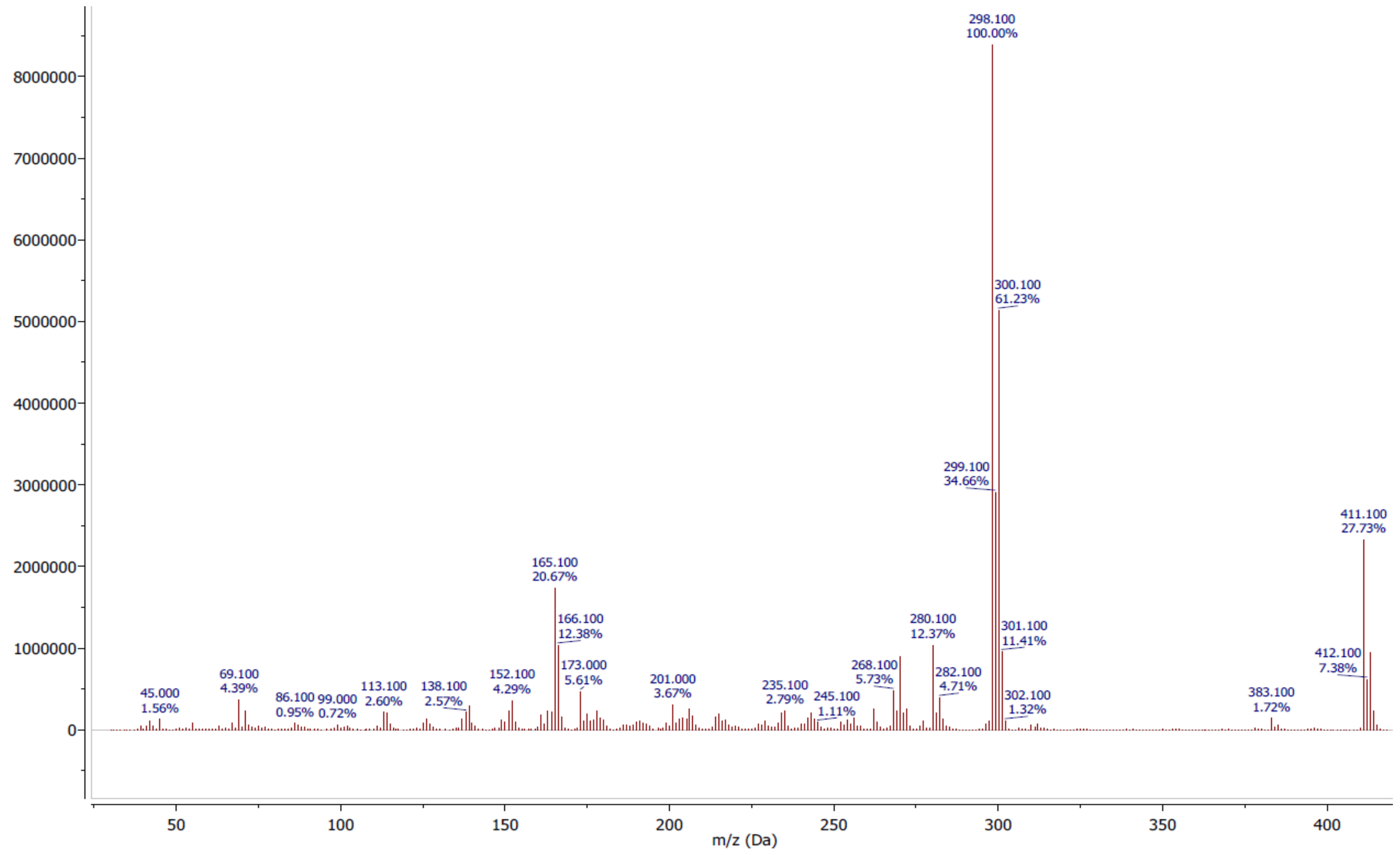

Figure 52s. Mass spectrum of B5

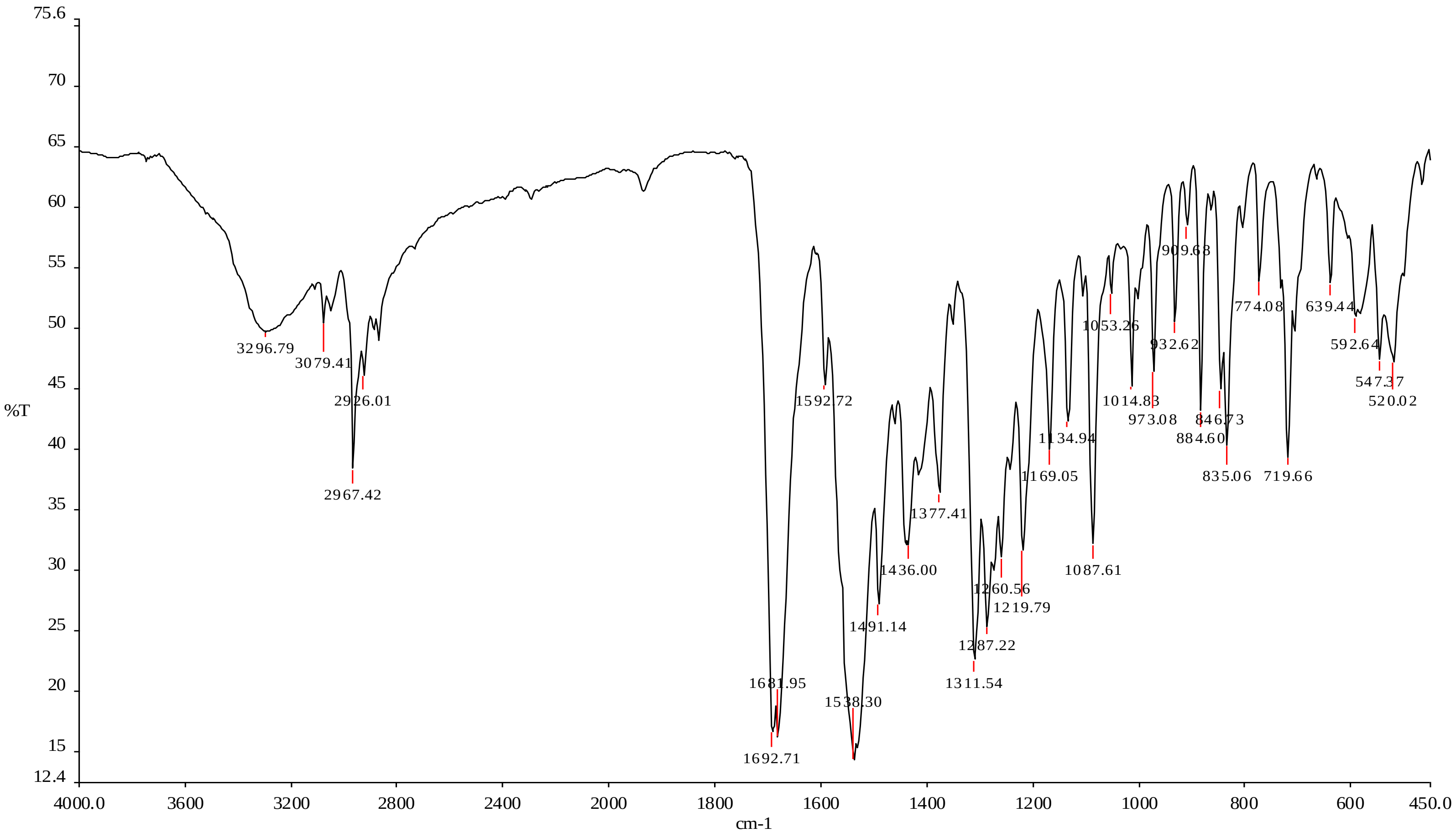

Figure 53s. IR spectrum of B5

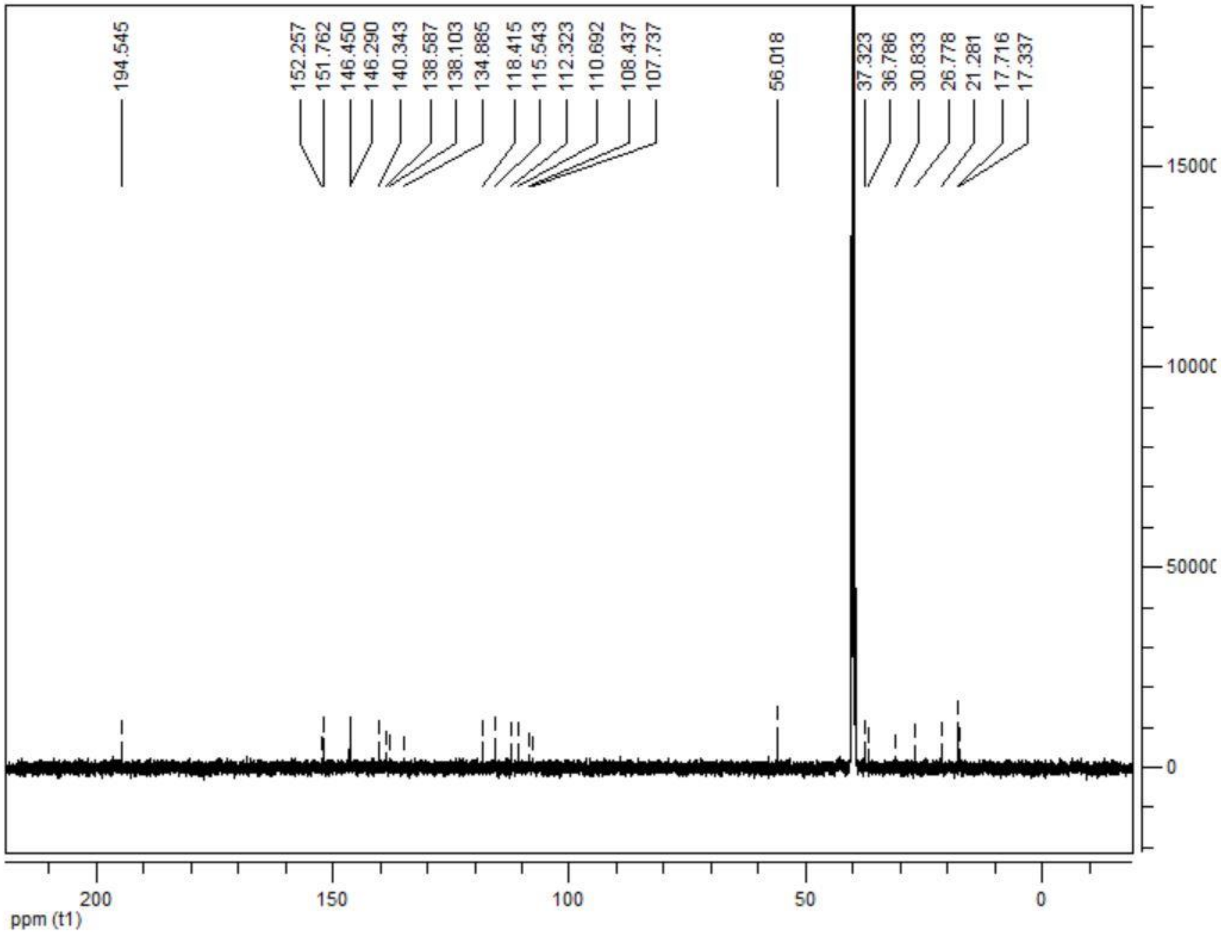

Figure 54s. ^{13}C NMR spectrum of B6

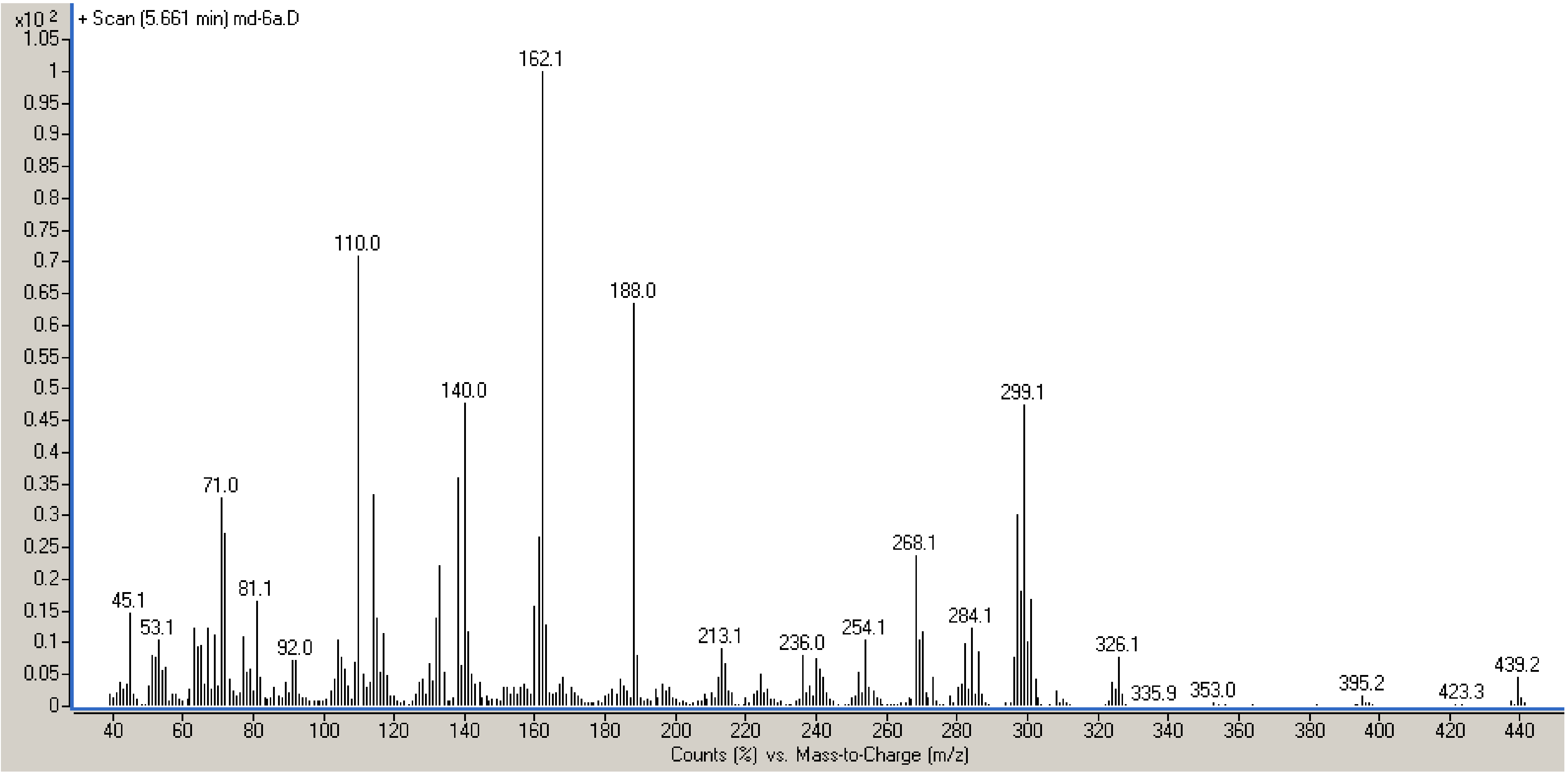

Figure 55s. Mass spectrum of B6

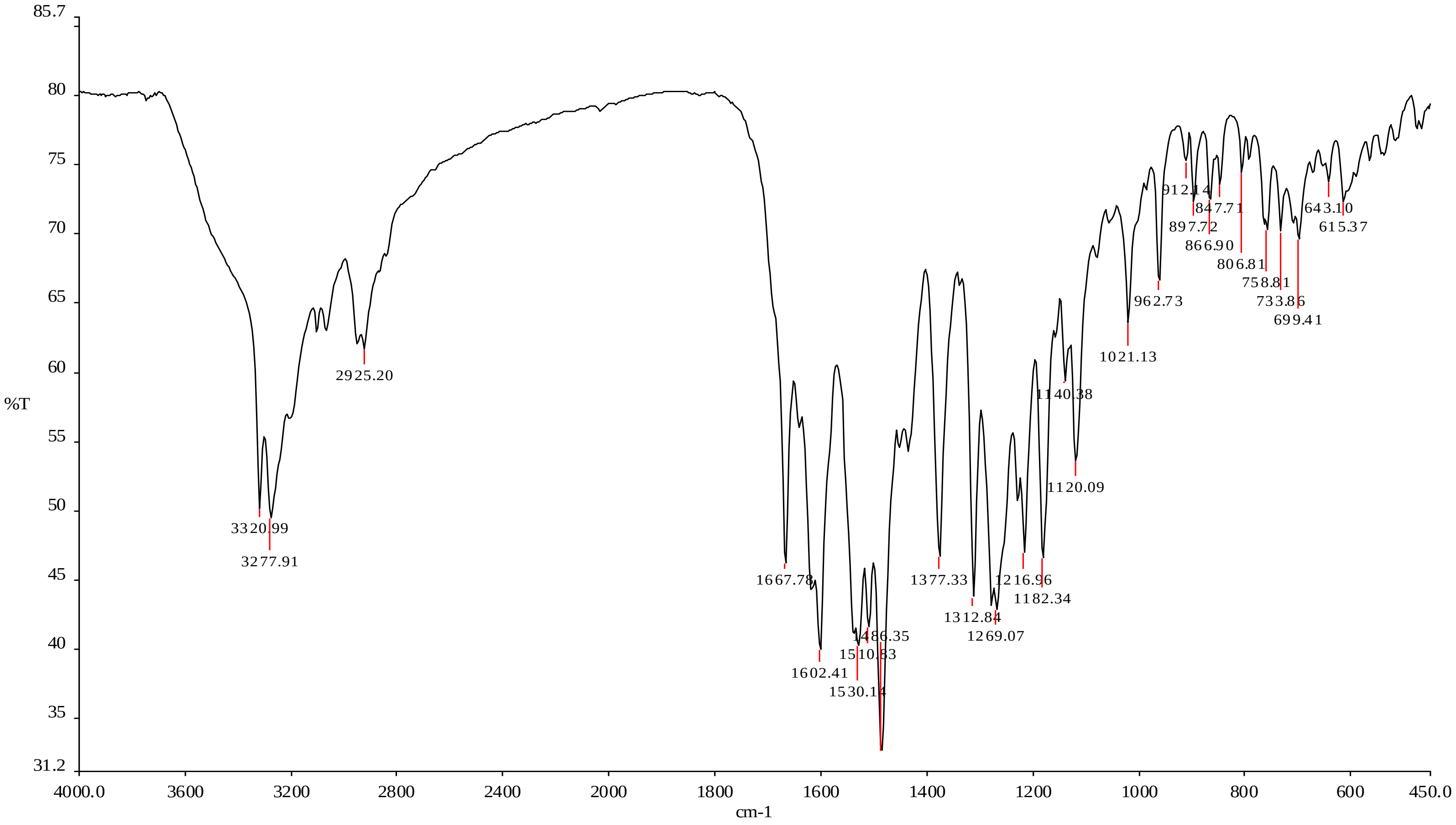

Figure 56s. IR spectrum of B6

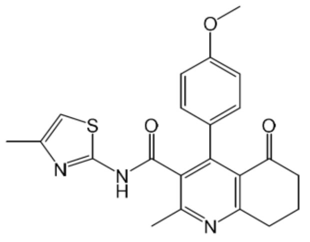

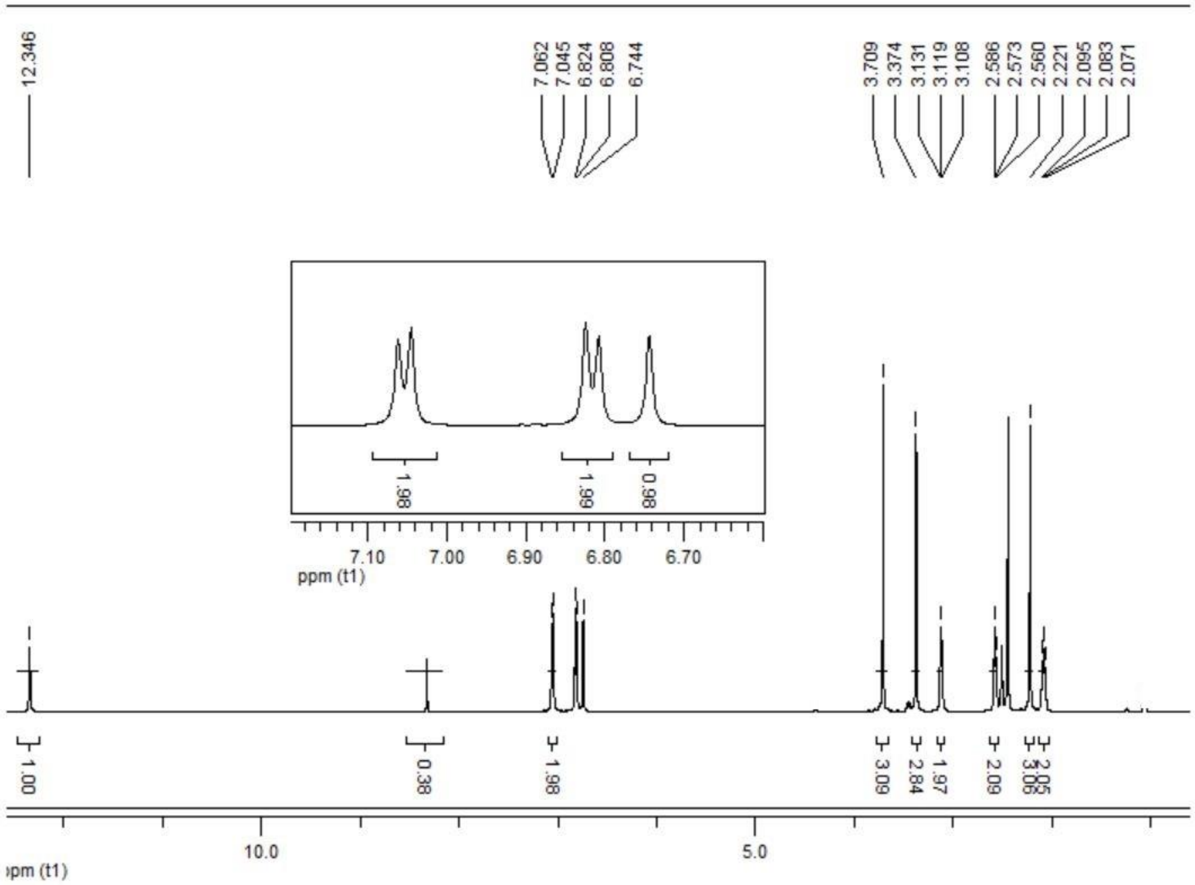

Figure 57s. ^{1}H NMR spectrum of B7

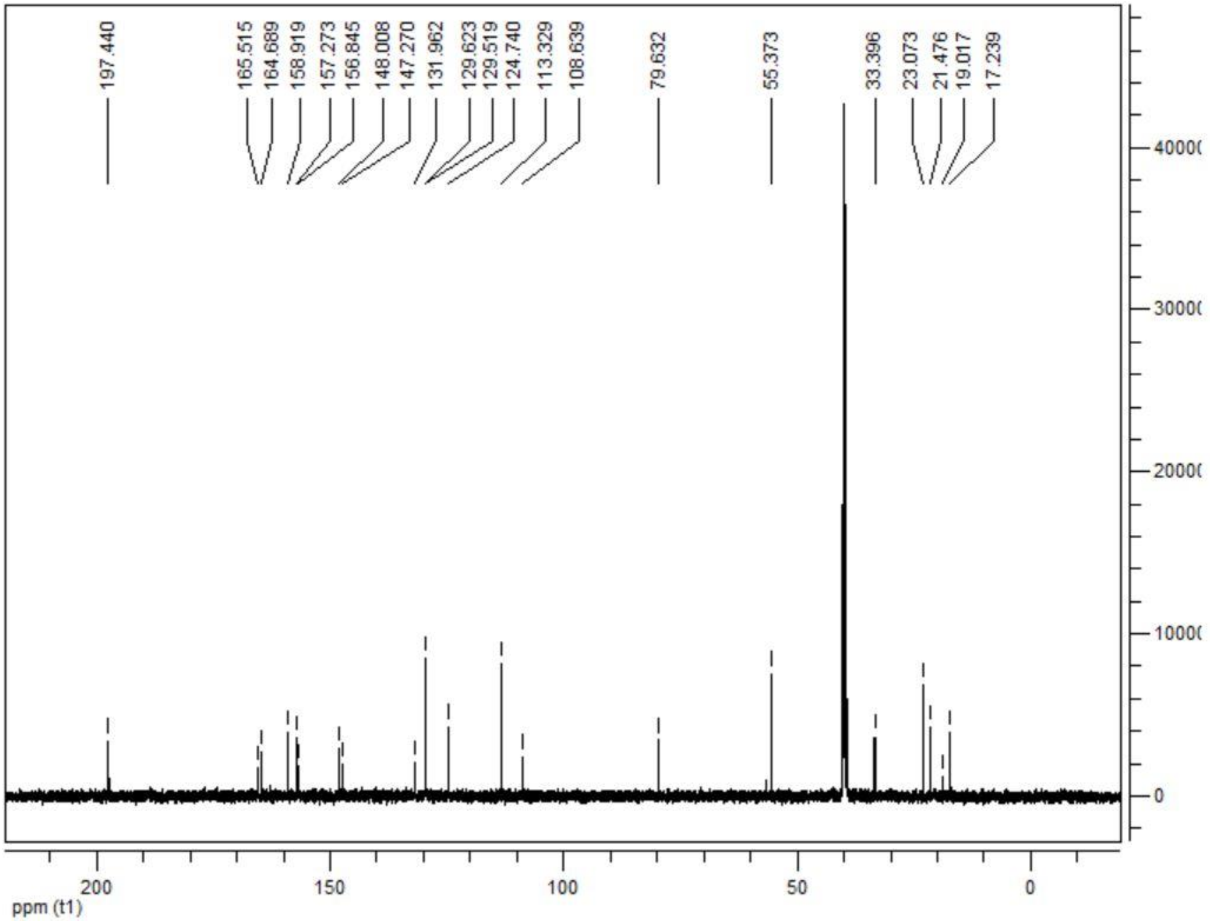

Figure 58s. ^{13}C NMR spectrum of B7

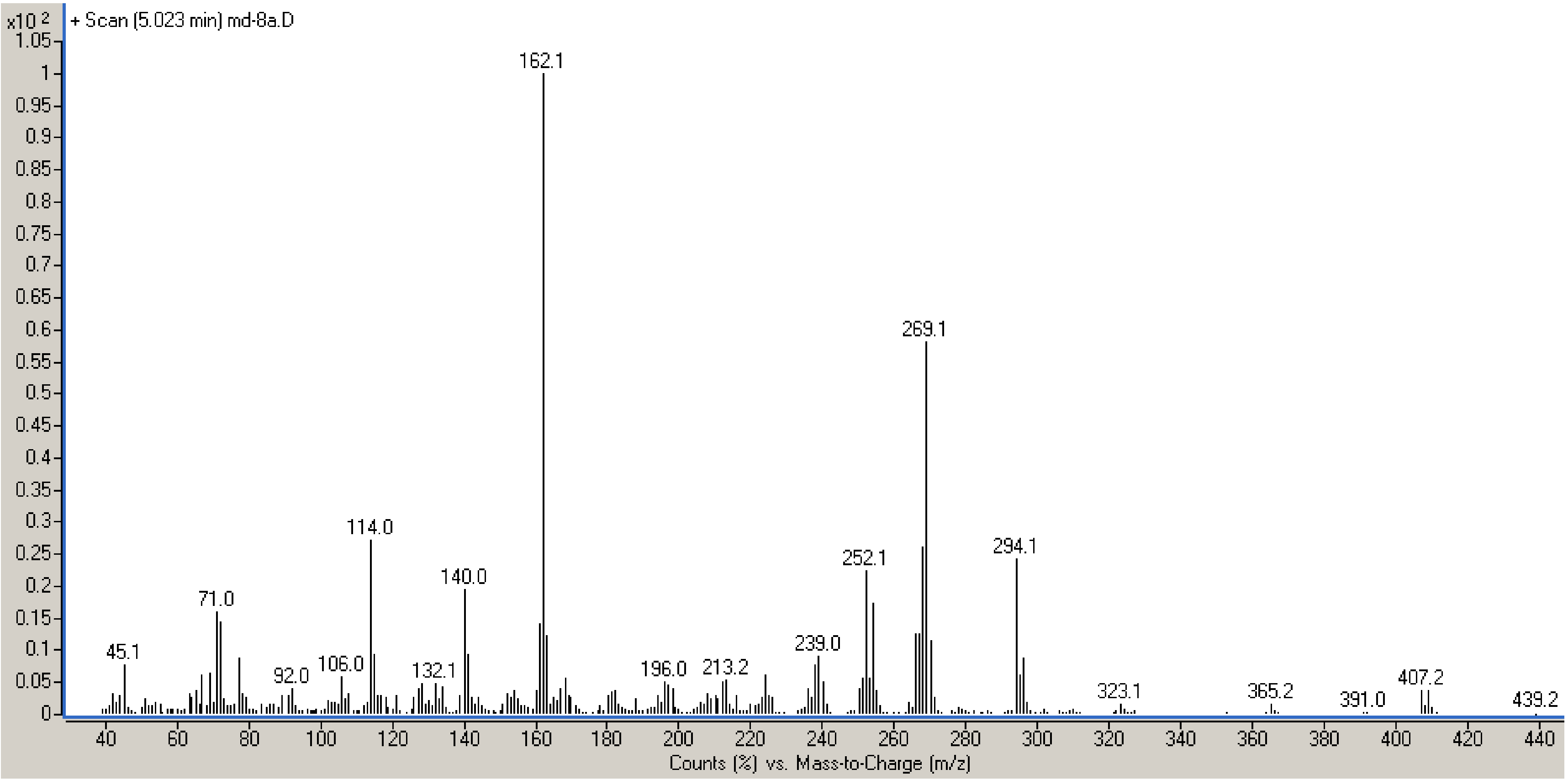

Figure 59s. Mass spectrum of B7

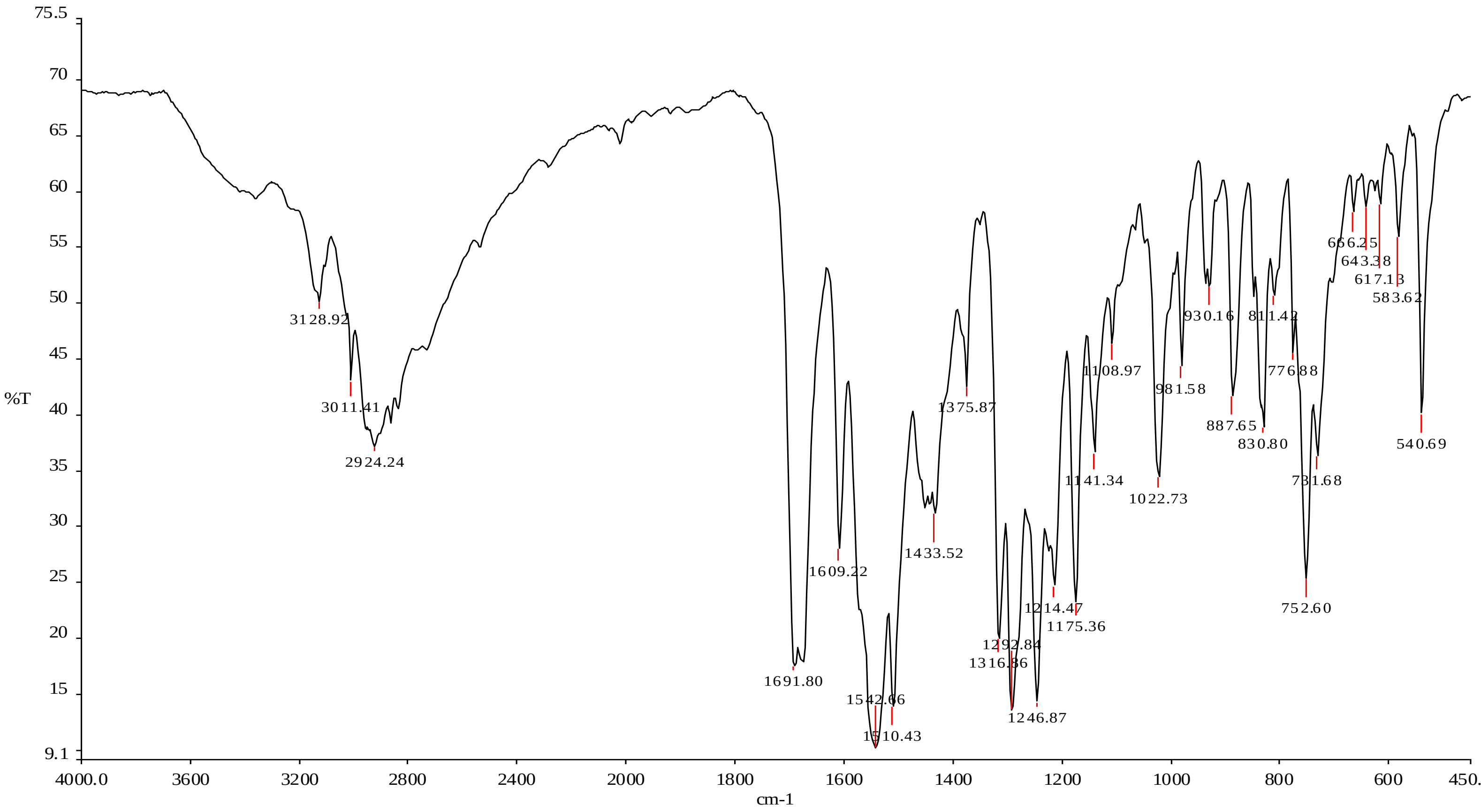

Figure 60s. IR spectrum of B7

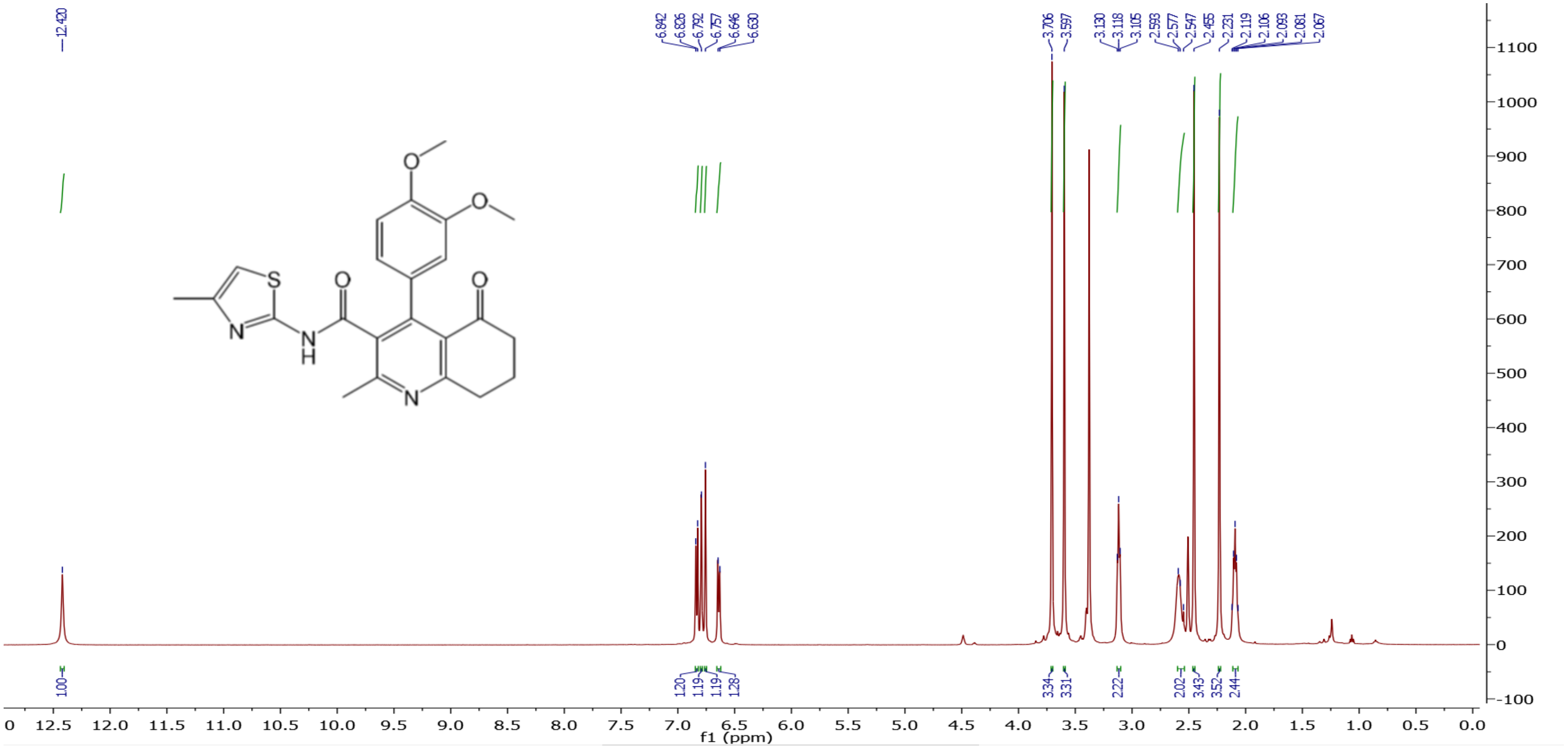

Figure 61s. ^{1}H NMR spectrum of B8

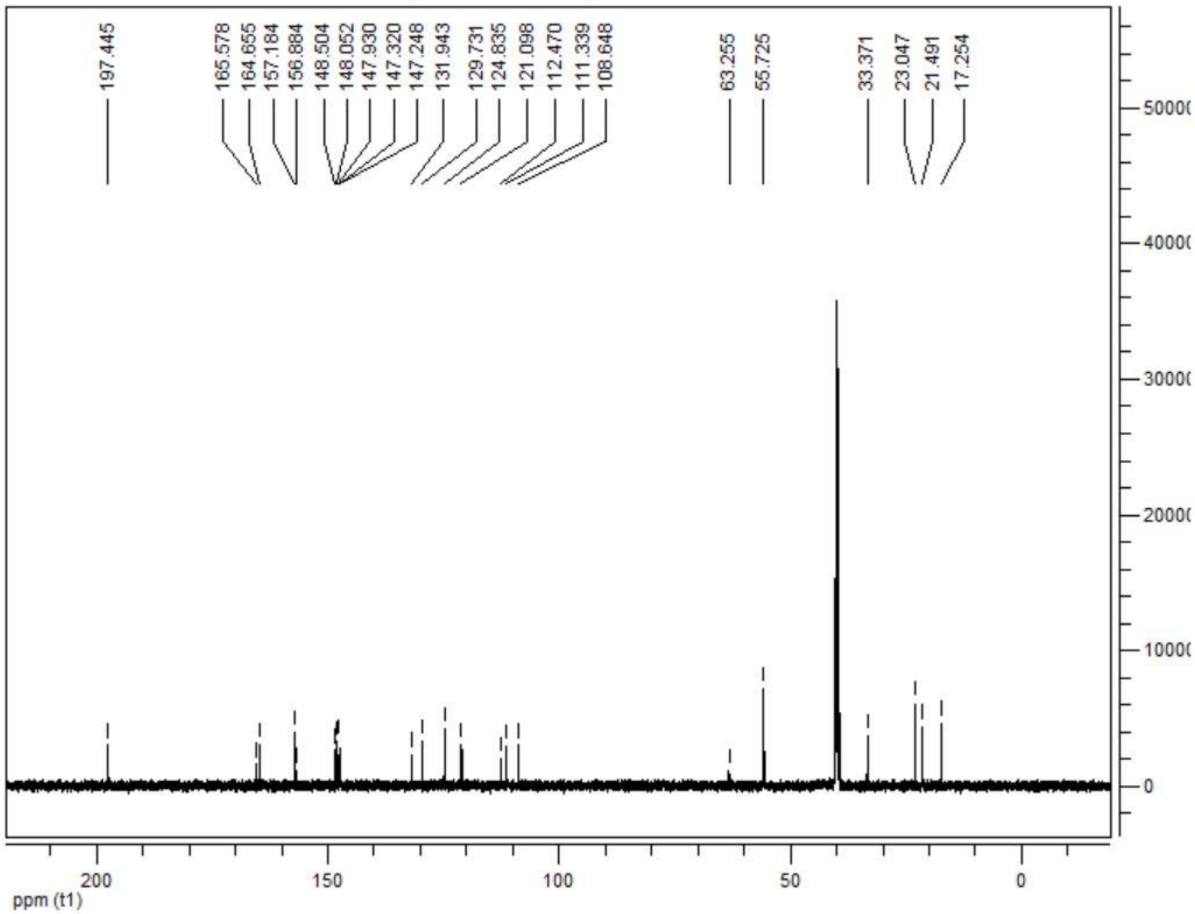

Figure 62s. ^{13}C NMR spectrum of B8

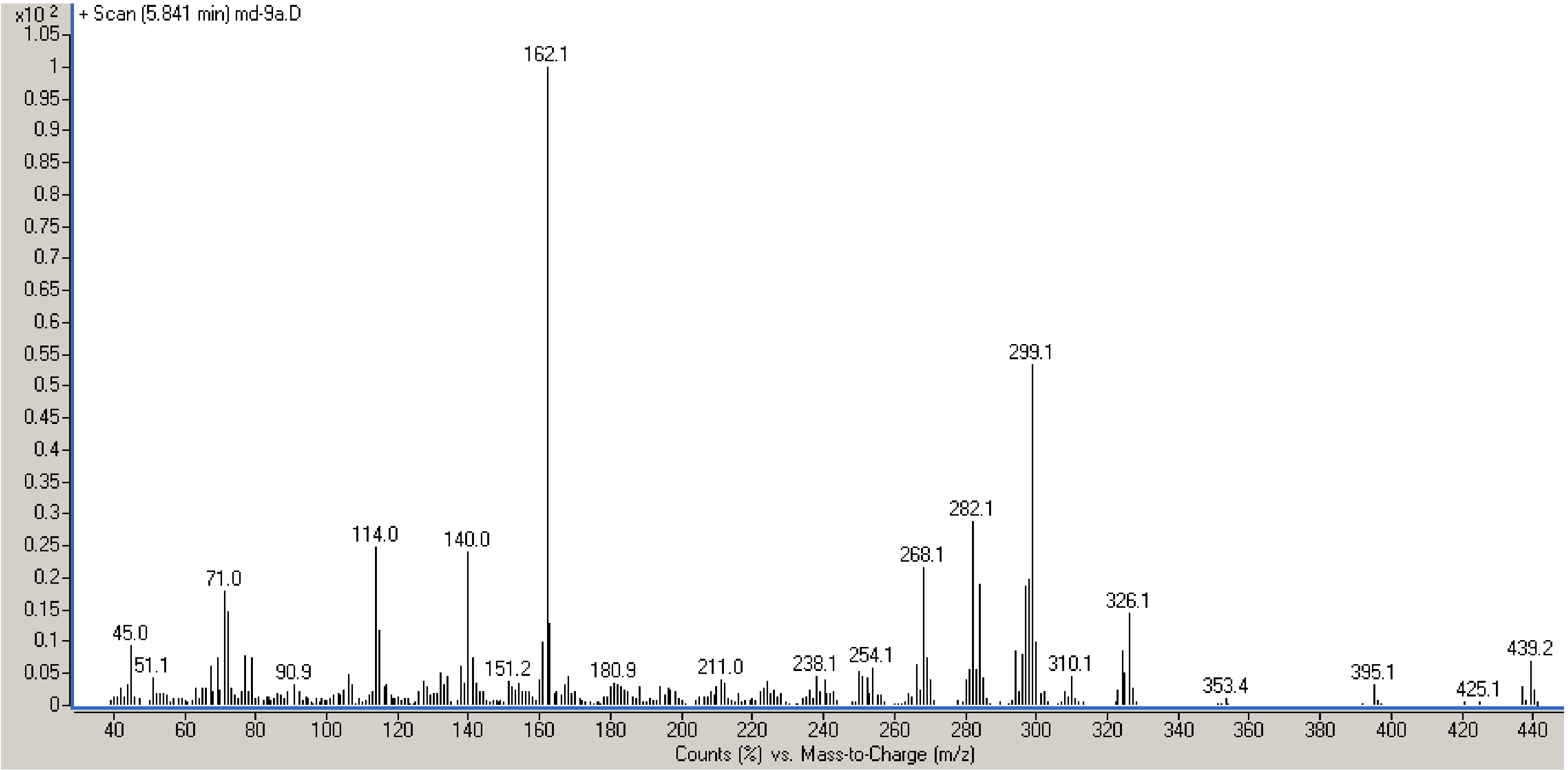

Figure 63s. Mass spectrum of B8

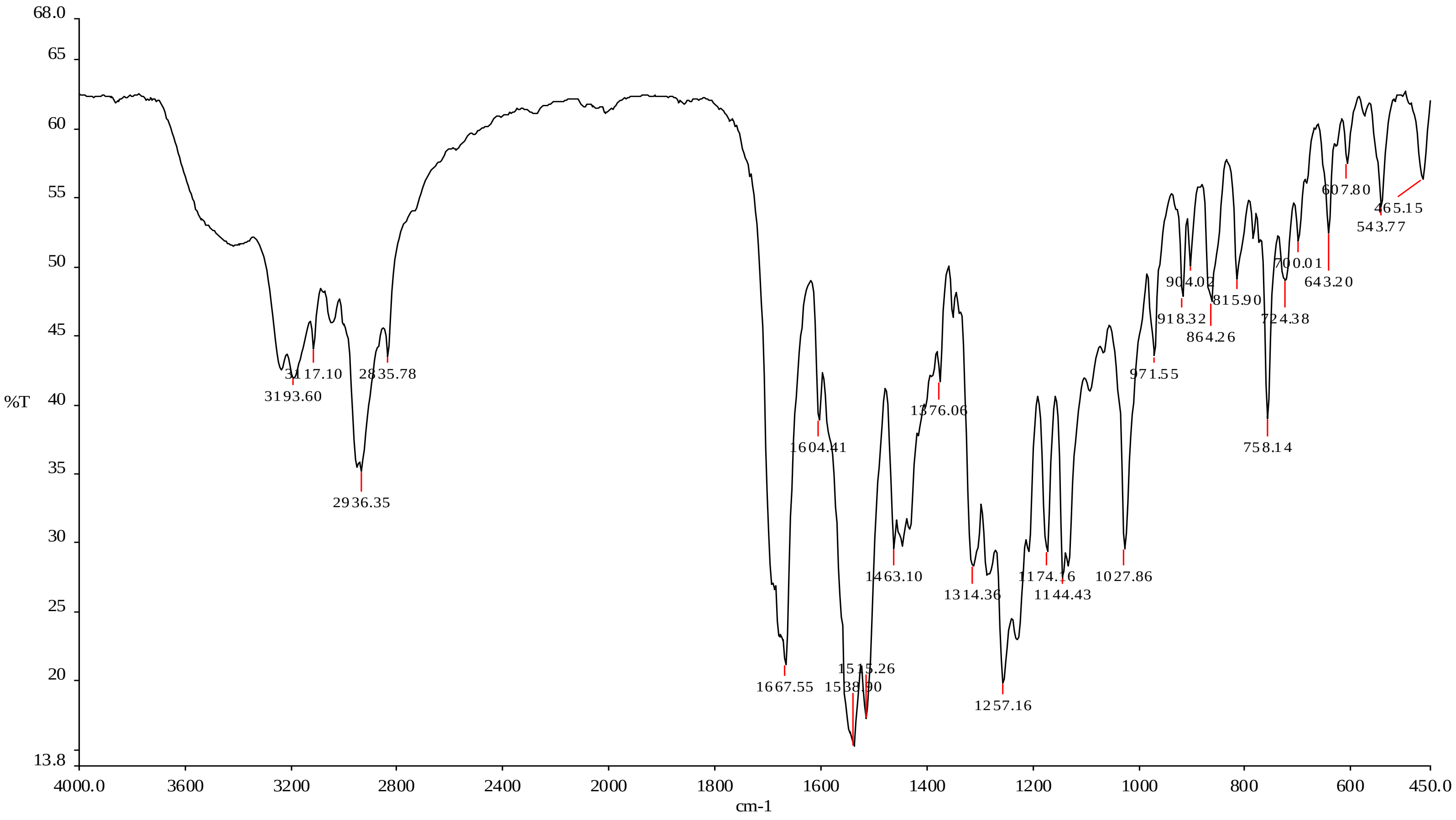

Figure 64s. IR spectrum of B8
